# Nepotism vs. intergenerational transmission of human capital in Academia (1088–1800)

**DOI:** 10.1007/s10887-024-09244-0

**Published:** 2024-06-29

**Authors:** David de la Croix, Marc Goñi

**Affiliations:** 1 IRES/LIDAM, UCLouvain and CEPR, Place Montesquieu 3, B-1348 Louvain la Neuve, Belgium; 2 Department of Economics, University of Bergen and CEPR, Fosswinckels gate 14, 5007 Bergen, Norway

**Keywords:** Intergenerational mobility, Human capital transmission, Nepotism, Universities, Upper-tail human capital, Pre-industrial Europe, C31, E24, J1

## Abstract

We have constructed a comprehensive database that traces the publications of father–son pairs in the premodern academic realm and examined the contribution of inherited human capital versus nepotism to occupational persistence. We find that human capital was strongly transmitted from parents to children and that nepotism declined when the misallocation of talent across professions incurred greater social costs. Specifically, nepotism was less common in fields experiencing rapid changes in the knowledge frontier, such as the sciences and within Protestant institutions. Most notably, nepotism sharply declined during the Scientific Revolution and the Enlightenment, when departures from meritocracy arguably became both increasingly inefficient and socially intolerable.

**Supplementary Information:**

The online version contains supplementary material available at 10.1007/s10887-024-09244-0.

## Introduction

Universities and scientific academies were instrumental in shaping major historical developments such as the Commercial Revolution, the Scientific Revolution, and the Enlightenment.^1^ Yet, despite their critical roles, these institutions have often faced criticism for clinging to outdated paradigms, commodifying educational credentials, and practicing nepotistic hiring. Historical data from 1088 to 1800 indicates that nearly 5% of scholars found employment at the same universities or academies as their fathers, often producing less scholarly work compared to their peers. This trend suggests that nepotism may have enabled less qualified individuals to secure positions based on familial ties rather than merit, adversely affecting knowledge formation. Nevertheless, the prevalence of family dynasties in high-skill fields underscore, in contrast, the importance of inherited human capital in sectors where talent is scarce.^2^

Disentangling inherited human capital from nepotism is important as their social and economic implications are fundamentally different: while dynasties based on inherited human capital can reflect meritocracy and increase productivity, nepotism leads to a misallocation of talent.^3^ Such misallocation is specially harmful in high-talent markets (Murphy et al., 1991; Hsieh et al., 2019), affecting the production of ideas, upper-tail human capital, technological progress, and economic growth (Mokyr, 2002).

However, measuring the human capital transmitted from parents to children, and separating it from nepotic practices is challenging in several respects. Inherited human capital endowments are unobservable by nature and are only imperfectly reflected into outcomes such as occupation, earnings, or performance. Recent studies suggest that this introduces a large measurement error bias which can severely attenuate intergenerational elasticity estimates and mask the true rate of occupational persistence.^4^ In turn, nepotism reflects parents’ social connections that allow sons to get jobs ahead of better-qualified candidates. These entry barriers are not only hard to quantify, but also introduce a different bias—selection—as sons of insiders are selected into top occupations under different criteria than outsiders. Finally, microdata with direct parent–child links is hard to come by in historical settings. Previous estimates on the parent–child transmission of human capital and social connections are limited to modern settings or rely on surname pseudo-links to study its evolution over centuries.^5^

In this paper, we quantify nepotism and inherited human capital in academia over seven centuries. We do so by building a comprehensive dataset with direct links between 1837 sons and fathers in 116 universities and 63 academies from 1088 to 1800. We then measure their scientific output using 4,106,901 library holdings by or about each scholar that are held in more than 10,000 libraries today (henceforth, publications). To separate inherited human capital from nepotism, we develop a new structural method which addresses the measurement error and selection biases described above by exploiting two sets of moments: *(i)* correlations in publications across generations—a standard moment to estimate intergenerational elasticities; and *(ii)* differences in the marginal publications’ distribution between the set of fathers and the set of sons—a novel moment. Our findings indicate that nepotism declined at times when the misallocation of talent incurred greater social costs, such as the Scientific Revolution and the Enlightenment, and in fields experiencing rapid changes in the knowledge frontier, particularly in the sciences and within Protestant institutions. Family dynasties did not disappear, but they became meritocratic, emerging mostly as a result of the human capital, knowledge, and other productive endowments that children inherited from their parents. We find that such upper-tail human capital endowments were inherited with an elasticity of 0.6–0.65—a higher estimate than suggested by simple parent–child correlations in publications, but lower than previous long-run estimates relying on surname pseudo-links.

**Fig. 1:**
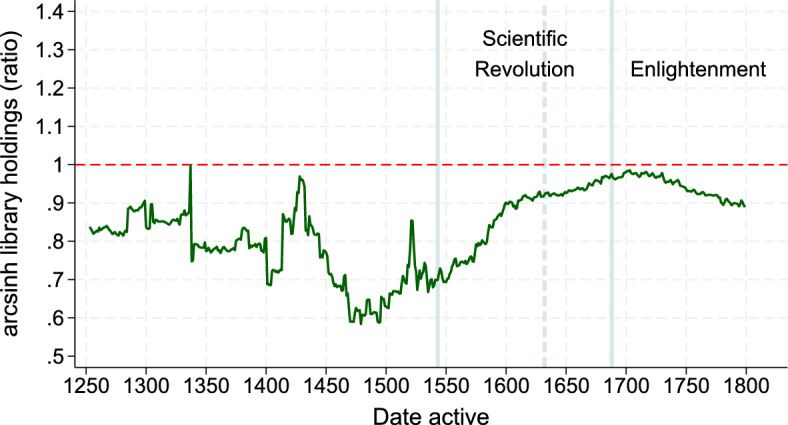
Publications of sons of scholars relative to outsiders over time. *Notes* The sample is 20,500 scholars from institutions with complete and broad coverage who are listed in Worldcat. The figure shows the ratio of the library holdings of the average son to the library holdings of the average outsider over time. We use the hyperbolic sine transformation of the number of library holdings. “Sons” means sons in the same institution as fathers. Trends are based on a 50-years moving average (100-years moving average before 1400). We exclude outliers (99-percentile) for both groups. We omit trends before 1250 because the sample restrictions above reduce to 5 the number of sons before 1250

Figure 1 illustrates our main findings. It shows the number of library holdings in modern libraries by or about an average scholar’s son relative to an average outsider in academia from 1250 to 1800, based on 20,500 scholars listed in WorldCat. The ratio is always below one, suggesting that scholar’s sons were less productive than outsiders. However, the figure also shows that their publications converged over time. In detail, the publications of scholars’ sons were 80% those of outsider scholars until 1400, and as low as 60% around 1500.^6^ This pattern reversed with the start of the Scientific Revolution and, by 1632—when Galileo’s *Dialogue* was published, the average scholar’s son published close to 90% as much as the average outsider. At the dawn of the Enlightenment (1687–1800) we observe no differences between the scientific output of scholars’ sons and outsiders. These trends motivate our main finding that nepotism faded in times of rapid scientific advancement when the misallocation of talent incurred greater costs, like the Scientific Revolution.

That said, the figure also highlights some of the empirical challenges associated with disentangling nepotism from inherited human capital. Comparisons between scholars’ sons and outsiders conflate the negative effect of nepotism with the positive effect of human capital transfers from fathers to sons. Specifically, one cannot simply assume that, absent nepotism, sons of scholars and outsiders would produce the same output. Absent nepotism, we would expect sons of scholars to publish as much as outsiders (ratio$=$1) if human capital reverted to the mean after just one generation, and more than outsiders (ratio>1) in the more realistic scenario where human capital transfers from fathers to sons mattered for a scholar’s research output. The productivity gap, hence, depends on the elasticity at which parents transfer their human capital to their sons. In other words, to disentangle nepotism from human capital transfers and to quantify their relative importance over time, it is crucial to observe father–son links, infer the father’s human capital endowments, and the elasticity at which these are transferred to their sons. In most empirical settings, there is no information about the parents of outsiders, their occupations (here, outside academia), or human-capital proxies comparable across occupations (here, a measure akin to publications in other occupations). Without this information, it is virtually impossible to estimate neither the inherited human capital of outsiders nor its importance relative to nepotism for the outsider-son productivity gap. We overcome these issues by exploiting direct parent–child links and comparing scholars’ sons and fathers, and validate our results *ex post* with comparisons between outsiders and scholars’ sons.

Our first contribution is to build a new dataset with direct parent–child links in premodern academia. We build our dataset using hundreds of secondary sources on university professors and members of academies, such as university catalogues, books on the history of each university, and compendia of professors. We establish and verify direct family links by matching each scholar to old biographical dictionaries and online encyclopedias (e.g., *Allgemeine Deutsche Biographie*, *Treccani*, and Dictionary of National Biography). Given the completeness of our sources, we collect the universe of father–son pairs in most institutions. We measure their publications using WorldCat—a comprehensive online catalogue of modern libraries worldwide. Specifically, we define publications as their library holdings in modern libraries, which capture the size as well as the long-run relevance of a scholar’s scientific output.

We begin our analysis by documenting two stylized facts for families of scholars. The first fact is that a scholar’s publications strongly depend on his ancestor’s publications. The father–son correlation on the intensive margin is 0.375, and the grandfather-grandson correlation is stronger than predicted by iterating the father–son correlation. The second fact is that there are large differences between the marginal publications distribution of the set of fathers and the set of sons—that is, between first-generation scholars and subsequent insiders. The fathers’ distribution first-order stochastically dominates the sons’ distribution, and differences are largest at the bottom.

We show that these two facts cannot be reconciled with standard intergenerational models based solely on the transmission of human capital (Becker and Tomes 1979, 1986). Fact 1 implies that the underlying human-capital endowments determining publications were strongly transmitted from parents to children, and hence, that the advantages and disadvantages of ancestors vanished at a slow rate, i.e., a slow rate of mean reversion. In contrast, Fact 2 implies a fast rate of mean reversion, as the set of sons has a substantially worse publication record than the set of fathers. These two apparently contradictory facts can be reconciled by extending the standard intergenerational framework with social connections that can result in nepotism. Nepotism allows scholars’ sons to become scholars even when their human capital is lower than that of the marginal father, generating differences at the bottom of the distribution (Fact 2) even when publications on the intensive margin slowly revert to the mean across generations (Fact 1).

Our second contribution is to develop a general method to disentangle inherited human capital from nepotism, and to use the two facts described above to estimate their relative importance in premodern academia. Our method recovers the intergenerational human capital elasticity from the father–son correlations in publications; and nepotism from the excess distributional differences between the set of fathers and sons, net of the effect of mean reversion in human capital. Formally, we structurally estimate the parameters of a first-order Markov process of human capital transmission (Clark & Cummins, 2015; Braun & Stuhler, 2018), extended to account for nepotism.

In detail, our model economy consists of a population of potential scholars whose unobserved human capital is transmitted from fathers to sons with an elasticity of $\beta$. Potential scholars with high human capital endowments become scholars, but there is a selection bias: the selection criterium for scholars’ sons can be different because of nepotism. We define nepotism as the share of scholars’ sons who would not have become scholars under the same criterium as a first-generation scholar. For selected scholars, the unobserved human capital endowments are transformed into an observed outcome, publications, with measurement error noise. The two sets of moments characterizing Facts 1 and 2 can be used to identify the deep parameters of this model: Father–son correlations in observed outcomes are a standard moment to characterize the rate $\beta$ at which human capital is transmitted from fathers to sons.^7^ Because publications are a noisy proxy of human capital, we also use correlations between grandparents and grandchildren, a method proposed by Lindahl et al. (2015) and Braun and Stuhler (2018) to correct for measurement error. Finally, we exploit that when the distributional differences between the set of fathers and sons are larger than predicted by the rate of reversion to the mean, it reflects that parents and children are selected under different criteria, i.e., nepotism. This should be specially visible at the bottom of the distribution, that is, close to the selection threshold where nepotism is binding. The excess distributional differences, net of the effect of mean reversion, can hence be used to identify nepotism. We use the Simulated Method of Moments to obtain estimates for the intergenerational elasticity of human capital, $\beta$, and for nepotism by minimizing the distance between these empirical moments and their simulated counterparts.

Our third contribution is to quantify nepotism in premodern academia. We find that, between 1088 and 1543, 48.8 of scholars’ sons would not have become scholars under the same criteria than their fathers. This nepotism estimate declined to 20.35% in the Scientific Revolution (1543–1687) and to 8.3% in the Enlightenment (1687–1800).

Importantly, we examine some of the historical processes behind the decline in nepotism around the Scientific Revolution. We show that nepotism was less prevalent in areas experiencing rapid changes in the knowledge frontier. Specifically, we use data from De la Croix (2021) on publications of all known scholars in 1500–1800 to calculate the yearly growth rate of publication by six fields of study in catholic and protestant universities. Our estimates show that nepotism was 9.2% among scholars entering academia in a time, place, and field of study which had experienced rapid changes in the knowledge frontier in the previous quarter century, and 25.3% among those entering academia in stagnant times, places, and fields. This suggests that the decline in nepotic practices in academia after the Scientific Revolution is explained by an increase in their costs: In eras of rapidly changing knowledge frontiers, the mismatch between talents and occupation becomes more costly, exceeding the benefits from the transmission of specific human capital from parents to children (Galor & Tsiddon, 1997).

In addition, consistent with the historical evidence, we document that a key mechanism behind the decline of nepotism was the foundation of modern, meritocratic institutions instead of structural reforms in existing institutions. To do so, we estimate our model separately for institutions established before vs. after the start of the Scientific Revolution, and show that the share of nepotic sons was more than 50% smaller in new institutions—such as the universities of Leiden, Jena, or the French Royal Academy of Sciences—than in the old institutions—such as the universities of Cambridge or Bologna.

Altogether, this suggests that low levels of nepotism are associated with periods of buoyant scientific advancement. To the extent that this decline in nepotism reflects a broader decline in favoritism towards acquaintances and other societal changes reducing barriers to entry in academia, our findings suggest that meritocracy was complementary with Europe’s scientific advancements before the Industrial Revolution.

Our fourth contribution is to provide estimates on the intergenerational elasticity of upper-tail human capital in premodern Europe. We document that human capital endowments were transmitted with an elasticity of 0.63within father–son pairs in academia. This estimate is higher than suggested by father–son elasticities in observed outcomes, confirming previous findings that two-generation estimates understate the rate at which inequalities persist over the very long run. Yet, our estimate is in the lower range of elasticities obtained via multiple generations, group-averages, or the informational content of surnames—three methods that ignore the transfer of social connections that can lead to nepotism. Specifically, elasticities obtained via multiple generations in our data are close to the 0.8–0.9 range estimated by Clark (2015). Hence, in settings with widespread nepotism, the standard multi-generational methods in the literature overstate the persistence of inherited endowments, skills, etc. which affect children’s outcomes. Furthermore, our findings do not support Clark’s hypothesis that the rate of persistence is constant through historical periods and, hence, that it reflects the transfer of genetic endowments.

In addition, we extend our analysis to examine heterogeneous effects. We find evidence of nepotism for 5–6.6% of scholars’ sons in Protestant and for 29.4% in Catholic universities and academies. Catholic institutions relied more heavily on intra-family human capital transfers. We show that these differences partly explain the divergent path of Catholic and Protestant universities after the Reformation. We also document that nepotism was higher in law and medical faculties than in sciences, for sons appointed before their father’s death, and for sons in the same field as their fathers. In addition, we conduct various robustness checks. First, we show that our estimates are not driven by selective reporting of father–son links in the sources used to build our data. Our estimates are robust to restricting the sample to sources covering all scholars in an institution, and hence, where we effectively identify the universe of father–son pairs. Second, we show that our findings—based on comparisons between scholars’ sons and fathers—are consistent with comparisons between scholars’ sons and outsiders. Third, we validate our identification strategy with a falsification test. We consider fathers and sons appointed at different institutions where, *ex ante*, we expect less nepotism. Consistently, we estimate a nepotism parameter of zero. This strongly suggests that our estimates do not conflate nepotism with other elements of the hiring process (e.g., information frictions) or with broader trends outside academia to which both our baseline and validation sample are exposed. Finally, we examine the robustness of our results to stationarity assumptions, drawing shocks from fat-tailed distributions, using unique works instead of library holdings, dropping library holdings on a scholar’s work written by a different author, allowing for better access to publishers for scholars’ sons, non-linearities human capital transmission, and father–son longevity differences.

Our paper can be seen as integrating three strands of literature. First, a number of studies have estimated the prevalence of nepotism in top professions.^8^ For modern academia, previous work has documented nepotism (Durante et al., 2011) and favoritism towards acquaintances (Zinovyeva & Bagues, 2015; Bramoullé & Huremovic, 2018) or scholars with home-ties (Fisman et al., 2018). Our paper is the first to quantify nepotism in premodern academia. Studies of favoritism in premodern organizations are scarce. An exception is Voth and Xu (2019), who find that promotions of connected British Navy officers reflected private information rather than favoritism. Methodologically, a common approach to estimate nepotism is to use natural experiments that alter the importance of connections to accessing jobs. Instead, our method allows to gauge the evolution of nepotism across time and space, beyond settings where such natural experiments are available.

Second, this study also contributes to a large literature on social mobility by providing the first estimate for the intergenerational elasticity of upper-tail human capital over centuries. While previous long-run elasticity estimates of wealth, earnings, and occupation rely on surname pseudo-links (Clark & Cummins, 2015; Barone & Mocetti, 2020; Häner & Schaltegger, 2024), our estimates are derived from true links across generations.^9^ This allows us to directly evaluate Clark (2015)’s hypothesis that latent endowments are transmitted from parents to children at a constant rate of 0.8–0.9 over the very long run. More generally, we show that to obtain reliable intergenerational elasticities it is important to jointly address both measurement error and selection bias. Traditional elasticities bundle transfers of unobserved human capital and social connections and, hence, cannot address both biases jointly. Measurement error has been addressed using multiple-generation links (Lindahl et al., 2015; Braun & Stuhler, 2018; Colagrossi et al., 2019), group-averages for siblings (Braun & Stuhler, 2018) and surnames (Clark & Cummins, 2015; Häner & Schaltegger, 2024), the informational content of surnames (Güell et al., 2015), or horizontal kinship ties (Collado et al., 2018). In line with these estimates, we find that inherited advantages are more persistent than what parent–child elasticities imply. That said, we show that, by ignoring the selection bias, these estimates can overstate the persistence of endowments like human capital or genetic advantages.^10^ Importantly, here we estimate an elasticity for a specific group, fathers and sons in academia, while some of the literature estimates cited above correspond to population elasticities. Although we discuss that selection may also affect such elasticities, our critique is most relevant for long-run estimates, which are often derived from selected samples, e.g., individuals leaving a will (Clark & Cummins, 2015) or ancestors and descendants that remain in one city (Barone & Mocetti, 2020; Häner & Schaltegger, 2024).

Third, upper-tail human capital, such as knowledge produced at universities and academies, has been deemed important for the rise of new Science (Mokyr (2002), Mokyr (2016), De la Croix et al. (2023)) and the Industrial Revolution (Galor and Moav (2002), Squicciarini and Voigtlander (2015)). Our contribution to this literature is to provide the first estimate of the intergenerational elasticity of upper-tail human capital before the Industrial Revolution. We find a slower rate of mean reversion after the Scientific Revolution. This lends credence to Galor and Moav (2002) and Galor and Michalopoulos (2012), who show that natural selection of growth-promoting traits (e.g., upper-tail human capital) is more likely when parents pass on such traits with a high probability. In addition, we show that periods of rapid scientific advancement were associated with less nepotism in universities and academies. This provides empirical support to the hypothesis by Greif (2006) and De la Croix et al. (2018) that, in pre-industrial Europe, the dissemination of knowledge in corporations was not slowed down by family networks.

Our finding that nepotism becomes more costly when the knowledge frontier is rapidly changing is related to an earlier literature showing that, in periods of rapid technological change, intergenerational mobility increases and the relative importance of the transmission of parental-specific human capital declines (Galor & Tsiddon, 1997). This idea is also present in studies of the decline of the family firms over the course of development (Carillo et al., 2019). Finally, we also provide new insights on the divergence of Catholic and Protestant universities beyond traditional explanations centred on religious values (Merton, 1938) or the Counter-Reformation (Landes, 1998; Blasutto & De la Croix, 2023; Dewitte et al., 2022). More generally, our results relate to a large literature showing that distortions in high-talent markets can drastically affect the production of ideas (Bell et al., 2018). Examples of such distortions include family-successions of CEOs (Pérez-González, 2006; Bennedsen et al., 2007).

## Institutional background and data

### Recruitment

Although norms varied across universities and academies, the recruitment process shared some general characteristics. The recruitment of university professors typically involved the faculty, who proposed to appoint a candidate to a chair, and an external authority (e.g., Monarch, Church, Municipality, Corporation), who approved it. Most chairs were filled by public competition, but appointments were sometimes transferred to a representative of the authorities (Rashdall 1895: vol 2, p. 192). For example, the University of Copenhagen initially appointed its professors. Following the introduction of Absolute Monarchy in 1660, these appointments had to be approved by the King. Both steps of the recruitment process were subject to nepotism. Slottved and Tamm (2009, 42–43) argues that Thomas Bartholin (1616–80) used his social connections both at the University of Copenhagen and at the court to promote his relatives’ careers. On the one hand, his permanent position as Dean of the Medical Faculty gave him influence over matters of importance at the University, particularly over appointments. On the other hand, Bartholin ingratiated himself with the King’s chancellor, who also served as Chancellor of the University. Other well-documented examples of nepotism are the Géraud de Vaxis and Lefranc families, who secured jobs at the University of Cahors for several generations (Ferte, 2000). Interestingly, Ferté wonders why it was so important for them to secure those jobs given the low salary paid by the University. The reasons were prestige and notability, the same factors pushing people to publish (Mokyr, 2016).

In academies, new members were elected by co-option—that is, they were elected at the discretion of existing members. In general, a member (or a group of members) sponsored an external candidate. All academy members then voted whether to accept this candidate (Foster & Rucker, 1897). The available election certificates of Royal Society fellows shows that fathers never sponsored their sons there. This suggests that, if there was nepotism, it was the result of fathers influencing the vote of their fellows. In some academies, the candidates had to submit a written work for evaluation (Galand, 2009). As in universities, the nomination of new academy members was sometimes subject to the approval of external authorities. For example, in the French and Spanish Academies, the votes for new members had to be approved by the King.

Besides chaired professors and academy members, our database contains a myriad of other scholarly positions. These include university regents in France, docents in Germany, or fellows in England, and various positions in academies, e.g., corresponding member, honorary member, free member. These positions were typically used as a stepping stone to a university chair or an academy membership. The recruitment rules for these intermediate positions varied across institutions, but generally they involved insiders, that is, the faculty or other academy members.

It is important to note that the decision to apply for an academic position was multifaceted and not a simple binary choice, as many scholars simultaneously held positions at universities and engaged in other occupations. In other words, taking an academic job did not imply abandoning other high-skilled jobs. For example, Polycarp Leyser (1586–1633), son of Polycarp Leyser (1552–1610), held a chair of theology at the university of Leipzig from 1613 (Junius Institute (2013)) but was also a canon at Wurzen, superintendent of Leipzig (since 1828), and provost in Zeitz (Allgemeinen Deutschen Biographie). Argentine Arsendi (1320–1388), son of Raniero Arsendi (1290–1358), was a professor of civil law at the University of Padova since 1351. This did not prevent him from acting as political negotiator and diplomat on behalf of the Lord of Padova, Francesco I da Carrara. According to Istituto dell’Enciclopedia Italiana (1961), he deserves to be remembered above all for the diplomatic activity in the service of the Carraresi. A position at university often implied teaching one or two courses, leaving ample time to conduct other activities at the same time. This is even more true for academies which only held meetings from time to time.

Although the virtues of meritocracy over nepotism are known since antiquity (Ciulla, 2005),^11^ academia became more open and meritocratic only from the 16th century onwards (Wooldridge, 2021). The historical narrative suggests that this process was associated with the foundation of new institutions—such as the university of Göttingen (1737) and the various academies of sciences (see, e.g., Mokyr 2016), whereas old institutions—such as most medieval universities—remained attached to old paradigms. In Sect. 5.3, we provide the first systematic evidence supporting these claims.

More generally, the early prevalence of nepotism and the later increase in meritocracy in academia was concomitant to broader trends in society. Early on, nepotism in society was recognized as problematic and discussed by several authors. For example, Simon Stevin (1548–1620), professor at the university of Leiden, wrote on how to change and improve the young Dutch state. Stevin “paid a lot of attention to, and expressed great concern about corruption and nepotism, two problems which current researchers have not recognized as prominent or topical in the early seventeenth-century Dutch Republic” (van Aelst, 2020). Around the time when new universities and academies were established during the Scientific Revolution, other merit-based institutions began to appear, such as a civil service for the administration of India by the British Empire in the 17C (Kazin, Edwards, and Rothman 2010: p. 142).

### Data

This section describes the dataset that we constructed for this paper and discusses the coverage and accuracy of the data. Appendix A lists the most important sources used and provides additional summary statistics and examples.

*Father–son pairs in academia* We build a new dataset of fathers and sons in the same university or scientific academy in Europe from 1088 to 1800. To construct this dataset, we use 343 secondary sources together with encyclopedias and biographical dictionaries. First, we assemble a list of the scholars in each university and academy. To do so, we use historical catalogues of the scholars in an institution, compendia of professors, books with biographies and bibliographies of a university’s scholars, and books on the history of a university or academy. Examples of these secondary sources are Mazzetti (1847)’s comprehensive list of University of Bologna professors since 1088, online catalogues of all members of the Royal Society and the Leopoldina academies, and Conrad (1960)’s list of all University of Tübingen chair holders. For universities and academies without a members’ catalogue or a book on their history, we assemble a list of their scholars by combining multiple secondary sources listed in Appendix A. For example, for the University of Avignon, a sample of professors was drawn from Laval (1889) for the medical faculty, Fournier (1892) and de Teule (1887) for lawyers, and Duhamel (1895) for rectors.^12^ The resulting list of scholars can be accessed at https://shiny-lidam.sipr.ucl.ac.be/scholars/. Not surprisingly, the gender distribution leans heavily towards men, with only 0.2% female scholars (De la Croix and Vitale, 2023).

Second, we identify all father–son pairs in the list of scholars in each university and academy. The secondary sources described above often mention if a scholar was related to another scholar. In addition, we use biographical dictionaries, encyclopedias about universities, and encyclopedias on the regions where universities were located to identify all father–son links. Following on the examples described above, we use the Treccani encyclopedia, the Dictionary of National Biography, the *Allgemeine Deutsche Biographie*, and the biographical dictionary of the Department of Vaucluse (Barjavel, 1841) to code fathers and sons in, respectively, the University of Bologna, the Royal Society, the Leopoldina and the University of Tübingen, and the University of Avignon.

In addition, we use these sources to record each scholar’s birth, nomination, and death year and their field of study. We consider the following broad fields: lawyers, physicians, theologians, scientists, and arts and humanities’ scholars. These fields correspond to the three higher faculties of early universities plus the arts faculty, where scientists gained importance over time. We also use Frijhoff (1996), British Library Board (2017) and McClellan (1985) to record the foundation date of each university and academy and its religious affiliation after the Protestant reformation. Finally, we follow the procedure described above to collect data on 507 father–sons pairs who were appointed to a different university or academy—whom we use to perform a validation exercise in Sect. 6.3.

Next, we discuss the limitations of our dataset and the accuracy of the father–son pairs. The main limitation is that we only observe the children of scholars who become scholars themselves. Hence, our estimates for the intergenerational elasticity of human capital are not population estimates, but reflect the transmission of upper-tail human capital in academia in 1088–1800. The biggest threat to estimate this elasticity, as well as nepotism, is if the sources used selectively report father–son links. One possibility is that links appear more frequently when fathers are famous: a father of no great account may be more likely to fall by the wayside than an underachieving son of a famous scholar. As a result, the data would be effectively selected on an outcome: father’s publications. This sampling bias could explain the father–son distributional differences and attenuate intergenerational correlations in outcomes (Solon, 1989), the two sets of moments used in our estimation. To assess the sensitivity of our analysis to this sampling bias, we classify the 343 sources used into three levels of completeness: First, sources with *complete coverage* cover all scholars in a university or academy, e.g., a catalogue of university professors. Under complete coverage we can fully rule out the possibility of sampling bias. Second, sources with *broad coverage* cover a large sample of scholars in an institution where a members’ catalogue does not exist, e.g., a book on the history of the university. Under broad coverage, sampling bias is less likely, although we cannot fully rule it out. Third, sources with *partial coverage* describe the case where the sample of scholars in an institution was inferred by secondary sources from other institutions and/or by general thematic biographies. Under partial coverage, there is risk of sampling bias.

Table 1 shows the percentage of observations in our data under each coverage category. Around two thirds of our father–son pairs are from sources with complete coverage, 95.9% from sources with complete and broad coverage, and only 4.1% from sources with partial coverage. The data coverage does not change significantly over time. Before the Scientific Revolution (1088–1543), 62.2% of father–son pairs are from sources complete coverage. The corresponding figure for the Enlightenment (1688–1800) is 72.7%. Overall, the percentage of father–son pairs are identified from sources with complete or broad coverage ranges little across historical periods, from 93.3% in the second stage of the Scientific Revolution (1633–1687) to 97.1% in the pre-1543 period. In addition, the percentage of father–son pairs from complete and broad sources does not differ substantially by country, by century, by the religion of the university, by field of study, nor by the prestige of the university (see Appendix Fig. A.1).^13^ Altogether, this suggests that the sampling bias described above is not prevalent in our data. That said, we examine the sensitivity of our main results to this sampling bias by presenting separate estimates using data only from sources with complete coverage.

**Table 1:** Data coverage (in %)

	Complete	Broad	Complete and Broad	Partial	N
All	64.1	31.8	95.9	4.1	1837
Pre-Scientific Revolution, 1088–1543	62.2	34.9	97.1	2.9	347
Scientific Revolution (I), 1543–1632	55.4	41.3	96.7	3.3	383
Scientific Revolution (II), 1633–1687	60.1	33.2	93.3	6.7	434
Enlightenment, 1688–1800	72.7	24.1	96.8	3.2	673

Our final dataset contains 1621 fathers and 1837 sons in the same university or academy. We also observe 176 families with three or more generations of scholars.^14^ Sons who worked in the same institution as their fathers represent around 5% of the known faculty—although there is heterogeneity across time and institutions.^15^ This percentage illustrates only the tip of the iceberg of favouritism in academia, as we only observe father–son connections but not nepotism towards other relatives (e.g., nephews, cousins) nor favouritism towards friends and acquaintances. Our dataset covers 116 universities and 63 scientific academies. These universities had, on average, 410 scholars and 11.8 academic dynasties; i.e, families in which more than one generation was employed in that university. We find the birth year for 76.6% of scholars, the death year for 86.4%, the nomination date for 91.1%, and the field of study for all.

**Fig. 2:**
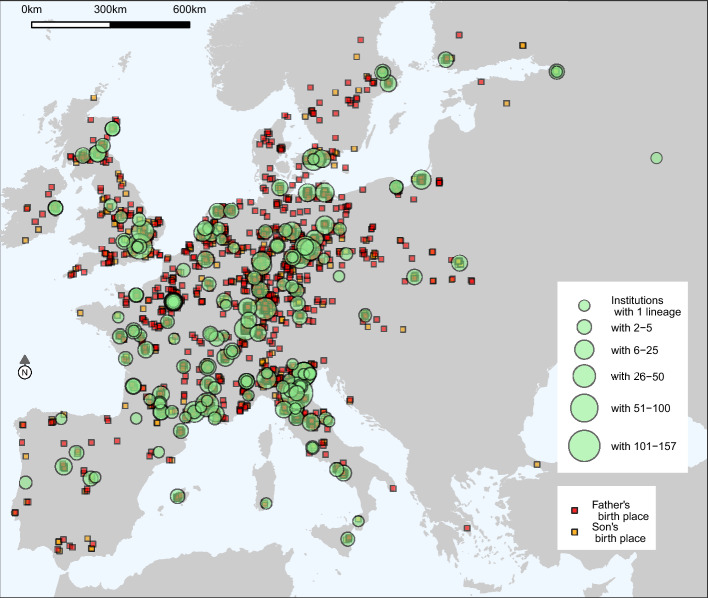
Geographical distribution of father–son pairs

Figure 2 shows the geographical distribution of our data. The recorded universities and academies (green circles) cover most of Europe. We observe 28 universities and 6 academies in the Holy Roman Empire, 32 universities and 24 academies in France, 7 universities and 8 academies in England, Scotland, and Ireland, and 7 universities and one academy in the Netherlands. In southern Europe, we cover 20 universities and 16 academies in Italy and 6 universities and one academy in Spain. We have several universities in eastern (e.g., Moscow, St. Petersburg) and northern Europe (e.g., Copenhagen, Lund, Turku, Uppsala). The map also displays birth places (orange for fathers, red for sons). Most scholars originate from north-west and central Europe and from Italy.

**Fig. 3:**
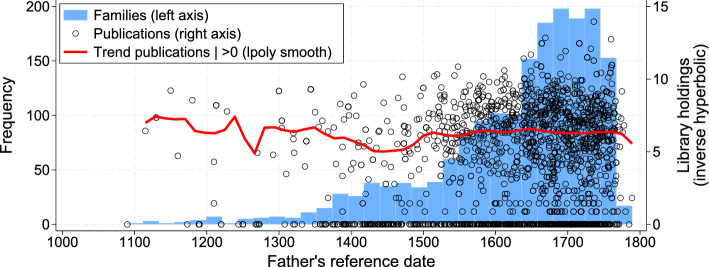
Number of scholar families and father’s publications. *Notes*: Reference date based on birth year, nomination year, or approximative activity year

The dataset spans seven centuries from 1088—the year of the foundation of the University of Bologna—to 1800. Half of the universities in our dataset were established before 1500, e.g., the University of Paris (officially established in 1200, but starting before), Oxford (1200), Cambridge (1209), Salamanca (1218), Prague (1348). That said, most scholars under analysis are from after the 1400s. Figure 3 plots the number of father–son pairs over time. Before 1400, we observe 104 families. This number increases after 1400 and peaks during the Scientific Revolution.

*Publications data* We measure the scientific output of each of the 1621 fathers and 1837 sons in our dataset. To do so, we use WorldCat—an online catalogue of the library holdings of more than 10,000 modern libraries worldwide. Specifically, we link each scholar to his entry in the WorldCat service and record his publications.

We compile three measures of a scholar’s scientific output: the total number of library holdings in modern libraries written by and about each scholar, the library holdings written by each scholar, and the number of unique published works by or about each scholar. Our preferred measure is the total number of library holdings by and about each scholar. This includes all imprints/editions/copies of books, volumes, issues, or documents which he wrote that are available in WorldCat libraries today. It also includes publications about his work written by a different author.^16^ Hence, our measure captures both the size and the relevance of a scholar’s production for today. In other words, library holdings by and abour a scholar is a measure akin to citations in modern academia, in the sense that it captures the quality of publications. In addition, while the number of unique published works may reflect nepotism or social connections in the publishing industry in the past, it is unlikely that the total number of library holdings in modern libraries today is affected by nepotism or social connections in the publishing industry centuries ago. Hence, we use library holdings by and about each scholar as our baseline measure of publications, and examine the robustness of our results to using unique publications and to excluding publications on a scholar’s work written by others in Sect. 7. Unless we indicate otherwise, throughout the paper we define publications as the inverse hyperbolic sine of the number of library holdings. We do so to estimate elasticities on a variable with a skewed distribution and some zeros (Bellemare & Wichman, 2020).^17^

We illustrate how this data was collected with an example: Honoré Bicais and his son Michel (see Appendix Fig. A.4). Both are listed as University of Aix professors in Belin ’s (1905) *Histoire de l’Ancienne Universite de Provence* (De la Croix and Fabre, 2019). Honoré’s biography states that Michel succeeded him “in his chair and in his reputation.”^18^ To measure their publications, we link Honoré and Michel Bicais to their WorldCat entries. WorldCat considers different spellings of the family name (Bicais, Bicaise, Bicays, and the latinized Bicaisius and Bicaissius), which ensures that the matching of authors to publications is accurate. Honoré Bicais was a prolific scholar: there are 315 library holdings of books written by him. In contrast, modern libraries only hold 16 copies of the work of his son Michel. While Michel succeeded his father in his chair, it is less clear that he did so too in his academic reputation.

Our data on scholar’s publications is comprehensive and accurate. Chaney (2020) conducted a validation exercise showing that WorldCat accurately approximates the population of known European authors. Specifically, he compared the Universal Short Title Catalogue Andrews to the references in the Virtual International Authority File (VIAF), on which WorldCat is based. Chaney successfully located 81% of USTC authors in the VIAF. In our setting, we do not find WorldCat entries for 38% of sons and for 29% of fathers. Given WorldCat’s coverage, these scholars likely never published. Nevertheless, we account for the possible loss of their publications in two ways. First, our estimation uses separate empirical moments for the intensive margin (i.e., publications conditional on being listed in WorldCat) and the extensive margin (i.e., being listed in WorldCat). Second, our model accounts for separate measurement error in the intensive and extensive margins. That is, it accounts for the possible loss of a scholar’s publications. This allows us to disentangle changes in the extensive margin of publications from other dynamics (e.g., changes in nepotism).

Our final dataset comprises 487,041 unique works and 4,106,901 library holdings. Figure 3 illustrates their time trends. It shows the inverse hyperbolic sine of the library holdings by and about fathers. The figure suggests that there is no upward trend in the number of publications, conditional on being positive. Appendix G validates this finding using the De la Croix (2021) data for all known pre-industrial scholars (not only fathers and sons). That said, we find evidence of a structural break around 1450 on the probability of being listed in WorldCat (see Appendix Fig. G.2). The historical evidence suggests that this break is related to the changes brought about by the printing press, rather than with a change in the human capital distribution or in nepotism.^19^ In other words, it affects fathers and sons similarly. Our model and estimation account for changes over time in the extensive margin of publications (Sect. 5.3), and Sect. 7 and Appendix G discuss the sensitivity of our results to this structural break.

## Two facts about fathers and sons in academia

Anecdotal evidence suggests that both nepotism and inherited human capital mattered for the careers of pre-industrial scholars. For example, Jean Bauhin (1541–1613), professor in Basel, has a remarkable publication record: there are 1471 library holdings of his work in modern libraries. Michaud’s *Biographie Universelle* emphasizes how Bauhin’s knowledge was inherited from his father, also a professor in Basel:

Jean Bauhin (1541–1613) learned very early the ancient languages and humanities. His father, Jean Bauhin, was his first master in the study of medicine and of all the underlying sciences.

This contrasts with the case of the Benavente family at the University of Salamanca. Juan Alfonso Benavente has 108 publications available in WorldCat libraries today. According to the *Diccionario Biográfico Español*, he used his power and influence to pass down his chair to his son Diego Alfonso:

After sixty years of teaching canon law in Salamanca, Juan Alfonso Benavente (–1478) retired in 1463. He retained his chair and his lectures were taught by substitutes, including his son Diego Alfonso Benavente (c. 1430–1512). Finally, on 1477, Benavente resigned his chair on the enforceable condition that his son was appointed to it.

Diego Alfonso Benavente proved less productive than his father. He only published a compendium of his father’s work.

Table 2 documents two stylized facts for fathers and sons in premodern academia: a strong correlation in publications across generations and large differences in the marginal publications distribution of the set of fathers and sons. These two facts reflect the patterns outlined by the examples above: on the one hand, there was a strong transmission of underlying human capital endowments from fathers to sons in academia, which was later reflected in a strong correlation in their publication record. On the other hand, nepotism was present among pre-industrial scholars, generating father–son distributional differences over and above the rate of mean reversion implied by the intergenerational correlations in publications.

**Table 2:** Moments used in the estimation

		Value	s.e.	N
*A. Intergenerational correlations*				
Father–son, intensive margin	$\rho (y_{t}, y_{t+1}\mid _{y_{t}, y_{t+1}>0})$	0.375	0.03	982
Father–son with zero publications	Pr$(y_t{=}0 \wedge y_{t+1}{=}0)$	0.211	0.01	1837
Grandfather-grandson, intensive margin	$\rho (y_{t}, y_{t+2}\mid _{y_{t}, y_{t+2}>0})$	0.234	0.17	87
*B. Father–son distributional differences*				
Fathers with zero publications	Pr$(y_{t}{=}0)$	0.288	0.01	1621
Sons with zero publications	Pr$(y_{t+1}{=}0)$	0.384	0.01	1837
Fathers median	Q50($y_{t}$)	5.075	0.14	1621
Sons median	Q50($y_{t+1}$)	3.402	0.25	1837
Fathers 75th percentile	Q75($y_{t}$)	7.370	0.08	1621
Sons 75th percentile	Q75($y_{t+1}$)	6.413	0.09	1837
Fathers 95th percentile	Q95($y_{t}$)	9.425	0.12	1621
Sons 95th percentile	Q95($y_{t+1}$)	8.537	0.07	1837
Fathers mean	E($y_{t}$)	4.456	0.09	1621
Sons mean	E($y_{t+1}$)	3.477	0.08	1837

*Notes:* *y*: publications (inverse hyperbolic of library holdings by or about each scholar)

**Fig. 4:**
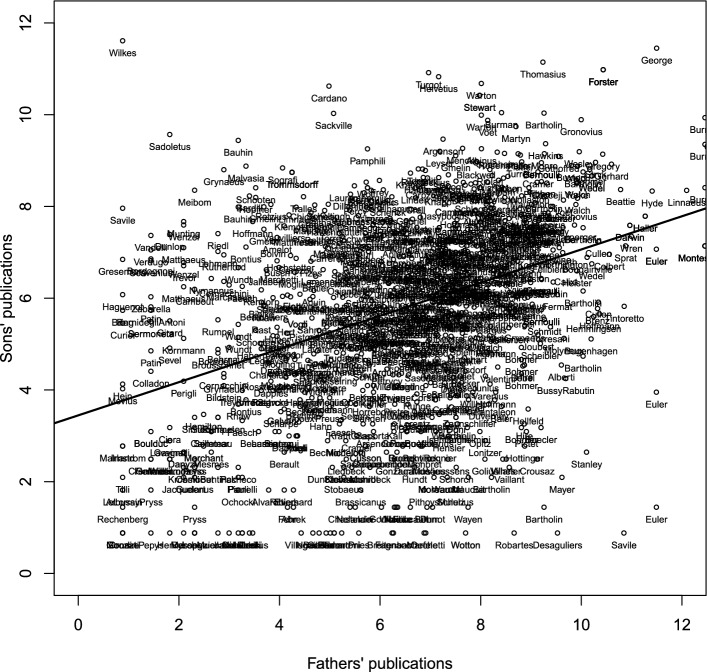
Father–son correlation in publications. *Notes*: The sample are 982 father–son dyads in academia where both have at least one publication

**Fact 1: High correlation of publications across generations.** Table 2, Panel A presents father–son correlations in publications, measured as the inverse hyperbolic sine of the number of library holdings. We distinguish correlations conditional on both father and son having at least one observed publication (intensive margin) from the proportion of pairs where father and son have zero publications (extensive margin). The correlation on the intensive margin is 0.375 (see also Fig. 4). This implies that an increase of 1% in a father’s publications is associated with an increase of 0.375 percent in his son’s publications.^20^ As for the extensive margin, in 21% of families both father and son have zero publications. In sum, publication records were persistent across two generations. This suggests that endowments determining publications, e.g., human capital, were partly transmitted from parents to children. In addition, lineages with three generations of scholars display high correlations in publications on the intensive margin. The correlation between grandfathers and grandsons is 0.23. This number is larger than predicted by the iteration of the two-generation correlation, i.e., 0.375$^2=0.141$. In other words, the advantages of ancestors vanished at a slower rate than suggested by father–son correlations.

We perform two exercises to validate the accuracy of Fact 1. First, we compare the moments in Table 2 to moments obtained using subsamples of fathers and sons from data sources with better coverage (see Appendix Table F.1). The father–son correlation on the intensive margin is 0.36 (s.e. 0.04) when we only use sources with complete coverage, and 0.38 (s.e. 0.03) when we use sources with complete and broad coverage. These moments are not statistically different than our baseline moment, 0.375 (s.e. 0.03). Similarly, the proportion of father–son pairs with zero publications and the grandfather-grandson correlation are not sensitive to restricting the data to sources with better coverage. Altogether, this strongly suggests that the high correlation of publications across generations (Fact 1) is accurate and not a by-product selective reporting of father–son links in sources that do not cover the universe of scholars in an institution.

Second, we show that Fact 1 is not driven by changes in the father’s and son’s marginal distributions which could reflect, e.g., trends in the quantity of publications over time. Note that Fact 1 is based on correlations in publications instead of on regression elasticities akin to an intergenerational elasticity (IGE). We prefer correlations because they are scale-invariant measures (Chetty et al., 2014, p.1561). Instead, regression elasticities conflate the join distribution of father and son ranks (the copula) with changes in the father’s and son’s marginal distributions. We confirm that our analysis is not driven by changes in the marginal distributions by showing that Fact 1 is robust to comparing father–son ranks in publications. We follow Chetty et al. (2014) and rank sons based on their publications relative to other sons in the same 50-year birth cohort. We rank fathers based on their publications relative to other fathers with sons in these 50-year birth cohorts. The father–son correlation in percentile-ranks is 0.39 in the intensive margin, almost identical to the coefficient reported in Table 2.

**Fact 2: The publication’s distribution of fathers first order stochastically dominates (FOSD) that of sons.** Table 2, Panel B presents 10 moments describing the marginal distribution of publications for fathers and for sons. On the bottom of the distribution, 38% of sons had zero publications. The corresponding figure for fathers is 29%. The average father had more than two times more publications than the average son (43 vs. 16, in levels). Fathers also have two times more publications than sons in the 75th and the 95th percentile of the distribution. The difference is larger at the median: there, fathers published five times more than sons (80 vs. 15, in levels).^21^

**Fig. 5:**
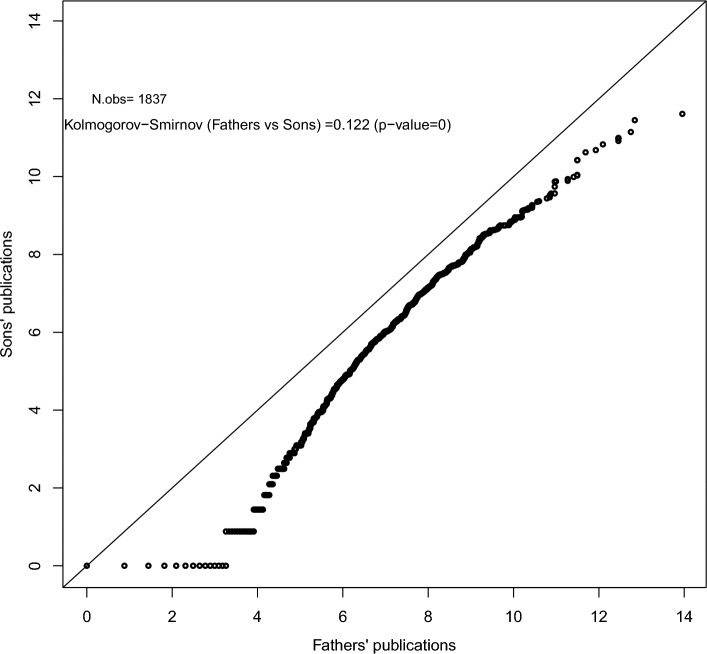
Quantile-quantile plot

To illustrate these differences, Fig. 5 presents a QQ-plot; a plot of the quantiles of the fathers’ distribution against the quantiles of the sons’ distribution. If the two distributions were similar, the points would lie on the 45 degree line. Instead, in all quantiles fathers have larger publication records. That is, the father’s publication distribution FOSD that of their sons. Importantly, the distributional differences are larger at the bottom of the distribution.

As before, we validate Fact 2 by comparing our baseline moments to moments obtained using data sources with better coverage that cover the universe of professors in an institution. Appendix Table F.1 shows that the fathers’ publication distribution FOSD that of sons also when we only use father-son pairs from sources with complete coverage, and when we only use sources with complete and broad coverage. This shows that Fact 2 also holds in data sources that do not selectively report father–son links when, e.g., fathers are famous scholars. Hence, it is highly unlikely that the observed wedge between the publications of fathers vs. sons is driven by sampling bias in our sources.

The large distributional differences suggest that fathers had higher human capital endowments than sons, which transformed into a better publication record. Partly, this difference in human capital endowments is explained by reversion to the mean. We are looking at a sample at the top of the human capital distribution, and hence, if there is reversion to the mean, sons should be worse than fathers. That said, the rate of mean reversion needed to explain away the observed distributional differences is implausibly high, especially in light of the high correlation in publications across generations (Fact 1). In other words, Facts 1 and 2 are hard to reconcile with standard mean-reversion models based solely on human capital transfers (Becker and Tomes 1979, 1986).

Instead, two pieces of evidence suggest that the bulk of these distributional differences reflect nepotism. That is, that fathers used their influence in the profession to allocate jobs to their sons ahead of outsiders, even when sons had low human capital endowments. The first is that sons of scholars had a worse publication record not only than their fathers, but also than outsiders whose parents were not academics (see Fig. 1 and Sect. 6.2). The second is that distributional differences are larger at the bottom of the distribution. That is, close to the human-capital threshold that determines whether an individual is selected to become a scholar or not, and where nepotism could be binding. Altogether, this kind of nepotic hiring can generate father–son distributional differences, especially at the bottom of the distribution, even when human capital slowly reverts to the mean. In our estimation, we use these excess distributional differences, net of reversion to the mean, to identify nepotism.

## Model of human capital transmission with nepotism

To account for these patterns, our model incorporates nepotism into a standard first-order Markov process of human-capital endowments’ transmission. We consider a population of potential scholars who are heterogeneous in their human capital. The human capital of each potential scholar depends on a human capital endowment inherited from his father and on random ability shocks.^22^ Potential scholars with high human capital are selected to be a scholar. We introduce the possibility of nepotism by allowing this selection criterion to be different for sons of scholars. For selected scholars, the unobserved human capital endowment translates into an observed outcome, publications, with noise.

Each potential scholar is indexed by $i \in \mathbb {I}$, their family, and by ${\textbf {t}}= \{t, t+1,...\}$, their generation. A potential scholar in generation *t* of family *i* is endowed with an unobserved human capital $h_{i,t}$. This is distributed according to a normal distribution with mean $\mu _h$ and standard deviation $\sigma _h$:

1 $$\begin{aligned} h_{i,t} \sim N(\mu _h,\sigma _h^2) \ . \end{aligned}$$

The offspring of this generation, indexed $t+1$, partly inherit the unobserved human capital endowment under a first-order Markov process:

2 $$\begin{aligned} h_{i,t+1} = \beta h_{i,t} + u_{i,t+1} \ , \end{aligned}$$

where $\beta$ is the intergenerational human capital elasticity^23^ and $u_{i,t+1}$ is an i.i.d. ability shock affecting generation $t+1$, which has a normal distribution, $N(\mu _u,\sigma _u^2)$.

At each generation, only a selected group of potential scholars with human capital above $\tau \in \mathbb {R}$ become scholars. We allow sons of scholars to become scholars if their human capital is above $\tau -\nu$. If $\nu > 0$, the selection process into becoming a scholar is subject to nepotism, in the sense that sons of scholars are selected into academia under a softer human-capital criterium than their fathers. Formally, the set $\mathbb {P}$ denotes the set of father–son pairs:

3 $$\begin{aligned} \mathbb {P}=\{i \mid h_{i,t}>\tau , h_{i,t+1}>\tau -\nu \} \subset \mathbb {I} \ . \end{aligned}$$

For selected scholars, human capital is transformed into an observable outcome *y* with measurement error. In our case, scholars use their (unobservable) human capital to produce knowledge in the form of (observable) publications. We depart from previous literature and consider two sources of measurement error: one on the intensive margin and one on the extensive margin. On the intensive margin, we consider idiosyncrasies in the publication process, shocks to an individual’s health, luck, etc. that can affect a scholar’s number of publications independently of his human capital. On the extensive margin, our empirical application needs to account for the possibility that some publications might be lost or are not held in modern libraries. That is, that we are more likely to observe the publications of a scholar with a larger record of publications. Formally, the publications for fathers, $y_{i,t}$, and sons, $y_{i,t+1}$, in the set of observed scholar families $\mathbb {P}$ are:

4 $$\begin{aligned} y_{i,t}= & {} h_{i,t} + \varepsilon _{i,t} \;\;\;\;\; \text{ if } \;\;\; h_{i,t} + \varepsilon _{i,t}>\kappa ,\;\;\;\;\; y_{i,t} = 0 \text{ otherwise } \end{aligned}$$

5 $$\begin{aligned} y_{i,t+1}= & {} h_{i,t+1} + \epsilon \;\; \text{ if } \;\; h_{i,t+1} + \epsilon _{i,t+1}>\kappa ,\;\;\; y_{i,t+1} = 0 \text{ otherwise } \end{aligned}$$

where $\varepsilon _{i,t}$, $\epsilon _{i,t+1}\sim N(0,\sigma _e^2)$ are mean-preserving shocks affecting how human capital transforms into publications; and $\kappa$ is the minimum number over which we observe a scholar’s publications. The former captures measurement error on the intensive margin, the latter on the extensive margin.

We assume that the human capital of *potential scholars* in consecutive generations *t* and $t+1$ (that is, the human capital of fathers, $h_{i,t}$, and of sons, $h_{i,t+1}$ in $\mathbb {I}$) is drawn from the same distribution. This stationarity assumption allows us to put structure on how much of the distributional differences between *observed* fathers and sons in $\mathbb {P}$ can be explained by pure reversion to the mean—that is, independently of nepotism. Formally, $h_{i,t} \sim N(\mu _h,\sigma _h^2)$ and $h_{i,t+1} = \beta h_{i,t} + u_{i,t+1}$ imply that $h_{i,t+1} \sim N(\beta \mu _h+\mu _u,\beta ^2\sigma _h^2+\sigma _u^2)$. Imposing stationarity leads to the following parameter restrictions:

6 $$\begin{aligned} \mu _u= & {} (1-\beta ) \mu _h \end{aligned}$$

7 $$\begin{aligned} \sigma _u ^2= & {} (1-\beta ^2) \sigma _h^2 \ . \end{aligned}$$

In Sect. 5.3, we relax this assumption. We assume that the human capital of a father and a son who are active in a given time period is drawn from the same distribution, but we allow the human capital distribution to change across periods.

We can now characterize our two main parameters of interest: the intergenerational elasticity of human capital and the magnitude of nepotism. First, the intergenerational elasticity of human capital, $\beta$, is given by Equation (2) and the stationarity conditions above, which imply:

8 $$\begin{aligned} h_{i,t+1} = \beta h_{i,t} + (1-\beta ) \mu _h + \omega _{i,t+1} \ , \end{aligned}$$

where $\omega _{i,t+1}$ is a shock distributed according to $N(0,(1-\beta ^2) \sigma _h^2)$. Equation (8) suggests that a son inherits a fraction $\beta$ of his father’s human capital, draws a fraction $(1-\beta )$ from the population mean, and is subject to a mean-preserving shock $\omega$. Hence, $\beta$ determines the speed at which inherited human capital advantages revert to the mean. For low values of $\beta$, the rate of mean reversion will be fast, and hence, we will observe large father–son distributional differences independently of nepotism. For high values of $\beta$, the rate of mean reversion will be slow, and hence, father–son distributional differences will reflect a different human-capital selection criterium for fathers and sons into academia, that is, nepotism.

Second, we define the magnitude of nepotism, $\gamma$, as the share of sons in academia who would not have become scholars under the same selection criterium as their fathers. This share is determined by parameters $\nu$ and $\tau$ in Eq. (3), but also by the distribution of human capital among all potential scholars and, as explained above, by the rate of mean reversion.^24^ Formally,

9 $$\begin{aligned} \gamma = F_h(\tau \ | \ h_{i,t+1}\ge \tau -\nu ) \ , \end{aligned}$$

where $F_h(x)$ is the (stationary) cumulative distribution of human capital with mean $\mu _h$ and variance $\sigma _h^2$, and $F_h(x \ | \ h_{i,t+1}\ge \tau -\nu ) = Prob \left( h_{i,t+1}\le x \ | \ h_{i,t+1}\ge \tau -\nu \right)$ is the corresponding truncated cumulative distribution of sons’ human capital in the set of observed scholars $\mathbb {P}$.

Note that $\gamma$ is a conservative estimate of nepotism. First, according to Eqs. (4) and (5), the human capital endowment transmitted across generations, *h*, includes skills but also any other inputs that facilitate sons’ publications, e.g., inherited social connections to publishers. In other words, $\gamma$ is restricted to nepotic selection into academia net of any unobserved endowment that positively affects a scholar’s research output—which here is captured by $\beta$. Second, $\gamma$ considers sons hired in academia thanks to their parents’ connections (hiring stage), but is conditional on the sons’ choice of an academic career (candidate stage). Because we do not observe the universe of potential scholars, we cannot model the ex-ante problem of choosing between academia and other activities. Hence, our $\gamma$ estimate abstracts from nepotism at the candidate stage. Although these two factors make nepotism a lower-bound estimate, the bias is likely small. This is because we use the number of library holdings in modern libraries instead of unique published works. Our measure captures research quality and relevance for today, and hence, is less sensitive to inherited connections to publishers omitted by $\gamma$. In addition, as explained in Sect. 2.1, academic jobs did not preclude scholars from taking up other high-skilled jobs. In this sense, the candidate-stage problem was less binding in our context, and hence, $\gamma$ should be largely robust to changes in scholar’s outside options over time or by field.

Estimating Eqs. (8) and (9) is challenging for two reasons: First, human capital endowments *h* are often unobserved and only reflected in observed outcomes, *y*, with measurement error (see Eqs. (4) and (5)). Second, note that Eq. (8) describes the mean-reversion process among *potential* scholars, while only those who actually become scholars are observed (see Eq. (3)). Hence, estimates of $\beta$ also need to address issues related to selection and, in this setting, of nepotism. Next, we explain how we address measurement error and selection in the form of nepotism to estimate Eqs. (8) and (9).

## Identification of parameters and main results

We identify the deep parameters of our model of human capital transmission with nepotism using the two Facts described in Sect. 3. Specifically, we identify six parameters by minimizing the distance between 13 simulated and empirical moments in Table 2. This minimum distance procedure is used to estimate the intergenerational elasticity of human capital ($\beta$), the magnitude of nepotism ($\gamma$), the noise with which unobserved human capital is transformed into observed publications ($\sigma _e$ and $\kappa$), and the shape of the human capital distribution ($\mu _h$ and $\sigma _h$). Finally, the parameters $\mu _u$ and $\sigma _u$ are pinned down from the stationarity conditions (6) and (7). We assume $\tau =0$ without loss of generality and recover $\nu$ from Eq. (9).

### Minimum distance estimation

The six parameters described above are identified by minimizing the distance between 13 simulated and empirical moments listed in Table 2: First, we consider the father–son correlation in publications conditional on both having at least one publication (intensive margin) and the proportion of father–son pairs with zero publications (extensive margin). Such father–son correlations in observed outcomes—especially, on the intensive margin—are widely used in the literature to estimate intergenerational elasticities and the rate of mean reversion, $\beta$ (see Black and Devereux 2011). When observed, we also consider the grandfather-grandson correlation in the intensive margin. As proposed by Lindahl et al. (2015) and Braun and Stuhler (2018), we use these multi-generation correlations to address measurement error in the extent to which observed publications reflect unobserved human capital ($\sigma _e$ and $\kappa$). Specifically, multi-generation correlations address measurement error under the assumption that this error is stable across generations.^25^ We provide evidence supporting this assumption in Appendix Fig. G.1.^26^ Second, we consider ten moments describing the marginal distribution of publications for the set of fathers and sons: the mean, median, 75th and 95th percentiles, and the proportion of zeros in the publications’ distribution.^27^ Together with the previous moments, father–son distributional differences help us to jointly identify the rate of mean reversion, $\beta$, and the magnitude of nepotism, $\gamma$. To see this, note that a slow rate of mean reversion will generate large father–son correlations in outcomes and small father–son distributional differences. In contrast, a large magnitude of nepotism will generate large father–son distributional differences at the bottom of the distribution (i.e., closer to the selection thresholds) even when intergenerational correlations suggest a slow rate of mean reversion. This is because, under nepotism, the human capital of selected sons will be low relative to that of selected fathers even under a slow rate of mean reversion. In addition, the 10 distributional moments also identify the shape of the human capital distribution ($\mu _h$ and $\sigma _h^2$). Appendix C illustrates our identification strategy with simulations.

In sum, our method recovers the intergenerational human capital elasticity from the father–son correlations in publications (the copula); and nepotism from the excess differences between the marginal distributions of fathers and sons, net of the effect of mean reversion in human capital.

Formally, we minimize the following objective function:

10 $$\begin{aligned} \min _{p} V(p) = \sum _{j} \lambda _j \left( \frac{\hat{m}_j(p) - m_j}{\sigma _{m_j}}\right) ^2 \end{aligned}$$

where *j* indexes the 13 moments described above, $p'=[\beta \ \gamma \ \mu _h \ \sigma _h \ \sigma _e \ \kappa ]$ is the vector of model’s parameters, $m_j$ is *j*’s empirical moment, $\hat{m}_j(p)$ is *j*’s simulated moment, $\sigma _{m_j}$ is the standard deviation of empirical moment *j*, and $\lambda _j$ is the weight of moment *j*. We attach higher weights to three moments which are most useful for identification. The first two are the proportions of fathers and sons with zero publications, which capture distributional differences close to the selection thresholds. The third is the standard moment in the literature: the father–son correlation in the intensive margin. $\lambda _j$ is arbitrarily large for these three moments, and $\lambda _j=1$ otherwise.

This procedure belongs to the family of the Simulated Method of Moments (Gourieroux et al., 1993; Smith, 2008), a structural estimation technique used when theoretical moments cannot be computed explicitly and need to be simulated. To compute the simulated moments, we draw 50,000 hypothetical families consisting of three generations: father, son, and grandson. Each generation’s human capital and publications are calculated according to Eqs. (1), (2), (4), and (5). Our simulated moments are computed from a sample of families in which fathers and sons meet the criteria to become scholars (Eq. (3)). Our simulated grandfather-grandson correlation moments, in turn, are computed from a sample of these families in which scholar’s grandsons also meet the (nepotic) criteria to become scholars, i.e., $h_{t+2}>\tau -\nu$. We minimize the objective function *V*(*p*) using the Differential Evolution algorithm (Price et al., 2006) as implemented in R by Mullen et al. (2011). To compute standard errors, we draw 200 random samples from the original data with replacement, generate the 13 moments for each bootstrap sample, and estimate the model’s parameters.

### Aggregate results (1088–1800)

Table 3 presents the identified parameters for the entire period, 1088 to 1800. Our main estimates are the magnitude of nepotism, $\gamma$, and the intergenerational elasticity of human capital, $\beta$. We find evidence of nepotism for one in six scholar’s sons and an intergenerationa human capital elasticity of 0.63. Next, we discuss the identified parameters in detail.

*Nepotism* Our $\gamma$-estimate shows that nepotism was present in pre-industrial academia. Between 1088 and 1800, 18.7 percent of scholars’ sons were nepotic scholars. That is, they would not have become scholars under the same selection criteria as their fathers. The percentage of nepotic sons is precisely estimated and significantly different from zero. The magnitude of nepotism can also be illustrated by recovering the parameter $\nu$ from Eq. (9). Specifically, our estimates imply that the human capital required to become a scholar is lower for sons of scholars, $\tau {-}\nu =-8.99$, than it was for their fathers, $\tau =0$. Furthermore, our estimates for the mean, $\mu _h=1.87$, and the standard deviation, $\sigma _h=4.22$, of the human capital distribution imply that the son of a scholar could become a scholar even if his human capital was 2.6 standard deviations lower than the average potential scholar, and 2.1 standard deviations lower than the marginal outsider scholar (i.e., a scholar with human capital below but close to $\tau$).

As explained in Sect. 2, the biggest threat to estimate nepotism is if our data sources selectively report father–son links. Table 3 shows that our results are robust to using fathers and sons from sources with complete coverage—where we can fully rule out sampling bias—and sources with complete and broad coverage—where sampling bias is unlikely. The percentage of nepotism, $\gamma$, is 18.7% when we use all the data (column 1), 14.8% when we use data with complete coverage only (column 2), and 18.2% when we use data with complete and broad coverage (column 3). These three estimates are not statistically different from each other, strongly suggesting that our results are not driven by sampling bias in the recording of father–son links.^28^

Finally, we perform a counterfactual exercise to gauge how nepotism may have impacted scientific production in our sample of fathers and sons in academia. We simulate our model with the estimated parameters and replace nepotic scholars by outsiders. That is, we replace sons who would not have become scholars under the same criteria as outsides by outsiders drawn randomly from the human capital distribution in academia under no nepotism. This would increase by 23.15 percent the scientific output of the average scholar in our simulated economy.

**Table 3:** Identified parameters

		All	Complete coverage	Complete and broad coverage
		[1]	[2]	[3]
IGE human capital	$\beta$	0.63 (0.04)	0.59 (0.05)	0.64 (0.04)
Nepotism, %	$\gamma$	18.7 (1.74)	14.8 (2.14)	18.2 (1.77)
Mean human capital	$\mu _h$	1.87 (0.47)	2.84 (0.44)	1.96 (0.45)
SD human capital	$\sigma _h$	4.22 (0.20)	3.90 (0.22)	4.22 (0.21)
SD publications’ shock	$\sigma _e$	0.39 (0.15)	0.25 (0.13)	0.38 (0.17)
Threshold publications	$\kappa$	2.12 (0.14)	2.15 (0.19)	2.13 (0.14)

*Notes:* SE in parenthesis from 200 bootstrapped samples with replacement; degrees of overidentification: 6

*Human capital transmission* We estimate an intergenerational elasticity of human capital, $\beta$, of 0.63 among fathers and sons in academia. This implies that sons inherited 63% of their father’s human capital. As before, Columns 2 and 3 of Table 3 show that this estimate is very similar and not statistically different when we restrict the data to sources with complete coverage (0.59) and sources with complete and broad coverage (0.64).^29^

Our $\beta$-estimates are 11–17 percentage points larger than the parent–child elasticity in publications (0.46 with s.e. 0.02, see Table 7), a difference that is statistically significant.^30^ This supports Clark (2015)’s hypothesis that underlying endowments transmitted across generations (in this case, human capital) are more persistent than suggested by parent–child elasticities in outcomes. That said, our estimate is smaller than those based on average outcomes across rare surname groups, which cluster around 0.8–0.9. It is also at the bottom range of estimates using multi-generation correlations (e.g., Braun and Stuhler 2018) and the informational content of surnames (e.g., Güell, Rodríguez Mora, and Telmer 2015). This suggests that, in empirical applications where selection and nepotism are relevant, the multiple-generation methods in the literature can provide upward-biased $\beta$-estimates. In Sect. 6.4, we provide evidence for this by comparing our estimates to those obtained using alternative methods in the literature.

*Other parameters* Table 3 shows that the human capital distribution among potential scholars has a mean of $\mu _h=1.87$ and a standard deviation of $\sigma _h=4.22$. This implies that the average potential scholar can become a scholar, but not those one standard deviation lower than the mean—unless their father is a scholar. Using stationarity conditions (6) and (7) we pin down the mean and standard deviation of the random ability shock to human capital: $\mu _u=0.69$ and $\sigma _u=3.28$. We also find an imperfect relation between human capital and the production of ideas: The shock affecting how scholar’s human capital transforms into publications, $\epsilon$, has a standard deviation of $\sigma _e=0.39$. We also estimate a high $\kappa =2.12$, implying that a scholar who published 2 or 3 works may have no library holdings today. In other words, we do not impose a zero human capital (or nepotism) to scholars with no library holdings, but allows the possibility that his publications may be lost and are not held in modern libraries.

*Model fit* Here we compare the empirical moments to those simulated by our model; the details are available in Appendix D. We reproduce Fact 1, that is, the high correlation of publications between fathers and sons in the intensive (0.375 in the model vs. 0.375 in the data) and extensive margin (0.17 vs. 0.21). Our model also matches the grandfather-grandson correlation (0.19 vs. 0.23), as well as the empirical observation that this correlation is larger than predicted by iterating the two-generation correlation (0.375$^2=0.14$).

**Fig. 6:**
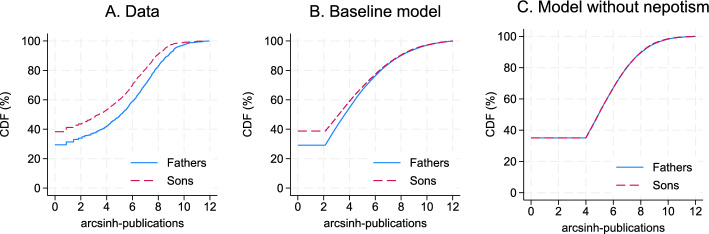
Empirical and simulated distribution of publications

We also reproduce Fact 2. This is illustrated in Fig. 6, which shows the empirical (Panel A) and simulated (Panel B) cumulative distribution function (CDF) of publications by the set of fathers and sons.^31^ We reproduce the observed distributional differences at the bottom of the distribution but also below the median. Our parsimonious model does not generate large differences at the very top, suggesting that these emerge independently of nepotism or human capital transfers. Importantly, Panel C shows that nepotism is crucial to reproduce the observed distributional differences. We consider an alternative model with $\gamma =0$, that is, where scholars’ sons and outsiders are selected into academia under the same criteria. In this alternative model, only mean reversion can generate distributional differences—since scholars are at the top of the human capital distribution, mean reversion will worsen the sons’ publications relative to that of their fathers. This effect should be larger for top vs. average scholars’ sons. The model without nepotism reproduces some small distributional differences in the 75th (6.58 for fathers vs. 6.57 for sons) and 95th percentile (8.98 vs. 8.89). That said, it fails to match Fact 2, as the CDFs are largely identical. In other words, the observed distributional differences are hard to reconcile with a model of *pure* mean reversion *à la* Becker and Tomes 1979, 1986, where persistence is explained with human capital transfers but not with inherited social connections and nepotism. Interestingly, the alternative model estimates a larger $\beta$ of 0.72, suggesting that ignoring the selection bias arising from nepotism can overstate intergenerational elasticities.

### Results over time

This section studies the evolution of nepotism and the intergenerational elasticity of human capital between 1088 and 1800. Were periods of rapid scientific advancement associated with a decline in nepotism, and hence, a better allocation of talent in academia? How important was the knowledge transmitted from parents to children during these periods? To answer these questions, we narrow our focus to the two proclaimed roots of all modern technological advances: the Scientific Revolution (Wootton, 2015) and the Enlightenment (Mokyr, 2010).

We divide our families of scholars into four periods based on the father’s reference date. We use standard dates marking the Scientific Revolution and the Enlightenment: **(i)** before 1543, when Copernicus published *De revolutionibus orbium coelestium*; **(ii)** 1543–1632, the beginning of the Scientific Revolution, which focused on recovering the ancients’ knowledge; **(iii)** 1632–1687, the Scientific Revolution, from Galileo’s *Dialogue* to Newton’s 1687 *Principia*; and **(iv)** 1687–1800, the age of Enlightenment.

Appendix Figs. E.1 and E.2 show QQ-plots for the marginal publications distribution in each historical period. The distribution for the set of fathers always first-order stochastically dominates that of scholars’ sons. That said, differences become smaller in the Scientific Revolution and in the Enlightenment. This suggests that, in later periods, the underlying human capital endowments determining publications were similar for fathers and sons selected into academia.

Our results show that these patterns emerged due to a decline in nepotism. Table 4 presents estimates of our model for each period separately.^32^ Before 1543, 48% of sons of scholars would not have become scholars under the same selection criteria as their fathers. This is reduced to 20.35 percent during the Scientific Revolution and to 8.3 percent during the Enlightenment. These percentages are precisely estimated and significantly different from each other: a Clogg et al. (1995)’s z-test rejects the null hypothesis of no difference in nepotism between the period before 1543, the Scientific Revolution, and the Enlightenment. In other words, in periods of rapid advancement, sons of scholars were selected more meritocratically. The dramatic differences in nepotism across time likely had large effects on the production of knowledge over time. To gauge this, we perform a counterfactual exercise where we replace all the nepotic scholars with average potential scholars. We find that this would increase the output of the average scholar in our sample by 69% before 1543, but only by 9% in the Enlightenment.

**Table 4:** Results over time

	$\beta$	$\gamma$	$\mu _h$	$\sigma _h$	$\sigma _e$	$\kappa$	N
Pre-Scientific Revolution,	0.18	48.82	-0.46	3.26	3.60	2.39	347
1088–1543	(0.14)	(10.42)	(0.93)	(0.82)	(1.21)	(0.53)	
Scientific Revolution (I),	0.62	20.35	1.63	4.28	0.21	2.01	385
1543–1632	(0.08)	(3.98)	(1.05)	(0.43)	(0.19)	(0.28)	
Scientific Revolution (II),	0.59	17.96	2.30	4.22	0.22	1.68	429
1633–1687	(0.08)	(2.74)	(0.68)	(0.30)	(0.14)	(0.22)	
Enlightenment,	0.67	8.29	3.77	3.47	0.38	2.34	673
1688–1800	(0.06)	(3.51)	(0.73)	(0.47)	(0.21)	(0.47)	
Institution established	0.63	19.48	1.61	4.13	0.49	2.22	730
pre-1543	(0.06)	(3.20)	(0.79)	(0.33)	(0.19)	(0.21)	
Institution established	0.61	8.80	3.93	3.59	0.38	1.88	760
post-1543	(0.05)	(2.16)	(0.42)	(0.26)	(0.10)	(0.26)	

SE in parenthesis obtained from 200 bootstrapped samples with replacement

Next, we turn to examine one of the mechanisms behind the decline in nepotism around the Scientific Revolution. The decline of nepotism could be the result of two processes: That *existing* universities and academies undertook structural reforms to eliminate nepotism from their hiring decisions; and/or that *new* institutions were established under more modern, meritocratic principles. The evidence supports the latter. In Table 4, we compare families of scholars in institutions established before vs. after 1543, the start of the Scientific Revolution. We only consider families who were active after 1543 such that both groups are comparable. We find that the percentage of sons hired under nepotism, $\gamma$, was substantially smaller in new institutions than in existing institutions which had been funded before the Scientific Revolution. Specifically, the percentage of nepotic sons are 19.5 for existing and 8.8 for new institutions, a difference that is statistically significant. This result is consistent with the historical narrative in Sect. 2, which suggests that the establishment of new academic institutions with more modern, meritocratic values was a key mechanism behind the modernization of academia in general, and behind the reduction of nepotism in particular.

Altogether, these estimates show that nepotism declined dramatically between 1088 and 1800. If seen as a witness of a broader downturn in favouritism towards relatives, friends, and acquaintances, the decline in nepotism is complementary with the accumulation of knowledge during the Scientific Revolution and the Enlightenment. In the next section, we provide more evidence in support of a relationship between changes in the knowledge frontier and the cost of nepotism.

Finally, we examine whether the father–son transmission of human capital changed over time. We find that, during the Scientific Revolution (1543–1632) and the Enlightenment (1715–1789), scholars inherited human capital endowments from their parents at a higher rate than pre-1543 scholars. Our $\beta$-estimate ranges from 0.18 before 1543 to 0.67 in 1688–1800, a difference that is statistically significant. This shows that, for individuals at the upper-tail of the human capital distribution, the intergenerational transmission of human capital is subject to changes in the environment and is not a universal constant as suggested by Clark (2015). Why would $\beta$ increase over time? On the one hand, $\beta$ captures the inheritability of skills, preferences, or genes, which is unlikely to vary much over time. On the other hand, $\beta$ also captures the transmission of other endowments which boost the sons’ research output—such as scientific knowledge, academia-specific human capital acquired at home, know-how on how to publish, on editors etc. These are endowments that can be transmitted at different rates in different periods. Although we cannot distinguish empirically between these two elements, the fact that our $\beta$-estimate increases over time suggests that the importance of academia-specific knowledge increased after the Scientific Revolution. That said, our $\beta$-estimate is relatively stable for a period of 450 years, from the start of the Scientific Revolution to 1800.

Interestingly, our estimates show an inverse relationship between nepotism, $\gamma$, and human capital transmission, $\beta$. In early academia, scholars used their influence to appoint their sons, even when these had low human capital. With the Scientific Revolution and the Enlightenment, nepotism faded but father–son pairs did not disappear. The reason is that sons of scholars inherited large human capital endowments from their parents, giving them a natural advantage over outsiders. In other words, lineages of scholars became more meritocratic. This suggests that the establishment of open universities and the emergence of meritocratic lineages in pre-industrial Europe was a stepping stone to the production of new ideas and the accumulation of upper-tail human capital.

## Heterogeneity and validation

### Heterogeneity

Here we explore heterogeneous effects with respect to universities’ religion, fields of study, changes in the knowledge frontier, whether sons were appointed during their father’s lifetime, and different types of academic institutions.^33^

*Protestant vs. catholic institutions* The Protestant Reformation is often associated to the rise of modern science. Merton (1938) argues that Protestant values encouraged the Scientific Revolution because science was seen as proof of God’s influence on the world. Others argue that, in Catholic countries, the Scientific Revolution was hindered by the Counter-Reformation (Lenski, 1963; Landes, 1998; Blasutto & De la Croix, 2023).^34^ We contribute to this debate by showing that differences in the scientific output of Protestant and Catholic universities are associated to differences in nepotism and human capital transfers within the family.

**Fig. 7:**
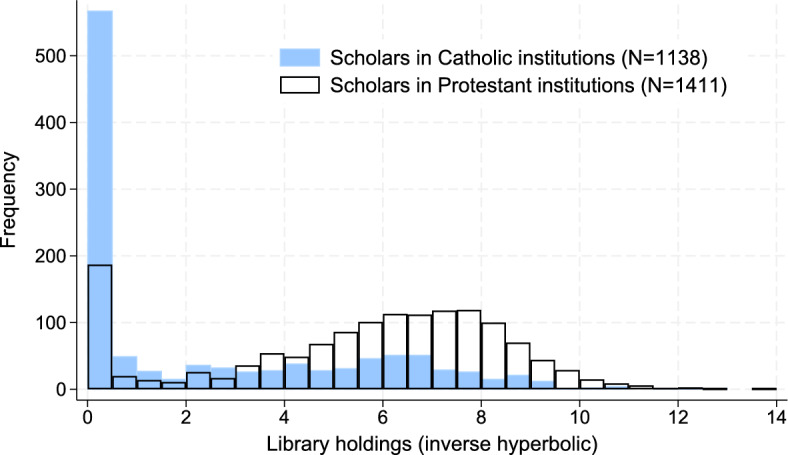
Publications, by institution’s religious affiliation. *Notes*: The sample are 2549 scholars nominated after 1527 who belong to a scholar’s lineage

We begin by showing that scholars in our dataset were more productive in Protestant than in Catholic institutions. To do so, we classify scholars according to the religious affiliation of their university or academy. We exclude all father–son pairs before 1527—when the first Protestant university, Marburg, was created. Figure 7 shows that the percentage of scholars with zero publications was 13.3% in Protestant institutions and 49.8% in Catholic institutions. Conditional on having at least one publication, the average scholar had more than foure times more publications in a Protestant institution than in a Catholic institution (298 vs 66 in levels). At the upper-tail of scientific production, there is a larger frequency of scholars with more than 1000 library holdings (ca. 7.6 arcsinh-publications) in Protestant institutions.

The larger scientific output in Protestant institutions is associated with a smaller prevalence of nepotism. Table 5, Panel A shows the estimated model’s parameters for Protestant and Catholic institutions separately. In Catholic institutions, 29.4% scholar’s sons were a by-product of nepotism; they would not have been selected into academia under the same criterium as their fathers. In contrast, in Protestant universities we only identify 6.6% of scholars’ sons as nepotic. These percentages are precisely estimated, and the z-test of Clogg et al. (1995) rejects the null hypothesis that our nepotism measure, $\gamma$, is equal in Catholic and Protestant institutions (see row 3 of Panel A). We also find that $\beta$ was 28 ppts larger in Catholic institutions, a difference that is statistically different from zero. In other words, Catholic institutions were more nepotic and relied more on the transmission of knowledge from fathers to sons in academia.

We find that differences in nepotism account for 16% of the Protestant-Catholic gap in publications in our data. Specifically, we perform a counterfactual exercise where we replace nepotic scholars with average potential scholars. This increases the publications of the average scholar by 41% in Catholic and by 7% in Protestant institutions. While the observed Protestant-Catholic gap in the son’s mean arcsinh-publications is 3.1, in this counterfactual scenario with no nepotism the gap is 2.6, which corresponds to a 16.1% reduction.

Note that many theology scholars were priests or pastors who could only be succeeded by their sons in Protestant institutions. In addition, nepotism was low in theology because appointments often required the approval of external Church authorities. We rule out that Protestant institutions appear more meritocratic because of this composition effect. To do so, we exclude theology scholars from the analysis (see row 4 of Panel A). The estimated percentage of nepotic sons, $\gamma$, is stable in Protestant institutions (5.0 vs. 6.6), and remains statistically different from the nepotism estimate in Catholic institutions (see row 5 of Panel A).

Altogether, these results suggest that nepotism and inherited human capital were relevant factors behind the decline of Catholic universities after the Protestant Reformation.

**Table 5:** Heterogeneity

	Intergen. HC elasticity	Nepotism, %	Other model parameters:				
	$\beta$	$\gamma$	$\mu _h$	$\sigma _h$	$\sigma _e$	$\kappa$	N
*A. Religion of university, post-1527*							
Catholic	0.77 (0.04)	29.4 (3.6)	-1.7 (0.8)	4.5 (0.3)	1.0 (0.4)	2.1 (0.2)	660
Protestant	0.49 (0.05)	6.6 (1.5)	4.6 (0.3)	3.4 (0.2)	0.3 (0.1)	1.5 (0.2)	838
Difference *p* value	[0.00]	[0.00]					
Protestant without theology	0.47 (0.06)	5.0 (2.0)	4.9 (0.4)	3.2 (0.3)	0.2 (0.1)	1.7 (0.5)	607
Difference *p* value	[0.00]	[0.00]					
*B. Field of study (of father)*							
Lawyer	0.71 (0.07)	33.8 (3.5)	-2.0 (0.7)	4.8 (0.5)	0.4 (0.3)	2.5 (0.4)	451
Physician	0.62 (0.07)	19.6 (3.4)	1.8 (0.7)	4.1 (0.3)	0.4 (0.2)	2.1 (0.2)	523
Theologian	0.55 (0.08)	12.0 (3.0)	3.6 (0.6)	3.9 (0.3)	0.4 (0.1)	1.1 (0.3)	259
Scientist	0.65 (0.07)	10.7 (3.3)	3.6 (0.7)	3.9 (0.4)	0.3 (0.1)	1.4 (0.4)	285
Father–son in same field	0.68 (0.05)	21.2 (2.2)	1.1 (0.7)	4.4 (0.3)	0.3 (0.2)	2.1 (0.1)	1341
Father–son in different field	0.56 (0.07)	14.8 (3.2)	3.0 (0.6)	4.0 (0.3)	0.2 (0.1)	1.9 (0.3)	496
Difference *p* value	[0.16]	[0.10]					
*C. Growth rate in publications*							
Rapidly growing knowledge frontier	0.64 (0.04)	9.2 (2.3)	3.7 (0.5)	3.5 (0.3)	0.3 (0.1)	2.1 (0.3)	1048
Stagnant knowledge frontier	0.78 (0.06)	25.3 (4.1)	-1.1 (1.3)	5.0 (0.5)	0.7 (0.2)	1.8 (0.3)	290
Difference *p* value	[0.05]	[0.00]					
*D. Appointment date of son*							
After father’s death	0.52 (0.06)	15.0 (2.6)	2.9 (0.5)	3.8 (0.2)	0.4 (0.1)	1.8 (0.2)	731
Before father’s death	0.72 (0.05)	21.4 (2.0)	0.8 (0.9)	4.9 (0.3)	0.4 (0.2)	1.6 (0.1)	777
Difference *p* value	[0.01]	[0.05]					
*E. Universities vs. Academies*							
Universities	0.68 (0.04)	16.5 (2.1)	2.1 (0.6)	4.2 (0.2)	0.4 (0.1)	1.9 (0.2)	1032
Academies	0.59 (0.07)	10.1 (3.5)	3.7 (0.6)	3.7 (0.4)	0.5 (0.2)	2.2 (0.4)	458
Difference *p* value	[0.26]	[0.12]					

*Notes:* Standard errors in parenthesis from 200 bootstrapped samples with replacement; *p* values in brackets from Clogg et al. (1995)’s z-test on null hypothesis of no differences in $\beta$ or nepotism

*Field of study* Next, we examine heterogeneity across fields of study. This is motivated because different types of upper-tail human capital can have different economic implications. For example, Murphy et al. (1991) and Maloney and Caicedo (2022) emphasize the importance of engineers for modern development. In medieval Europe, university training in Roman law helped to establish markets during the Commercial Revolution (Cantoni & Yuchtman, 2014). During the Scientific Revolution, research and teaching in science gained importance relative to philosophy, music, and history.^35^

Table 5, Panel B presents separate estimates for four fields of study: science (arts), law (canon and Roman law), medicine (including pharmacy and surgery), and theology.^36^ Father–son pairs are sorted into fields according to the father’s field. Our estimates show that nepotism was most prevalent in law faculties and among physicians: 33.8% of law scholars’ sons and 19.6% of physicians’ sons became scholars thanks to nepotism. This is in line with Lentz and Laband (1989), Mocetti (2016), and Raitano and Francesco (2018), who find high levels of nepotism for modern lawyers, pharmacists, and doctors. Differently, only 10.7% of scientists’ sons were nepotic scholars, suggesting that applied sciences were more open to newcomers. Although splitting the data by field of study reduces sample sizes, our nepotism estimates are precise, and the differences between fields are statistically significant. Finally, note that nepotism was low in theology. This reflects the fact that such appointments often required approval by Church authorities, and hence, universities had less discretion in filling this positions.

Next, we compare sons who followed their father’s footsteps in the same field with sons who published or taught in a different field than their fathers.^37^ This is interesting in two respects: First, one would expect sons in the same field to be less meritocratic—a son’s inherited social connections may be more important for obtaining a job in the same faculty as his father. Second, this exercise allows us to separate the transmission of general human capital from the transmission of human capital specific to the father’s field of study.

Table 5 presents the results. Families with fathers and sons in the same field were less meritocratic: 21.2% of sons in their father’s field became scholars because of nepotism; higher than the 14.8% of nepotism for fathers and sons in different fields. We also find a stronger transmission of human capital from fathers to sons in the same field, although $\beta$ is high for fathers and sons in different fields. This highlights the importance of general upper-tail human capital in our setting. Finally, these findings add credence to our identification strategy. It shows that the negative relation between nepotism, $\gamma$, and inherited human capital, $\beta$, over time is not an artificial by-product of our model or our estimation strategy. Where we expect both high transmission of human capital and high nepotism—such as among fathers and sons in the same field—our estimates for $\gamma$ and $\beta$ are positively related.

*Changing vs. stable knowledge frontier* Our results suggest that nepotism is more prevalent in stagnant environments (e.g., in catholic universities after 1527) than in dynamic societies or sectors experimenting structural changes (e.g., in scientific fields after the Scientific Revolution). This is consistent with the idea that under a rapid change in the knowledge frontier, technological progress, or cultural change, the cost from a mismatch between talents and occupation (i.e., nepotism) exceeds the benefits from the transmission of specific human capital from parents to children (i.e., a high $\beta$). The reverse holds true under a stable environment where the knowledge and social connections of one generation are still useful for the next. This idea has been examined in the context of the transmission of human capital and technological progress (Galor & Tsiddon, 1997), managerial capital in family firms (Carillo et al., 2019), and cultural persistence (Giuliano & Nunn, 2020).

Here we test this hypothesis in the context of premodern academia. We do so by estimating nepotism and the elasticity of human capital separately for families where the son entered academia at a time, society, and field of study that was rapidly changing vs. stagnant.

Specifically, we proceed in three steps. First, we use data from De la Croix (2021) on 40,800,000 publications of all known scholars active between 1500–1800 to calculate, for each year, the growth rate of publications over the previous 25 years by six fields of study: law, medicine, theology, humanities, science, and applied science. We further distinguish between field-specific growth rates in catholic and protestant institutions after 1527.^38^ We use an HP filter to smooth out short-run fluctuations and to preserve observations at the beginning and end of our time series. Appendix Fig. E.8 displays the different field-institution growth rates in publications over time. In general, theology, law, and humanities experienced eras of stagnation starting shortly before the 1600s, while applied sciences always display positive growth rates. All fields of study display a higher growth in protestant than catholic institutions, although theology and law become stagnant in protestant institutions after, respectively, the 1700s and the 1650s. Second, we classify families of scholars into two groups: those who worked at a time, society (catholic or protestant), and field of study experiencing rapid changes in the knowledge frontier vs. experiencing stagnation. In detail, we classify families into the first group if the field-institution growth rate in publications was positive at the time the son entered academia; and into the second group if the field-institution growth rate in publications was zero or negative at the time the son entered academia. Third, we estimate all our model’s parameters separately for these two groups.

Table 5, Panel C presents the results. Nepotism is less prevalent where the knowledge frontier was rapidly changing than where it was stable or stagnant. We find a nepotism estimate of 25.3% among scholars who were active at a time, society, and field with a stagnant production of knowledge. In contrast, only 9.2% of scholars were nepotic among those active in eras when their field was experiencing rapid changes in the knowledge frontier. These percentages are precisely estimated, and we reject the null hypothesis that nepotism was equal across these two groups (see row 3 of Panel C). We also find that the transmission of specific human capital from parents to children, $\beta$, was 14 percentage points larger in eras of stagnation than in eras of rapid change, a difference that is statistically significant. These results are robust to alternative groupings of families: Appendix Table E.1 shows similar results from a classification based on whether the field-institution growth rate in publications was above or below the median at the time the son entered academia (instead of above or below zero). Results are also robust to grouping families into rapidly changing vs. stagnant fields based on the date at which the father entered academia.

Overall, these results confirm the hypothesis that the observed decline in academic nepotism after the Scientific Revolution and in particular fields and institutions is complementary to a rapidly changing knowledge frontier. This raised the cost from a mismatch between talents and occupation, exceeding the benefits from the transmission of specific human capital from parents to children.

*Nomination before vs. after father’s death* A father may use his social connections to nominate his son to a chair, or secure a university chair as part of his family’s assets and pass it down to his son upon his death. We distinguish these two expressions of nepotism by estimating our model for father–son pairs in which the son was nominated before vs. after his father’s death. Table 5, Panel D shows that 21.4% of sons nominated during their father’s lifetime were nepotic scholars. Alternatively, we find nepotism in 15% of sons nominated after their father’s death. The z-test of Clogg et al. (1995) can reject the null hypothesis of no difference with a *p* value of 0.05. This suggests that, in our setting, nepotism is characterized mostly by fathers using their social connections to nominate their sons, but also by fathers passing down their chairs as part of the inheritance.

*Universities vs. academies* Academies were often seen as superior institutions than universities. Many outstanding scholars joined the academies created during the Scientific Revolution, e.g., Royal Society of London (1662), Académie des Sciences (1666), and the Leopoldina (1677). These academies formalized the Republic of Letters and were an engine of cultural change (Mokyr, 2016). Table 5, Panel E compares families of scholars in universities vs. academies after 1543, the start of the Scientific Revolution. Our findings do not support the negative views about universities: both the father–son transmission of human capital ($\beta$) and the percentage of nepotism ($\gamma$) are not statistically different in universities vs. academies. This suggests that nepotism declined after the Scientific Revolution in academies, but also in newly established universities.

### Validation using outsider scholars

So far, our analysis has focused on comparing the publications of scholars’ sons and their fathers. Here we show that our results are consistent with comparisons between scholars’ sons and the universe of outsiders in academia. That is, scholars who did not belong to a family dynasty. Figure 1 already showed some preliminary evidence that our main findings are consistent with rough comparisons of the research productivity of the average scholar’s son and the average outsider over time. Here we further validate our main findings by extending our estimation strategy to incorporate outsiders and showing that our results are unchanged. Specifically, we now use data on outsiders and quantify the nepotistic behavior of fathers in favor of their children by comparing the selection criteria (i.e., entry barriers) faced by sons of scholars with those applied to outsiders in the same generation.

In order to conduct this exercise, we need to extend our estimation strategy in three dimensions. First, we extend our theoretical model to incorporate outsiders. As before, our model economy consists of a population of potential scholars whose unobserved human capital is transmitted from fathers to sons with an elasticity of $\beta$ (Eqs. (1) and (2)), and transformed into publications with measurement error noise (Eqs. (4) and (5)). Potential scholars with human capital endowments above $\tau$ become scholars. We now allow the selection criterium to be different for sons of scholars, not only relative to their fathers (Eq. (3)) but also relative to outsiders in the same cohort who do not have family connections in academia. Formally, we extend Eq. (3) as follows:

11 $$\begin{aligned} \mathbb {P}=\left\{ i \mid h_{i,t}>\tau , h_{i,t+1}>\tau -\hat{\nu }\right\} \end{aligned}$$

12 $$\begin{aligned} \mathbb {O}=\left\{ i \notin \mathbb {P} \mid h_{i,t+1}>\tau \right\} , \ \ \ \ \ \ \end{aligned}$$

where $\mathbb {P}$ denotes the set of father–son pairs in academia and $\mathbb {O}$ the set of outsiders. Specifically, sons of scholars are individuals in generation $t+1$ who fulfill the (nepotic) criterium to become scholars, $h_{i,t+1}>\tau -\hat{\nu }$, and whose fathers are in academia, $h_{i,t}>\tau$. Outsiders are individuals in generation $t+1$ who fulfill the criterium to be scholars, $h_{i,t+1}>\tau$, and who do not have family connections in academia in the previous generation, $i \notin \mathbb {P}$.

This changes the interpretation of $\hat{\nu }$, which now measures the distance in human capital between the marginal scholars’ son and the marginal outsider *in their same generation*, i.e., $\hat{\nu }=\tilde{h}_{i\in \mathbb {O}, \ t+1}-\tilde{h}_{i\in \mathbb {P}, \ t+1}$. That is, $\hat{\nu }$ captures how much scholars’ sons are favored relative to all their potential competitors in the same cohorts who do not have any family connection.^39^ Similarly, we define the magnitude of nepotism, $\hat{\gamma }$, as the share of sons in academia who would not have become scholars under the same selection criterium as outsiders in the same cohorts. We recover $\gamma$ from Eq. (9), which now is based on this modified interpretation of the entry barriers to academia for sons of scholars, $\hat{\nu }$.^40^

The second extension to our estimation strategy is to use data not only on families of scholars but also on the universe of all known scholars, including outsiders. We select the relevant set of outsiders by applying two sample restrictions: On the one hand, we consider only outsiders who started working in the same decade and institution as at least one scholar’s son. This restriction is done such that we effectively recover nepotism by comparing sons of scholars and their potential competitors in the same cohorts. On the other hand, scholars belonging to a dynasty tend to be better documented than outsiders. For example, conditional on the number of publications, cohort, field, and institution fixed effects, a scholar’s son is more likely to have a Wikipedia page than an outsider (see Appendix Table E.2). To increase the comparability between the two groups and make sure that our results are not driven by this difference, we take a conservative approach and consider only outsiders and families of scholars who are listed in Worldcat or Wikipedia.

**Fig. 8:**
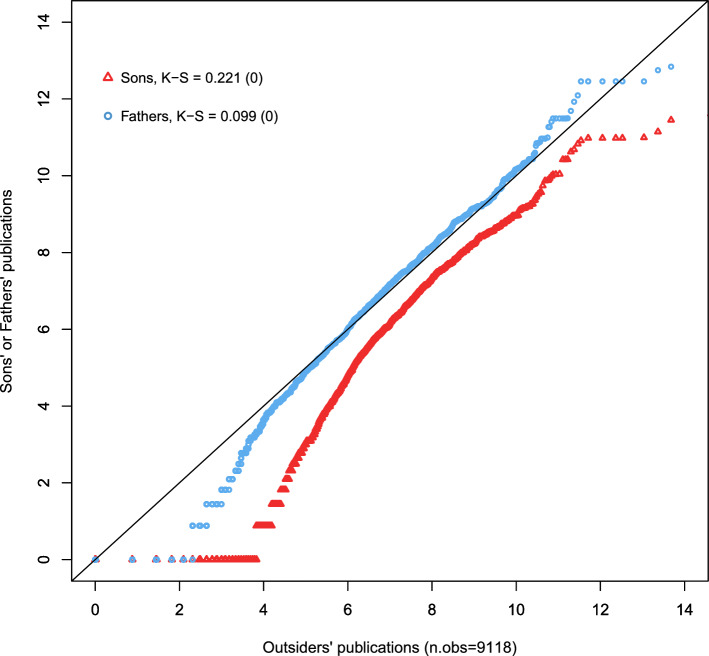
Quantile-quantile plot of outsiders, fathers, and sons in academia. *Notes*: The sample are 1482 families of scholars and 9118 outsiders listed in Worldcat or Wikipedia. Outsiders are restricted to those entering academia in the same decade and institution as a scholar’s son. Publications are the inverse hyperbolic sine of library holdings by or about each author

The third extension to our estimation strategy concerns the targeted moments. In addition to our two baseline sets of moments—intergenerational correlations and father–son distributional differences—we now also target differences in the publications’ distribution of scholar’s sons, $f(y_{i \in \mathbb {P}, \ t+1})$, and outsiders, $f(y_{i \in \mathbb {O}, \ t+1})$, in the same generation $t+1$. In detail, we target our baseline 13 moments and 5 additional moments: the share of outsiders with zero publications, the mean, median, 75th and 95th percentile of the outsiders’ publications distribution. These moments are illustrated in Fig. 8 (see Appendix Table E.3 for all moments). It shows a QQ plot of the quantiles of the publications’ distribution of outsiders (x-axis) against the quantiles of scholar’s sons in the same cohorts and institutions, as well as their fathers (y-axis). The publications’ distribution of outsiders and fathers is similar, as most quantiles lie on the 45 degree line. In other words, outsiders and fathers (i.e., first-generation scholars) had similar research productivity, which suggests that they were subject to similar selection criteria into academia. In contrast, in all quantiles, fathers and outsiders have larger publication records than scholar’s sons. That is, the outsider’s publication distribution FOSD that of scholars’ sons competing in the same cohorts and institutions (Fact 3). This provides some preliminary evidence that incorporating outsiders into the analysis will not substantially alter the nepotism estimates obtained from comparisons between the set of sons and fathers in academia. To confirm this hypothesis, we use the additional Fact 3, together with the two Facts on fathers and sons described before (see Sect. 3), to estimate nepotism and the intergenerational human capital elasticity.

Formally, we identify the six parameters in our extended model ($\beta , \hat{\gamma }, \mu _h, \sigma _h, \sigma _e, \kappa$) by minimizing the distance between these 18 simulated and empirical moments, using an objective function analogous to Eq. (10).^41^ As before, intergenerational correlations and father–son distributional differences allow us to estimate the intergenerational human capital elasticity between fathers and sons, net of measurement error in how observed outcomes (publications) reflect unobserved endowments (human capital). Importantly, we now recover the nepotism parameters ($\hat{\gamma }$, which in turn is based on $\hat{\nu }$) from the distributional differences between the publications of sons of scholars and ousiders who were active in the same decade and institution. In our baseline model, these were estimated solely from excess father–son distributional differences net of mean reversion.

Table 6 presents the results. Column (1) reports estimates from our baseline strategy comparing fathers and sons. Note that the sample and results are different than those in Sect. 5.2 because of the additional sample restrictions described above. The share of nepotic sons is 14.38%, in the lower range of estimates in Table 3. Column (2) reports estimates from our extended strategy, which recovers the different entry barriers ($\hat{\nu }$) and nepotism ($\hat{\gamma }$) by comparing sons of scholars and outsiders who were active in the same decade and institution. The share of nepotic sons is 14.55%, identical to the estimate obtained from our baseline strategy comparing fathers and sons. Similarly, the parameters $\nu$ in the baseline estimation and $\hat{\nu }$ in the estimation with outsiders are similar and not statistically different from each other.^42^ This suggests that the entry barriers faced by outsiders relative to scholars’ sons in the same generation were similar to the the entry barriers faced by first-generation scholars relative to their sons one generation later. We find no significant differences in the estimated intergenerational elasticity of human capital, $\beta$, in our baseline (0.572) vs. extended estimation using outsiders (0.562). The other model’s parameters capturing the noise with which unobserved human capital is transformed into observed publications ($\sigma _e$ and $\kappa$), and the shape of the human capital distribution ($\mu _h$ and $\sigma _h$) are also not significantly different across strategies.

**Table 6:** Identified parameters using estimation strategy with outsiders

		Baseline estimation	Estimation with outsiders	Difference
		[1]	[2]	[3]
IGE human capital	$\beta$	0.572 (0.04)	0.562 (0.04)	−0.010 [0.919]
Nepotism, %	$\gamma$	14.38 (1.76)	14.55 (1.65)	0.170 [0.944]
Mean human capital	$\mu _h$	3.116 (0.36)	3.425 (0.35)	0.309 [0.536]
SD human capital	$\sigma _h$	4.079 (0.20)	4.510 (0.21)	0.431 [0.131]
SD publications’ shock	$\sigma _e$	0.309 (0.13)	0.248 (0.09)	−0.061 [0.697]
Threshold publications	$\kappa$	1.268 (0.14)	1.354 (0.14)	0.086 [0.655]
Entry barriers	$\nu$ or $\hat{\nu }$	8.954 (1.18)	8.850 (0.57)	−0.104 [0.937]

*Notes:* Col. [1] targets 13 intergenerational correlations and fathers’ and sons’ distributional moments (N=1,482); Col. [2] targets, in addition, 5 outsiders’ distributional moments (N=9,118); SE in parenthesis from 200 bootstrapped samples with replacement; *p* values in brackets from Clogg et al. (1995)

Altogether, our estimates are robust to comparing the entry barriers faced by scholar’s sons and outsiders in the same generation. This strongly suggest that our baseline estimation strategy comparing scholars’ sons and their fathers provides a credible characterization of nepotism and inherited human capital transfers in premodern academia.

### Validation using families at different universities

In this section we perform a validation test on an alternative sample where, *ex ante*, we expect less nepotism: fathers and sons at different institutions. Social connections may be more important for obtaining a job where one’s father is employed than in a different university or academy. Hence, sons appointed at a different institution than their father were more likely to be hired meritocratically.

We estimate our model for an alternative sample of 507 scholars appointed to at least one different university or academy than their fathers. 63.5% of these father–son pairs are also in the baseline sample—that is, they held positions in the same and in different institutions. The remaining 36.5% were never in the same institution. Since we expect these father–son pairs to be more meritocratic, a large estimate for the nepotism parameter would falsify our identification strategy. It would suggest that our nepotism parameter reflects sampling issues or that it captures other elements of the university’s hiring process—e.g., information frictions affecting scholars’ sons and outsiders differently. A large nepotism estimate could also suggest that broader trends outside academia—to which both our baseline and validation sample are exposed—are important for our results over time.

Appendix Table E.4 provides the empirical moments and the estimates for this alternative sample. As expected, fathers and sons appointed to different institutions have a better publication record: the share with zero publications is lower, and the mean, median, and 75th percentile are higher than for fathers and sons in our baseline sample. In addition, the distribution of publications of fathers no longer first-order stochastically dominates that of sons.

We find that nepotism was negligible in this alternative sample: only 0.04% of sons appointed to a different institution than their fathers were hired because of nepotism. This estimate is statistically significantly lower for fathers and sons in different vs. in the same institution. In addition, fathers and sons in different institutions transmitted their human capital with an elasticity of 0.81, not significantly different than our baseline elasticity (0.63).

Admittedly, these two sets of families are different in other dimensions, and successful professors may have had some sway in placing their sons in other institutions. That said, other than validating our identification strategy, this result is interesting in its own right. It shows that mobile families of scholars, where fathers and sons worked in different institutions, were not the result of nepotism. This supports the hypothesis that the establishment of an academic market with hiring across universities (De la Croix et al., 2023) might have fostered modern, open universities not subject to nepotism.

### Comparison to other methods to estimate intergenerational persistence

Here we compare our $\beta$-estimates to those obtained using alternative methods in the literature. As explained above, our estimate is consistent with Clark’s hypothesis that endowments transmitted across generations are more persistent than suggested by parent–child correlations in outcomes, but falls at the bottom of the range of estimates using rare surname groups (Clark, 2015), multi-generation correlations (e.g., Braun and Stuhler 2018), and the informational content of surnames (e.g., Güell, RodríguezMora, and Telmer 2015). All these methods address the measurement error bias in parent–child correlations, but ignore selection in the form of nepotism (see Appendix B). To evaluate if our different estimate reflect the importance of addressing selection in the form of nepotism or is just a byproduct of the specifics of our setting, we use our data on father–son scholars to estimate intergenerational elasticities using standard methods in the literature.

First, we estimate a standard log-log elasticity:

13 $$\begin{aligned} y_{i,t+1} = b \ y_{i,t} + e_{i,t+1} \ , \end{aligned}$$

where *y* is an outcome for fathers, *t*, and sons, $t+1$. In our setting, *y* is the logarithm of 1 + the number of library holdings.

Second, we estimate rank-rank slopes as proposed by Chetty et al. (2014). We rank scholars’ sons based on their publications relative to other scholars’ sons in the same 50-year birth cohort. We then rank scholars’ fathers based on their publications relative to other scholars’ fathers with sons in these 50-year birth cohorts. We estimate the rank-rank slope by regressing the son’s percentile-rank in publications on their father’s percentile-rank in publications.

Table 7 presents the results. We find a log-log elasticity, $\hat{b}$, of 0.46. This implies that a 1% increase in a father’s publications is associated with a 0.46% increase in his son’s publications.^43^ The log-log elasticity for fathers and sons with at least one publication, that is, the elasticity in the intensive margin, is $b_I=0.36$. The rank-rank slope estimates are very similar: $\rho _{PR}=0.49$ for all scholars and of $\rho _{PR,I}=0.39$ for fathers and sons with at least one publication. In comparison, our model’s $\beta$-estimate is larger than both the log-log elasticities and the rank-rank slopes estimated with our data.^44^ This suggests that our larger intergenerational elasticity estimates do not only stem from the specifics of our setting, but also reflect methodological differences. Specifically, they reflect that the measurement error in father–son log-log elasticities and rank-rank slopes can attenuate intergenerational estimates.

**Table 7:** Intergenerational elasticites amongs scholars, different methods

Method		Value	s.e.	N	References
Log-log elasticity, all	$\hat{b}$	0.46	0.019	1837	Equation (13)
Rank-rank slope, all	$\rho _{PR}$	0.49	0.022	1837	Chetty et al. (2014)
Log-log elasticity, intensive margin	$\hat{b}_I$	0.36	0.028	982	Equation (13)
Rank-rank slope, intensive margin	$\rho _{PR,I}$	0.39	0.027	982	Chetty et al. (2014)
Multiple-generations’ ratio	$\hat{\beta }$	0.91	0.077	216	Braun and Stuhler (2018)
Multiple-generations’ ratio	$\hat{\beta }_A$	0.79	0.070	216	Braun and Stuhler (2018)
Model’s $\beta$	$\beta$	0.63	0.042	1837	–

*Notes:* The sample are 1837 scholars and their fathers. In rows 3 and 4, this is restricted to 982 families in which both father and son have at least one publication. Rank-rank elasticities estimated from Eq. (13) using a scholar’s percentile-rank in publications within 50-year birth cohorts instead of his log-publications. In rows 5 and 6, the sample are 216 scholars (G3), their fathers (G2), and grandfathers (G1); $\hat{\beta }~=~b_{_{G1-G3}} \ / \ b_{_{G2-G3}}$ and $\hat{\beta }_A~=~b_{_{G1-G3}} \ / \ average\left( b_{_{G1-G2}}, b_{_{G2-G3}} \right)$, where $b_{_{Gi-Gj}}~=~ cov(y_{_{Gi}}, y_{_{Gj}}) \ / \ var(y_{_{Gi}})$ is the elasticity of publications between generations *Gi* and *Gj*. Bootstrapped standard errors

Third, we estimate multiple-generations’ methods proposed by Braun and Stuhler (2018) to address measurement error. They consider a Markov process as in Eq. (2), where the endowments transmitted across generations, *h*, are not observed and are normally distributed with a mean $\mu _h$ and a variance $\sigma _h^2$. As in our setting, these unobserved endowments are transformed into observable outcomes *y* with measurement error: $y_{i,t} = h_{i,t}+ \varepsilon _{i,t}$, where $\varepsilon _{i,t+1}$ is an independent noise term with a standard deviation of $\sigma _\varepsilon$.^45^ Differently from us, they do not consider selection in the form of nepotism (Eq. (3)). Under their framework, the elasticity in outcomes across *n* generations is $\beta ^n \ \theta$, where $\beta$ is the intergenerational transmission of endowments and $\theta = \sigma _h^2 \ / \ \left( \sigma _h^2+\sigma _\varepsilon ^2 \right)$ is the measurement error bias. As $\theta <1$, this is an attenuation bias. To correct for it and identify $\beta$, they use the ratio between the grandfather-grandson elasticity ($\beta ^2 \theta$) and the father–son elasticity ($\beta \theta$).

Table 7 presents estimates for this ratio using our sample of 176 families with three generations. These families contain 216 scholars (generation 3) with their fathers (generation 2) and one of their grandfathers (generation 1) in academia.^46^ We report estimates of $\hat{\beta }$, the ratio of the elasticity between generations 1 and 3 to the elasticity between generations 2 and 3. We also report $\hat{\beta _A}$, the ratio of the elasticity between generations 1 and 3 to the average elasticity between generations 2 and 3 and generations 1 and 2. These methods yield a $\beta$ between 0.91 and 0.79, a larger value than our model-based $\beta$ and closer to the estimates of Clark (2015). This suggests that in empirical applications where selection is relevant, as is our case, the multiple-generation methods in the literature can provide upward-biased $\beta$-estimates.

Addressing this selection bias is important for studies of the intergenerational transmission of occupations, especially where nepotism is commonplace. That said, even in empirical applications where nepotism is absent, the type of entry barriers/selection bias described here may also affect intergenerational elasticities. Specifically, long-run estimates of the intergenerational elasticity of wealth, earnings, or occupations typically rely on selected samples, such as probate records—where only those leaving wealth above a legal threshold are sampled (Clark & Cummins, 2015), or ancestors and descendants in a particular city—where only non-migrants are sampled (Barone & Mocetti, 2020; Häner & Schaltegger, 2024). Although these selection processes are different in nature to nepotism, they are related to the inherited endowments, and hence, can potentially lead to similar selection biases in intergenerational elasticities. For empirical applications studying the transmission of years of schooling (e.g., Braun and Stuhler (2018),Lindahl et al. (2015)) selection can take on different forms. For example, the inherited connections and social circles of sons may facilitate their access to more prestigious, post-graduate institutions ahead of better suited candidates. Moreover, even if these empirical applications typically use census data covering the population, families are not sampled if intergenerational links are not observed, e.g., because children emigrate or die before observable outcomes *y* (e.g., income, years of education) are realized. These estimates are potentially subject to a selection bias as the one described above, since whether observations are sampled or not (attrition) can be correlated with unobserved endowments *h* inherited by children (e.g., if there is negative selection into migration by parental endowments).

## Robustness

We perform several additional robustness checks. This section briefly describes them; the detailed results are available in the Appendix.

*Stationarity* Our estimation assumes that the human capital of fathers and sons in the population of *potential scholars* is drawn from the same distribution. This stationarity assumption is standard in the literature, but its importance to estimate intergenerational elasticities is rarely discussed (Nybom & Stuhler, 2019). In Sect. 5.3, we relax this assumption. Specifically, we assume that a father and a son who were active in a given historical period draw their human capital from the same distribution, but we allow the human capital distribution to change across periods. Hence, we allow publications to exhibit time trends on both the extensive or intensive margin. In addition, Appendix G examines the stationarity assumption further. First, it examines trends among potential scholars using the De la Croix (2021) dataset on all known scholars (not only fathers and sons). The mean and the standard error of publications, our proxy for human capital, are stable over time, suggesting a stationary human capital distribution. The probability of being listed in WorldCat changes around 1450, but this break is related to the introduction of the printing press rather than to changes in the human capital distribution, and is accounted by in our estimation by the $\kappa$ parameter (see Sect. 5.3). Second, the appendix shows that under stationarity our nepotism estimates are conservative, lower-bound estimates. The reason is that our estimation uses distributional differences to identify nepotism but does not attribute all these differences to it. We allow for distributional differences to be the result of a second force: mean reversion. That is, that top scholar’s sons may be “naturally” worse than their fathers, even if no nepotism is involved. In a non-stationary environment where the human capital distribution improves over time, mean reversion would explain less of the father–son distributional differences in publications. Hence, under a non-stationary environment, our nepotism estimates would be larger.

*Shocks from fat-tailed distributions* Like most of the literature, we draw shocks affecting human capital from a normal distribution. An attractive alternative consists in drawing shocks from fat-tailed distributions, giving higher likelihood to the emergence of geniuses. In Appendix H we re-estimate our model under different distributional assumptions. We show that, although fat tailed distributions for human capital shocks seem *a priori* to be appealing, they do not fit the data well, which is very normally distributed after all. Our nepotism estimates are however robust to assuming fat-tailed shocks, although the estimated intergenerational persistence is not.

*Linear human capital transfers* We assume that $\beta$ is linear, that is, that parents at the top and bottom of the distribution transmit their endowments at the same rate. This assumption would be violated, e.g., if successful fathers spent less time with their children, reducing their human capital transfers systematically.^47^ Appendix I shows evidence supporting our assumption. Specifically, OLS elasticity estimates are identical to elasticities estimated non-parametrically. The latter allow elasticities to differ across families with different publication records, and hence, with different human capital endowments.

*Publication threshold* To capture measurement error on the extensive margin, our model considers $\kappa$, the minimum number of works to observe a scholar’s publications. Admittedly, this parameter may differ across scholars. For example, the work of the son of a famous scholar may capture the attention of publishers more easily—even if it is of lower quality. Appendix J examines whether this can explain away our results on nepotism. We re-estimate our model allowing the publication threshold $\kappa$ to be lower for scholars’ sons. Our estimates are robust to this modification.

*Measure of publications* Our preferred measure of the size and relevance of a scholar’s output is the total number of library holdings in modern libraries by or about each scholar. This includes all the copies of work written by a scholar, but also library holdings about his work written by a different author. In Appendix K, we show that our results are robust to excluding library holdings about his work written by a different author, and to using the number of unique works by or about a scholar instead of the total library holdings. Using these two alternative measures suggests that 18.7 and 18.8% of scholars’ sons were nepotic, indistinguishable from our baseline result of 18.7%. The $\beta$ estimates are also similar across measures (respectively, 0.63, 0.62, and 0.61).^48^

*Longevity* On average, scholar’s sons in our sample died at age 61.7, six years earlier than their fathers. Since longevity is important for publications, Appendix L shows that our results are not driven by this differential longevity. We use OLS and simultaneous-quantile regressions to estimate the marginal effect of living an additional year on the mean, median, 75th and 95th percentile and on the proportion of sons with zero publications. We then use these estimates to adjust the distributional moments for the set of sons. The adjusted and baseline moments are very similar. Even after accounting for longevity differentials, the fathers’ publications distribution FOSD that of sons, especially at the bottom of the distribution (*Fact 2*). This strongly suggests that our nepotism and $\beta$-estimates are not driven by differences in longevity.

*Fertility differentials* Appendix M discusses the sensitivity of Fact 2 and our nepotism estimates to fertility differentials between scholars with more and less publications, and shows that estimates are unchanged when we exclude scholars with more than one son in academia.

## Conclusion

From the Bernoullis to the Eulers, families of scholars have been common in academia since the foundation of the first university in 1088. In this paper, we have shown that this was the result of two factors: Initially, scholars’ sons benefited from their fathers’ connections to get jobs at their fathers’ university. Between 1088 and 1543, about one in two scholars’ sons benefited from nepotism. They became academics even when their underlying human capital was lower than that of marginal first-generation scholar. After the Scientific Revolution, nepotism faded but families remained in academia. The reason is that scholars transmitted to their sons a set of underlying endowments, i.e., human capital, that were crucial to produce scientific knowledge. Our estimates suggest a large intergenerational elasticity of such endowments, as high as 0.6–0.65.

To disentangle the importance of nepotism vs. inherited human capital endowments, we proposed a new method to characterize intergenerational persistence. Our method exploits two sets of moments: one standard in the literature—correlations in observed outcomes across multiple generations—another novel—distributional differences between adjacent generations in the same occupation. Under a standard Becker and Tomes (1979) model of intergenerational human capital transmission, a slow rate of reversion to the mean strengthens the correlations across generations and (should) reduce the distributional differences between fathers and sons. When these distributional differences are larger than predicted by reversion to the mean, it reflects the fact that parents and children are selected under different criteria, i.e., nepotism. In other words, excess parent–child distributional differences within a top occupation can be used to identify and to quantify the prevalence of nepotism.

Our results have two important implications for measuring the rate of intergenerational persistence. First, we argue that estimates that bundle the transmission of human capital and social connections may provide biased estimates of the true rate of intergenerational persistence. The reason is that each of these two elements is associated with a different econometric bias: measurement error and selection. Our estimate for the transmission of human capital endowments is higher than estimates ignoring both biases—i.e., parent–child correlations—but in the lower range of estimates ignoring selection—i.e., multi-generational correlations, group averages, or the informational content of surnames. Specifically, when we omit nepotism, we find large intergenerational human capital elasticities among scholars, close to the 0.8–0.9 range estimated by Clark (2015). Hence, failing to account for selection can overstate the true rate of persistence of underlying human capital endowments. This problem is particularly acute in historical studies of social mobility over the very long run, which typically rely on selected samples.

Second, our proposed method circumvents some of the data requirements that have limited the study of intergenerational persistence in historical contexts. By modelling selection explicitly, our method only requires data from a well-defined universe, for example, a top occupation. Historical data of such occupations, e.g., scholars, artisans, artists, or government officers, is more common than the census-type evidence required by some of the alternative methods in the literature (Güell, Rodríguez Mora, and Telmer 2015, Lindahl et al. 2015, Braun and Stuhler 2018, Collado, Ortuno-Ortin, and Stuhler 2018). In addition, we build a novel dataset with direct links across generations over 1088–1800. This allows us to overcome the empirical challenges associated with using surname pseudo-links to estimate intergenerational elasticities over centuries Clark 2015. Finally, relative to the literature examining the concentration of certain families in top occupations, our approach allows us to estimate nepotism across time and space, beyond the specific settings where a natural experiment is available.

Finally, this paper sheds new light on the production of upper-tail human capital and its importance for pre-industrial Europe’s take-off (Cantoni and Yuchtman 2014, Mokyr 2002, 2016, Squicciarini and Voigtländer 2015, De la Croix, Doepke, and Mokyr 2018). We find that the transmission of human capital within the family and nepotism follow an inverse relationship over time. Periods of advancement in sciences, like the Scientific Revolution or the Enlightenment, are associated with less nepotism in universities and scientific academies. In contrast, nepotism is prevalent in periods of stagnation and in Catholic institutions that fell behind in the production of scientific knowledge. This is consistent with the idea that in eras of rapid change in the knowledge frontier, technological progress, or cultural change, the cost from a mismatch between talents and occupation caused by nepotism exceeds the benefits from the transmission of specific human capital from parents to children (Galor & Tsiddon, 1997; Carillo et al., 2019).

Although nepotism only concerns fathers and sons, it is likely to reflect other forms of favouritism towards relatives, friends, and acquaintances. Hence, the high levels of nepotism might reflect broader inefficiencies and talent misallocation in early academia. Altogether, our evidence suggests that during the Scientific Revolution and the Enlightenment some of these inefficiencies were removed and that the resulting modern, open universities contributed to Europe’s scientific advancements. The extent to which these changes explain Europe’s rise to riches is an intriguing question for future research.

## Supplementary Information

Below is the link to the electronic supplementary material.

Supplementary file 1 (pdf 13503 KB)

## Footnotes

1 Cantoni and Yuchtman (2014), Applebaum (2003), and Mokyr (2010), respectively.

2 In historical settings where access to education was limited, a crucial element for human capital formation was growing up in a household where there were books. In premodern Europe, scholars’ households were amongst the few owning books; attesting to the historical importance of the human capital transmitted from parents to children.

3 Although the term “meritocracy” is a modern construct, its virtues are known since Plato and Confucius (Wooldridge, 2021).

4 For example, Lindahl et al. (2015) show that human capital endowments are more persistent across multiple generations than suggested by parent–child elasticities.

5 Clark (2015); Häner and Schaltegger (2024); Barone and Mocetti (2020).

6 This corresponds to 100–200 fewer library holdings in modern libraries.

7 See Solon (1999), Corak (2006), and Black and Devereux (2011) for reviews.

8 Examples are doctors (Lentz & Laband, 1989), lawyers (Laband & Lentz, 1992; Raitano & Francesco, 2018), politicians (Dal Bó et al., 2009), inventors (Bell et al., 2018), CEOs (Pérez-González, 2006; Bennedsen et al., 2007), pharmacists (Mocetti, 2016), self-employed (Dunn & Holtz-Eakin, 2000), and liberal professions (Aina & Nicoletti, 2018; Mocetti et al., 2018).

9 Only two articles studied premodern social mobility using direct parent–child links: Shiue (2017) for the Tongcheng county in China (1300–1900), and Mulder et al. (2009) for East Anglia (1540–1845).

10 A related literature studies if human capital is genetically inherited (selection) or depends on parents’ investments (causation) (Holmlund et al., 2011). Differently, we address the selection bias from nepotism to disentangle it from human capital transfers, but not whether the latter reflect nature or nurture.

11 In Plato’s Republic, children in the ideal society were raised by the state to undermine their nepotistic preferences for their relatives (Ciulla, 2005).

12 Robert Stelter digitized chair holders at the University of Tübingen and Alice Fabre lawyers and rectors at the University of Avignon (see De la Croix et al. 2023).

13 For example, we have a complete coverage for the University of Macerata—a small university in Italy, while there is no comprehensive catalogue of professors for the University of Paris.

14 For example, the Chicoyneau and Mögling families had, respectively, four and six generations of scholars at the University of Montpellier and at Tübingen. See Figs. A2 and A3 in Appendix A for illustration.

15 This figure is based on 20,500 scholars from De la Croix (2021). See Sect. 6.2 for details.

16 WorldCat identifies publications about a scholar’s work by another author if the scholar’s name appears in any metadata associated with the publications, such as title, dedicatee, subject, or other authors. A common example of this are compendia of a scholar’s work written or edited by another author.

17 Importantly, in our setting the arcsinh and log-distributions of the number of library holdings behave identically, and hence, we can interpret arcsinh-arcsinh specifications as elasticities. This is because the number of library holdings (in levels) of fathers and sons take on large values, with means well-above the 10 threshold proposed by Bellemare and Wichman (2020).

18 *Les Bouches-du-Rhône, Encyclopédie Départementale*, by Masson (1931).

19 Changes in the human capital distribution would affect trends on both the extensive and intensive margin.

20 In magnitude, the father–son correlation in academia is similar to the elasticity in wealth in premodern populations (Mulder et al., 2009), and in education attainment in modern Sweden (Lindahl et al., 2015).

21 The differences in levels are $sinh(4.456)=43.07$ vs. $sinh(3.477)=16.17$ in the mean and $sinh(5.075)=79.983$ vs. $sinh(3.402)=14.995$ in the median.

22 In our empirical application we do not observe mothers. Under the assumption of positive assortative matching, the endowment inherited from father and mother is similar.

23 For simplicity, we assume that $h_{i,t}$ and $h_{i,t+1}$ are in logarithms.

24 Note that $\tau - \nu$ alone does not characterize the magnitude of nepotism. For example, the same $\tau - \nu$ can reflect low levels of nepotism if the mean $\mu _h$ and the variance $\sigma _h^2$ of the human capital distribution are high, and high levels of nepotism if $\mu _h$ and $\sigma _h^2$ are low (see Appendix Fig. C.1 for an example).

25 Consider the Markov process in Eq. (2) without selection. The elasticity of *y* between parents (*t*) and children ($t+1$) is $\beta \theta$, with $\theta = \sigma _h^2 / (\sigma _h^2+\sigma _\varepsilon ^2 )$. The elasticity of *y* between grandparents (*t*) and grandchildren ($t+2$) is $\beta ^2 \theta$. If measurement error is constant across generations, the ratio of these elasticities identifies $\beta$.

26 In addition, the variance of the fathers’ and sons’ distributions—captured by the 75th and 95th percentiles—allows us to disentangle measurement error from nepotism: Larger measurement error increases the variance of both distributions, while larger nepotism increases the variance of the sons’ distribution more. This allows us to address measurement error without resort to grandfather-grandson correlations (see Appendix C).

27 We calculate the distributional measures on all fathers and sons based on the inverse hyperbolic sine of library holdings. We do so such that these measures are analogous to intergenerational correlations, which are based on this transformation.

28 Sect. 2 shows that the geographical coverage is extense and not associated with the data coverage (complete, broad, or partial), suggesting that selection of universities and academies into our sample is not a source of bias.

29 Solon (1989) shows that sampling bias tends to attenuate intergenerational elasticities. Our estimates are lowest for data with complete coverage, suggesting that our sources do not selectively report father–son links.

30 The difference is smallest when the sample is restricted to sources with complete coverage ($\beta = 0.59$ vs. 0.48 parent–child elasticity). This is because the wedge between these two measures is partly explained by measurement error, which is smallest under complete coverage sources (see Appendix Eq. (16)).

31 Note that we show our targeted moments (median, Q75, and Q95) but also percentile moments that are not used in the estimation. Hence, the figure is in the spirit of an overidentifying restrictions test.

32 This relaxes the stationarity assumption in Eqs. (6) and (7). We now assume that the human capital of fathers and sons is drawn from the same distribution within a period, but we allow the distribution to change across periods. In addition, these estimates account for the possibility that the selection process to enter academia changed over time. This is because we allow all our model parameters to change across historical periods, including the parameters determining the “nepotic” entry threshold $\tau -\nu$.

33 All QQ plots for these differences are shown in Appendix Figs. E.3 to E.7.

34 Lenski (p. 176) argued that “[i]n the centuries before the Reformation, southern Europe was a centre of learning and intellectual inquiry [...] The Protestant Reformation ... gave a big boost to literacy, spawned dissents and heresies, and promoted the scepticism and refusal of authority that is at the heart of the scientific endeavour. The Catholic countries, instead of meeting the challenge, responded by closure and censure.”

35 Some faculties of arts, however, missed on fields such as cartography and astronomy. This led scientists like Copernicus, Kepler, or Galileo to quit their universities (Pedersen, 1996).

36 We omit other fields belonging to the faculty of arts, e.g., Hebrew, Philosophy, and Rhetoric.

37 For fathers and sons in multiple fields, we consider them in the same field if at least one field coincided.

38 As above, we exclude years before 1527 because the catholic-protestant distinction is not relevant before that date.

39 Before, $\nu$ captured how much sons are favored relative to first-generation scholars in the set of families.

40 This extended model also assumes the stationarity conditions (6) and (7). Hence, $\beta$ can still be characterized by in terms of mean reversion in the human capital distribution of potential scholars (Eq. (8)).

41 As before, we fix $\tau =0$ and recover $\mu _u$ and $\sigma _u$ from the stationarity conditions.

42 Although $\nu$ is redundant to $\gamma$, we report its value to further compare the two estimation approaches.

43 In terms of magnitude, this estimate for the father–son elasticity of publications in academia is similar to Braun and Stuhler (2018)’s population estimates for the elasticity of education attainment in Germany.

44 A means t-test rejects the null that our model’s $\beta$ is the same as the estimates $\hat{b}$, $\hat{b}_I$, $\rho _{PR}$, and $\rho _{PR,I}$.

45 This is akin to our Eqs. (4) and (5) but ignoring measurement error on the extensive margin, i.e., $\kappa =0$.

46 The number of observations is larger than the number of families because some families consist of more than three generations and some families contain brothers.

47 Note that this would generate father–son distributional differences in publications at the top of the distribution. Instead, we identify nepotism mainly from differences at the bottom of the distribution.

48 Note that unique publications may reflect nepotism in the publishing industry in the past, as this measure does not distinguish between work that is highly or scantly reproduced in modern libraries. The fact that results using both measures are similar rules out that the decline in $\gamma$ simply reflects an increased ability of sons to leverage their fathers’ nepotic connections to obtain more publications (in addition to obtaining academic positions).
