# Supplementary material for: Nepotism vs. intergenerational transmission of human capital in Academia (1088–1800)
Source: J Econ Growth (Boston). 2024 Jun 29;29(4):469–514. doi: 10.1007/s10887-024-09244-0 (PMC11502639; doi:10.1007/s10887-024-09244-0)
Supplement: Supplementary file 1 — (pdf 13503 KB) [file 10887_2024_9244_MOESM1_ESM.pdf]

Online Appendix to "Nepotism vs. Intergenerational Transmission of Human Capital in Academia (1088–1800)"

David de la Croix[*] Marc Goñi[†]

May 31, 2024

[*]IRES/LIDAM, UCLouvain & CEPR, Paris. E-mail: david.delacroix@uclouvain.be.
[†]Department of Economics, University of Bergen & CEPR, Paris. Email: marc.goni@uib.no

A Data appendix

This appendix lists the 343 most important secondary sources used to construct our dataset of 1,621 and 1,837 in universities and academies between 1088 and 1880. First, we complement the description in the main text on the coverage and accuracy of the data by providing additional summary statistics. Next, we describe in detail the secondary sources used for the largest universities and academies in our dataset and list all the data sources used for each institution in our dataset. Finally, we presents two examples: one to illustrate multiple-generation lineages of scholars (the Chicoyneau and Mögling dynasties), another to illustrate our data collection process (Honoré Bicais and his son Michel).

A.1 Additional descriptives on data coverage

As explained in Section 2 of the main text, we distinguish three levels of completeness in the sources used to construct our dataset of father-son pairs in pre-industrial academia (1088–1880):

- A *partial* coverage describes a situation in which the sample of scholars in an institution was informed by sources from other universities and general thematic biographies only. Under partial coverage, there is risk of sampling bias: On the one hand, if a scholar had a father who was briefly or no great account a professor, this is more likely to fall by the wayside than an underachieving son of a famous professor. On the other hand, if a scholar had a son who was briefly or no great account a professor, this is more likely to fall by the wayside than an underachieving father of a famous professor.
- A *broad* coverage is for father-son pairs in institutions where a members' catalogue listing all scholars in that institution does not exist. Instead, we use a combination of sources covering that particular institution, for example, a book on the history of that particular university or academy. Sources with broad coverage cover a large sample of scholars in an institution. Under broad coverage, hence, sampling bias is less likely, although we cannot fully rule it out.
- A *complete* coverage is for father-son pairs in institutions that are covered by an existing catalogue, compendium, website, or book whose aim is to list all scholars in that institution. For example, a source with complete coverage is a catalogue of all professors in a particular university or academy. Under complete coverage it is possible to distinguish whether a scholar's father was a professor or not with certainty.

Table A.1 shows the number of institutions and of father-son pairs by each coverage category. Around two thirds of our father-son pairs are from sources with complete coverage, 95.9% from sources with complete and broad coverage, and only 4.1% from sources with partial coverage. At the institution level, half of the universities and academies in our dataset have secondary sources with complete coverage, and 86 percent have secondary sources with complete and broad coverage. Importantly, the quality of the coverage is not related to the prestige of the university. We have an excellent

coverage of the University of Macerata—a small university in Italy, while there is no comprehensive list of professors for the University of Paris.

TABLE A.1: Breadth of coverage

Coverage	Number of institutions	Number of sons
Complete	90	1,178
Broad	64	585
Partial	25	74
Total	179	1,837

Next, we show that the share of father-son pairs coded from better sources is not heterogeneous across time, space, field of study, and religion. Specifically, Panel A of Figure A.1 shows the percentage of father-son pairs under complete and broad coverage by the country where the university or academy is located. Countries are based on modern borders. There is very little variation in this percentage, which ranges from ca. 90 to 100%. Note also that the countries where the percentage of father-son pairs from complete and broad sources is below 100 percent are both from north-west (e.g., UK) and southern Europe (e.g., Italy), and include both catholic and protestant countries.

In the main text, Table 1 showed that the coverage of the sources used to identify father-son pairs was stable across the four historical periods in our analysis: the period before 1543, the beginning of the Scientific Revolution (1543-1632), the second part of the Scientific Revolution (1633-1687), and the Enlightenment (1688-1800). Panel B of Figure A.1 complements this evidence by showing that the share of father-son pairs identified from better sources, by century. Specifically, it sorts father-son pairs by centuries based on the fathers' reference date—which includes a combination of their birth year, nomination year, or approximate activity year. The figure shows that the percentage of father-son pairs under complete and broad coverage is always above 90 percent, independenty of the century.

Similarly, Panel D of Figure A.1 shows that fathers and sons in the main fields of study that we consider in our analysis—theology, law, medicine (physicians), and science—are recovered from data sources of similar quality. As before, the percentage of fathers and sons under sources with complete and broad coverage varies little across their respective fields of study.

Finally, Panel E of Figure A.1 shows the breadth of the coverage by religion. We consider the religion of Universities after 1527—when the first Protestant University was established in Marburg. In both catholic and protestant universities, 95-100 percent of father-son pairs are based on sources with complete and broad coverage. We obtain a very similar result when we exclude theology scholars, who were typically priests or pastors and, hence, could only have (legitimate) descendants in protestant universities.

Altogether, this evidence shows that our main results, our results over time, and or heterogeneity analysis are based on sources with very good coverage, where the possibility of selective reporting of father-son links is unlikely. In other words, it is unlikely that our estimates are driven by sampling bias

Figure A.1: Percent of father-son pairs recovered from sources with complete and broad coverage

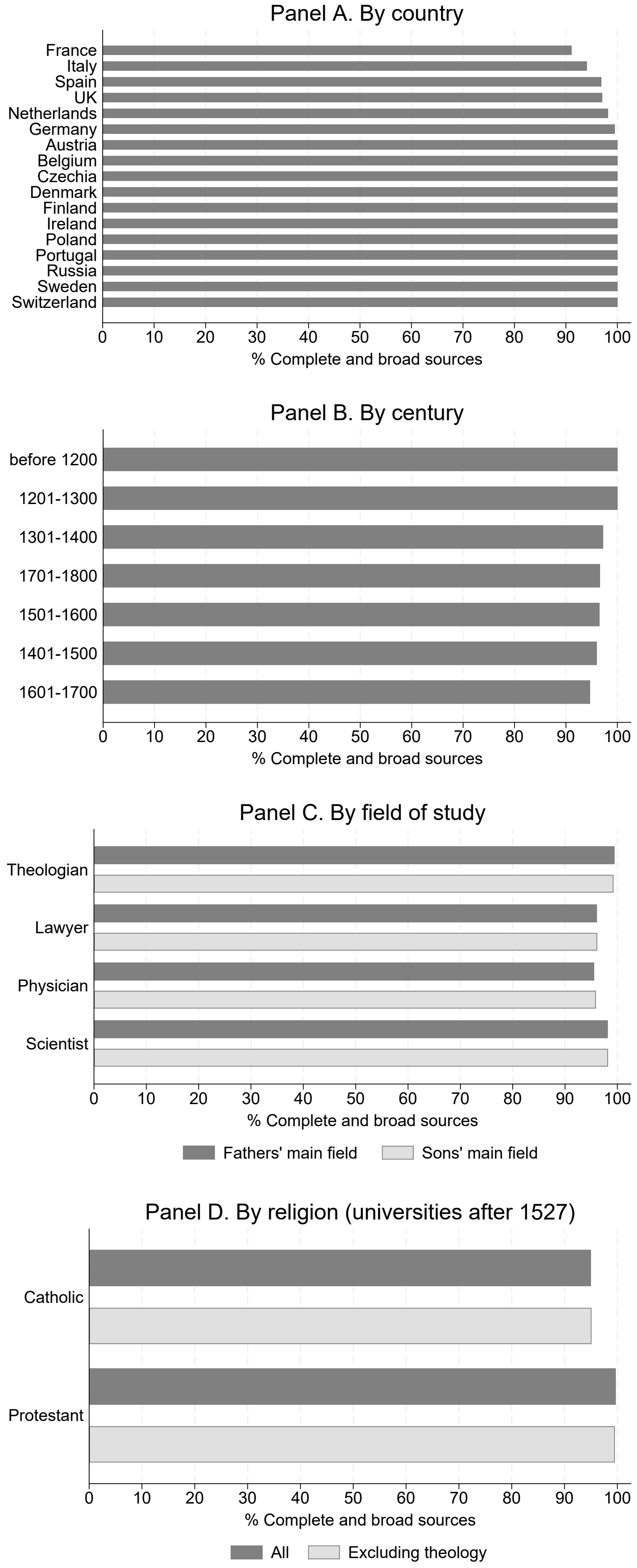

in the father-son links, or by composition effects where the groups compared are based on data sources with different coverage and accuracy. Nevertheless, to fully rule out the possibility of sampling bias, in the main text we examine the robustness of our results to using data with complete coverage alone.

A.2 Data sources

Table A.2 summarizes the ten institutions with more father-son pairs in our dataset. Table A.3 lists the secondary sources used for each of the 116 universities and 63 scientific academies included in our database. Specifically, the table provides the name of the university or academy, its foundation date (and, when applicable, closure date), the number of father-son pairs in that institution, the references for the secondary sources used, the coverage of these sources (3 =Complete, 2 =Broad, 1 =Partial), and the reference to the issue of the *Repertorium Eruditorum Totius Europae* if it exists.

TABLE A.2: Institutions with the largest number of father-son pairs

Institution (dates)	N	Main Sources	Biographical dictionary†
U. Bologna (1088-)	171	Mazzetti (1847)	Treccani
Royal Society (1660-)	78	www.royalsociety.org/	DNB
Accad. dei Ricovrati (1599-)	61	Maggiolo (1983)	Treccani
U. Padova (1222-)	60	Facciolati (1757),<br>Del Negro (2015)	Treccani
U. Avignon (1303-1793)	58	Laval (1889), Fournier (1892)<br>de Teule (1887), Duhamel (1895)	Barjavel (1841)
U. Cambridge	48	Walker (1927), Venn (1922)	DNB
U. Tübingen (1476-)	48	Conrad (1960)	ADB
U. Copenhagen (1475-)	47	Slottved (1978)	www.geni.com
U. Basel (1460-)	45	Herzog (1780)	Attinger (1928)
Leopoldina (1652-)	44	www.leopoldina.org/	ADB

Notes: ADB: Allgemeine Deutsche Biographie; DNB: Dictionary of National Biography; Treccani: Enciclopedia italiana; N: number of father-son pairs; †Main biographic dictionary used.

Table A.3: Sources used, number of father-son pairs, and coverage, by institution (1/7)

Institution	City	Country	Dates		Nb.	Sources	Cov.	RETE
University of Bologna	Bologna	ITA	1088		171	Mazzetti (1847)	3	1:1–10
Royal Society of London (···)	London	GBR	1660		78	https://royalsociety.org/	3	7:11–19
Accademia dei Ricovrati	Padova	ITA	1599		61	Maggiolo (1983)	3	51–63
University of Padua	Padova	ITA	1222		60	Pesenti (1984), Casellato and Rea (2002), Facciolati (1757),Del Negro (2015)	3	3:33–42
University of Avignon	Avignon	FRA	1303	1793	58	Laval (1889), Fournier (1892), de Teule (1887), Duhamel (1895), Barjavel (1841)	2	
University of Tubingen	Tübingen	DEU	1476		48	Conrad (1960)	3	7:21–30
University of Cambridge	Cambridge	GBR	1209		48	Walker (1927), Venn (1922)	2	
University of Copenhagen	København	DNK	1475		47	Slottved (1978)	3	2:21–29
University of Basel	Basel	CHE	1460		45	Herzog (1780), Junius Institute (2013) Rosen (1972)	2	
Academy of Sciences Leopoldina	Halle	DEU	1652		44	http://www.leopoldina.org/	3	
University of Montpellier	Montpellier	FRA	1289	1793	37	Astruc (1767), Dulieu (1975, 1979, 1983)	2	
University of Leipzig	Leipzig	DEU	1409		35	von Hehl and Riechert (2017),Schwinges and Hesse (2019)	3	8:33–42
University of Jena	Jena	DEU	1558		30	Günther (1858)	3	1:25–32
Univ. of Pavia	Pavia	ITA	1361		29	Raggi (1879)	3	8:45–52
University of Königsberg	Kaliningrad	RUS	1544		27	Naragon (2006), Schwinges and Hesse (2019)	2	
University of Marburg	Marburg	DEU	1527		25	Gundlach and Auerbach (1927)	3	
University of Greifswald	Greifswald	DEU	1456		24	https://www.uni-greifswald.de	2	
University of Giessen	Gießen	DEU	1607		24	Haupt and Lehnert (1907)	3	2:31–38
University of Helmstedt	Helmstedt	DEU	1575	1809	22	Gleixner (2019)	3	
Royal Prussian Acad. of Sciences	Berlin	DEU	1700		22	BBAW (2022)	3	3:1–9
French Academy of Sciences	Paris	FRA	1666	1793	22	Maury (1864), Rozier (1776)	3	9:1–10
University of Strasbourg	Strasbourg	FRA	1538		22	Berger-Levrault (1890)	3	8:7–15
University of Paris	Paris	FRA	1200	1793	21	Antonetti (2013), Courtenay (1999), Hazon and Bertrand (1778)	2	
University of Rostock	Rostock	DEU	1419		19	Krüger (2019)	3	
University of Wittenberg	Wittenberg	DEU	1502	1813	17	Kohnle and Kusche (2016)	2	10:47–55

Table A.3: Sources used, number of father-son pairs, and coverage, by institution (2/7)

Institution	City	Country	Dates		Nb.	Sources	Coverage	RETE
University of Leiden	Leiden	NLD	1575		17	Leiden (2019)	3	
University of Lund	Lund	SWE	1666		17	Tersmeden (2015),Delen and Weibull (1868)	3	5:9–16
University of Perugia	Perugia	ITA	1308		16	Frova and Zucchini (2017),Zucchini (2008)	2	
University of Edinburgh	Edinburgh	GBR	1582		15	Junius Institute (2013), Grant (1884)	3	
University of Geneva	Genève	CHE	1559		14	Junius Institute (2013), Borgeaud (1900)	3	1:41–47
Académie royale d'architecture	Paris	FRA	1671	1793	14	www.cths.fr	1	
Académie Royale (· · ·) de Lyon	Lyon	FRA	1700	1790	13	Dominique (2017)	3	
Collège Royal	Paris	FRA	1530		13	Collège de France (2018)	3	1:19–24
University of Cahors	Cahors	FRA	1332	1751	10	Ferté (1975)	2	
University of Halle	Halle (Saale)	DEU	1694	1817	13	Schopferer (2016)	3	
University of Pisa	Pisa	ITA	1343		13	Fabroni (1791)	3	11:25–33
University of Salamanca	Salamanca	ESP	1218		13	Addy (1966),Arteaga (1917), Vidal y Díaz et al. (1869)	3	7:1–9
University of Louvain	Leuven	BEL	1425	1797	12	Ram (1861), Nève (1856),Brants (1906), Lamberts and Roegiers (1990)	2	4:53–66
University of Heidelberg	Heidelberg	DEU	1386		12	Drüll (1991), Drüll (2002)	3	6:25–34
Academy of(· · ·) Mainz	Erfurt	DEU	1754		12	Kiefer (2004)	3	
Royal Swedish Academy of Sc.	Stockholm	SWE	1739		12	http://www.kva.se, Dahlgren (1915)	3	
University of Valence	Valence	FRA	1452	1793	12	Brun-Durand (1900), Nadal (1861)	2	2:13–20
University of Aix	Aix-en-Provence	FRA	1409	1793	11	Belin(1905), Fleury and Dumas (1929), De la Croix and Fabre (2019)	2	4:35–44
Accademia Fiorentina	Firenze	ITA	1540	1783	11	Boutier (2017)	1	
University of Oxford	Oxford	GBR	1200		11	Emden (1959), Foster (1891)	2	
University of Franeker	Franeker	NLD	1585	1811	11	Feenstra, Ahsmann, and Veen (2003)	2	
University of Kiel	Kiel	DEU	1652		11	Volbehr and Weyl (1956)	3	
Collegium Carolinum	Zurich	CHE	1525		11	Junius Institute (2013), Attinger, Godet, and Türler (1928)	2	
University of Poitiers	Poitiers	FRA	1431	1793	10	Boissonade (1932)	2	
Uppsala University	Uppsala	SWE	1477		10	Von Bahr (1945), Astro.uu.se (2011), Jensen (2018)	2	

Table A.3: Sources used, number of father-son pairs, and coverage, by institution (3/7)

Institution	City	Country	Dates		Nb.	Sources	Coverage	RETE
Royal Society of Edinburgh	Edinburgh	GBR	1783		10	Waterston and Shearer (2006)	3	10:1–7
University of Toulouse	Toulouse	FRA	1229	1793	10	Deloume (1890), Barbot (1905), Lamothe-Langon (1823)	2	8:53–63
Académie des inscriptions (· · ·)	Paris	FRA	1663		10	Boutier (2018)	2	
University of Bordeaux	Bordeaux	FRA	1441	1793	10	Gaullieur (1874),Pery (1888)	2	
University of Lausanne	Lausanne	CHE	1537		10	Junius Institute (2013),Kiener and Robert (2005)	3	
University of Salerno	Salerno	ITA	1231		9	Sinno (1921), De Renzi (1857)	2	
University of Torino	Torino	ITA	1404		9	Vallauri (1875)	2	7:31–38
University of Glasgow	Glasgow	GBR	1451		5	Coutts (1909), University of Glasgow (2020)	2	
Zamojski University	Zamosc	POL	1594	1784	9	Kedzoria (2021)	2	10:31–37
University of Freiburg	Freiburg	DEU	1457		9	Bauer (1957)	2	9:59–68
Societas Privatas Taurinensis	Torino	ITA	1757	1792	8	Accademia delle Scienze di Torino (1973)	3	5:43–51
University of Göttingen	Göttingen	DEU	1734		8	Ebel (1962)	3	4:1–8
University of Angers	Angers	FRA	1250	1793	8	Rangeard and Lemarchand (1868), De Lens (1880), Denéchère and Matz (2012), Port (1876)	2	
Jagiellonian University	Krakow	POL	1364		8	Pietrzyk and Marcinek (2000), http://www.archiwum.uj.edu.pl/	3	6:35–42
Accademia della Crusca	Firenze	ITA	1583		7	Parodi (1983)	3	10:39–45
Utrecht University	Utrecht	NLD	1636		7	Dorsman (2011)	3	
Jardin Royal des Plantes	Paris	FRA	1635	1793	7	Jaussaud and Brygoo (2004)	3	
Academy of St Petersburg	Saint-Petersburg	RUS	1724	1917	7	Shemivot (1873)	3	5:17–25
University of Siena	Siena	ITA	1246		7	Frova, Catoni, and Renzi (2001)	2	
French Academy	Paris	FRA	1635		7	http://www.academie-francaise.fr/	3	6:17–23
University of Groningen	Groningen	NLD	1612		7	https://hoogleraren.ub.rug.nl/	3	
Åbo Akademi University	Turku	FIN	1640		7	Klinge et al. (1988)	2	
Académie d'agriculture	Paris	FRA	1761	1793	7	Marion (2019)	3	
University of Pont-à-Mousson	Pont-à-Mousson	FRA	1572	1768	7	Martin (1891)	3	2:1–6
Dutch Academy of Sciences	Haarlem	NLD	1752	1804	7	https://khmw.nl/historische-leden/	3	
University of Florence	Firenze	ITA	1321	1515	7	Prezziner (1810), Gherardi (1881)	2	

Table A.3: Sources used, number of father-son pairs, and coverage, by institution (4/7)

Institution	City	Country	Dates		Nb.	Sources	Coverage	RETE
Académie des Sciences et B.-L.	Toulouse	FRA	1729	1793	7	Taillefer (1985)	3	6:51–58
University of Dole	Dole	FRA	1422	1793	7	Beaune and d'Arbaumont (1870)	2	6:43–50
Accademia delle scienze	Bologna	ITA	1714		7	Ercolani (1881)	3	7:57–65
Royal Spanish Academy	Madrid	ESP	1713		6	https://www.rae.es/la-institucion/	3	3:11–17
University of Perpignan	Perpignan	FRA	1350	1793	6	Carmignani (2017), Capeille (1914),Izarn (1991)	2	
Bavarian Academy of (· · ·)	München	DEU	1759		6	Bayerische Akademie der Wissenschaften (2022)	3	
University of Nantes	Nantes	FRA	1460	1793	6	Chenon (1890), Grünblatt (1961)	2	
"Mersenne" Academy	Paris	FRA	1635	1648	6	de Coste (1648)	3	2:7–12
University of Dublin	Dublin	IRL	1592		6	Kirkpatrick (1912), Burtchaell and Sadleir (1935)	2	
University of Caen	Caen	FRA	1432	1793	6	de Pontville (1997)	1	
University of Ingolstadt	Ingolstadt	DEU	1459	1800	6	Wolff (1973)	3	
Académie des Sciences et B.-L.	Bordeaux	FRA	1712	1793	6	Courteault (1912)	3	4:9–17
Académie des Sciences et B.-L.	Dijon	FRA	1725	1793	6	Milsand (1871)	3	11:17–24
Old University of Aberdeen	Aberdeen	GBR	1495		6	Anderson (1893)	3	4:27–34
University of Altdorf	Altdorf bei Nürnberg	DEU	1578	1809	5	Flessa (1969)	2	
Royal College of Physicians	London	GBR	1518		5	Munk (1878)	1	
University of Modena	Modena	ITA	1175		5	Mor and di Pietro (1973)	2	9:25–32
University of Valladolid	Valladolid	ESP	1280		5	Alcocer Martinéz (1918)	3	1:11–18
University of Ferrara	Ferrara	ITA	1391		5		1	
New University of Aberdeen	Aberdeen	GBR	1593		5	Anderson (1898)	3	
Majorcan cartographic school	Palma	ESP	1330	1500	5	Pastor and Camarero (1960)	2	
Royal Dublin Society	Dublin	IRL	1757		5	Berry (1915)	3	6:9–15
University of Rinteln	Rinteln	DEU	1620	1809	4	Hänsel (1971)	3	
University of Mainz	Mainz	DEU	1476	1792	4	Benzing (1986)	3	
Académie des Arts et B.-L.	Caen	FRA	1705	1793	4	de Pontville (1997)	3	
University of St Andrews	Saint-Andrews	GBR	1411		4	Smart (2004)	2	
Académie (.) de la Rochelle	La Rochelle	FRA	1732	1744	4	Marion (2019)	2	
Universty of Naples	Napoli	ITA	1224		4	Origlia Paolino (1754)	2	
University of Montauban	Montauban	FRA	1598	1659	4	Bourchenin (1882)	3	

TABLE A.3: Sources used, number of father-son pairs, and coverage, by institution (5/7)

Institution	City	Country	Dates		Nb.	Sources	Cov.	RETE
University of Sedan	Sedan	FRA	1599	1681	4	Bourchenin (1882)	3	
Akademisches Gymnasium Danzig	Gdansk	POL	1558		4	Hirsch (1837)	3	
Société Royale des Sciences	Montpellier	FRA	1706	1793	3	Dulieu (1983)	3	1:33–39
University of Harderwijk	Harderwijk	NLD	1647	1811	3	van Epen (1904)	3	
Braunschweig University (· · ·)	Braunschweig	DEU	1745		3	Albrecht and Gundler (1986)	3	
University of Orléans	Orléans	FRA	1235	1793	3	Bimbenet (1853), Duijnstee (2010)	2	
University of Rome	Roma	ITA	1303		3	Renazzi (1803)	2	
Gottingen Academy of Sciences	Göttingen	DEU	1752		3	Krahnke (2001)	3	
University of Catania	Catania	ITA	1444		3	Sabbadini (1898)	2	
University of Douai	Douai	FRA	1559	1793	3	Collinet (1900)	2	
Académie des Sciences, ...	Clermont-Ferrand	FRA	1759	1793	3	Mège (1999)	3	8:27–32
Academy of Spalding	Spalding	GBR	1710	1770	3	Matthew, Harrison, and Long (2004)	2	
University of Saumur	Saumur	FRA	1596	1685	2	Bourchenin (1882)	3	
Académie des belles-lettres, (· · ·)	Marseille	FRA	1726	1793	2	http://www.academie-sla-marseille.fr/	2	
University of Würzburg	Würzburg	DEU	1402		2	Walter (2010)	2	
Viadrina European University	Frankfurt O.	DEU	1506	1811	2	Junius Institute (2013),Schwinges and Hesse (2019)	2	
Universite of Die	Die	FRA	1601	1684	2	Bourchenin (1882)	3	
University of Macerata	Macerata	ITA	1540		2	Serangeli (2010)	3	2:39–45
Academy of Gorlitz	Gorlitz	DEU	1773		2	https://www.olgdw.de/gesellschaft/	3	
Agriculture Society of Lyon	Lyon	FRA	1761		2	Marion (2019)	1	
University of Erlangen	Erlangen	DEU	1742		2	Wachter (2009)	3	
Danzig Research Society	Gdansk	POL	1743	1936	2	Schumann (1892)	3	1:49–54
University of Vienna	Wien	AUT	1465		2	Lackner (1976), Schwinges and Hesse (2019), von Aschbach (1865)	2	
Literary and philosophical society	Manchester	GBR	1781		2	Anonymous (1896)	3	
University of Parma	Parma	ITA	1412		2	Rizzi (1953)	2	6:1–8
Accademia Aldina	Venezia	ITA	1494	1515	2	Da-Rio (1828)	2	
University of Coimbra	Coimbra	PRT	1308		2	Rodrigues (2003),Rodrigues (1992)	3	9:49–57
Derby Philosophical Society	Derby	GBR	1783	1858	2	Sturges (1978)	3	

Table A.3: Sources used, number of father-son pairs, and coverage, by institution (6/7)

Institution	City	Country	Dates		Nb.	Sources	Coverage
Academy of Castres	Lyon	FRA	1648	1670	2	Marion (2019)	1
Academy of the Unknown	Venezia	ITA	1626	1661	1	The British Library (2021)	1
Athenaeum Illustre of Amsterdam	Amsterdam	NLD	1632	1877	1	http://www.albumacademicum.uva.nl/	3
Academy of the Burning Ones	Padova	ITA	1540	1545	1	The British Library (2021)	1
Freiberg University (· · ·)	Freiberg	DEU	1765		1	Appelt and Wulkow (2022)	3
Nijmegen University	Nijmegen	NLD	1655	1679	1		1
Veneziana (Seconda Accademia)	Venezia	ITA	1594	1608	1	The British Library (2021)	2
University of Nîmes	Nîmes	FRA	1539	1663	1	Bourchenin (1882)	3
University of Moscow	Moskow	RUS	1755		1	Andreev and Tsygankov (2010)	3
Academy of the Invaghiti	Mantova	ITA	1562	1738	1	The British Library (2021)	2
University of Rennes	Rennes	FRA	1735	1793	1	Chenon (1890)	3
University of Prague	Prague	CZE	1348		1	Svatoš and Čornejová (1995), Čornejová and Fechtnerová (1986)	2
University of Erfurt	Erfurt	DEU	1379		1	Schwinges and Hesse (2019)	1
Royal Botanic Garden	Kew	GBR	1759		1		1
Academie de Beziers	Béziers	FRA	1723	1793	1	Marion (2019)	1
University of Cervera	Cervera	ESP	1714	1821	1	Rubio y Borras (1914)	3
Academy of the Umorists	Roma	ITA	1603	1670	1	The British Library (2021)	2
Academy of the Filateri	Ferrara	ITA	1554	1563	1	The British Library (2021)	2
University of Bourges	Bourges	FRA	1464	1793	1		2
University of Orange	Orange	FRA	1365		1		1
Society of Observers of Man	Paris	FRA	1799	1804	1		1
University of Oviedo	Oviedo	ESP	1574		1	Canella Secades (1873)	1
Royal Danish Science Society	Copenhagen	DNK	1742		1	Lomholt (1950)	3
French Academy of Medecine	Paris	FRA	1731	1793	1		1
University of Duisburg	Duisbrug	DEU	1654		1		1
University of Cagliari	Cagliari	ITA	1606		1	Tola (1837), Pillosu (2017)	2
Accademia Roveretana	Rovereto	ITA	1752		2		1
University of Alcala	Alcala de H.	ESP	1499		1	Torrecilla, Arboniés, and Torres (2013)	2

Table A.3: Sources used, number of father-son pairs, and coverage, by institution (7/7)

Institution	City	Country	Dates		Nb.	Sources	Coverage	RETE
University of St Petersburg	St. Petersburg	RUS	1724		1	Shemivot (1873)	2	
Philosophical Society	Dublin	IRL	1683	1778	1	Wilde and Lloyd (1844)	1	
Accademia Botanica	Firenze	ITA	1739	1783	1		1	
University of Dijon	Dijon	FRA	1722	1792	1		1	
University of Fermo	Fermo	ITA	1585		1	Brizzi (2001), Curi (1880)	2	5:27–34
University of Pau	Pau	FRA	1722	1793	1	Maisonnier (1972)	1	
University of Besancon	Besancon	FRA	1691	1793	1	Lavillat (1977)	3	
University of Nancy	Nancy	FRA	1768	1793	1		1	
Académie des sciences, ...	Angers	FRA	1685	1793	1	Marion (2019)	2	
University of Mondovi	Mondovi	ITA	1560		1	Vallauri (1875)	3	
Académie des Sciences, ...	Besancon	FRA	1709	1709	1	Defrasne et al. (2002)	3	7:39–45
Scuole Palatine	Milano	ITA	1647	1647	1		1	
Oxford Philosophical Society	Oxford	GBR	1658	1658	1	Gunther (1925)	2	8:17–25
Accademia Boreliana	Palmi	ITA	1673	1673	1	Maylender (1930)	1	
Société des Sciences,...	Agen	FRA	1778	1778	1	Labande (1901)	3	
Académie des belles lettres	Montauban	FRA	1738	1738	1	Forestié (1888)	3	
Accademia Filopatria	Torino	ITA	1759	1759	1	Calcaterra (1943)	3	
Société des sciences physiques	Lausanne	CHE	1785	1785	1	Société de Lausanne (1790)	3	
Societatis Regiae Scientiarum Upsaliensis	Uppsala	SWE	1700	1700	1	Karlberg (1977)	2	

Notes: Missing sources correspond to families which were mentioned in sources about other institutions.
Coverage: 3=Complete, 2=Broad, 1=Partial.

A.3 Examples

Multi-generation lineages of scholars. Our database contains 176 families with three or more generations of scholars at the same university or scientific academy. For the sake of illustration, Figure A.2 shows one of these dynasties of scholars: the Chicoyneau. The Chicoyneaus had four generations of scholars, all employed at the University of Montpellier. For almost a century (from 1659 to 1758), there was at least one Chicoyneau at the University of Montpellier. This lineage was reconstructed using Dulieu (1983). Note that some Chicoyneaus developed a prolific career. For example, François Chicoyneau (1672-1752) was a professor at Montpellier and was also appointed at the Académie des Sciences. Other members of the dynasty were appointed professor at very early ages. The last member of the dynasty, Jean-François Chicoyneau (born in 1737), was made a professor in 1752—that is, at the tender at age of 15. In principle, dynasties like the Chicoyneaus may emerge because human capital was strongly transmitted across generations, because of nepotism, or because of a combination of both.

Similarly, Figure A.3 displays another multi-generation lineage of scholars: the Mögling family at the University of Tübingen (Conrad 1960). This lineage spans six generations, from the sixteenth to the eighteenth century. The first three generations were professors in medicine. After Johan David Mögling (1650-1695), however, the family switched to law (in Section 6.1 of the main text, we exploit such field switches). In the first and fifth generation, the lineage members held a professorship elsewhere: Daniel Mögling (1546-1603) at Heidelberg, Johan Friedrich Mögling (1690-1766) at Giessen.

In the main text, we exploit these multi-generation lineages to address measurement error in estimates for the transmission of human capital. Specifically, we use multi-generation lineages to compute correlations in observed publications across multiple generations. Elsewhere it has been shown that, under the assumption that measurement error is constant across generations, these multi-generation correlations reflect the transmission of (unobserved) underlying human-capital endowments. In other words, multi-generation lineages help us tackle the measurement error bias in parent-child publication elasticities.

Data collection example - Honoré and Michel Bicais. In Section 2 on the main text, we illustrate the data collection process by using the example of Honoré Bicais and his son Michel, both professors at the University of Aix. Figure A.4 shows the different sources mentioned in the main text: **(a)** Honoré Bicais' biography from Belin's *Histoire de l'Ancienne Universite de Provence* (1905) — used to identify Honoré (and Michel) as professors at the University of Aix; **(b)** The biographical dictionary of Aix's Department, *Les Bouches-du-Rhône, Encyclopédie Départementale* by (Mason 1931) — used to retrieve birth years and the quote that Michel Bicais succeeded his father in "in his chair and in his reputation;" and **(c)** Honoré and Michel Bicais' WorldCat entries — used to measure their scientific output in the form of library holdings by or about them in modern libraries.[1]

[1] The WorldCat entries in Figure A.4 were accessed on 30th of November, 2020. The number of library holdings may change slightly if modern libraries acquire/retire copies of the works by or about these authors.

Figure A.2: The Chicoyneau dynasty

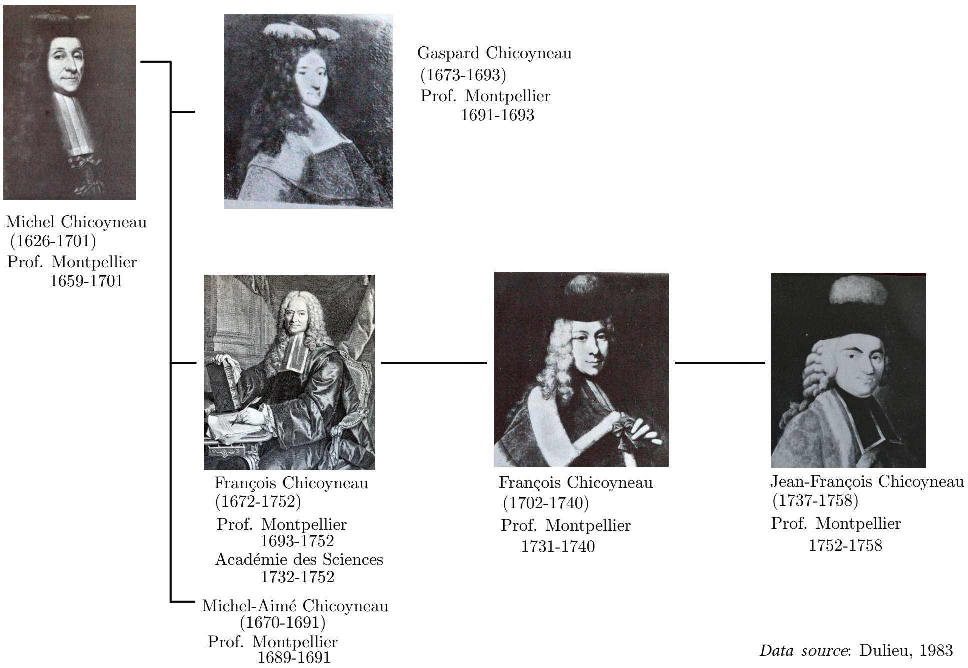

Data source: Dulieu, 1983

Figure A.3: The Mögling dynasty

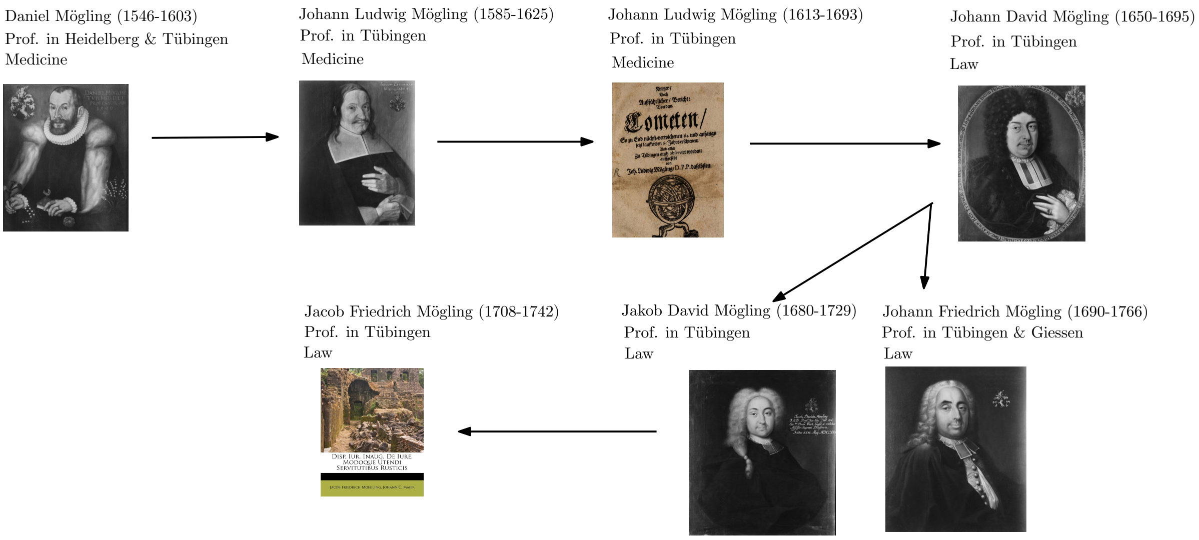

Figure A.4: Example of data collection - Honoré and Michel Bicais

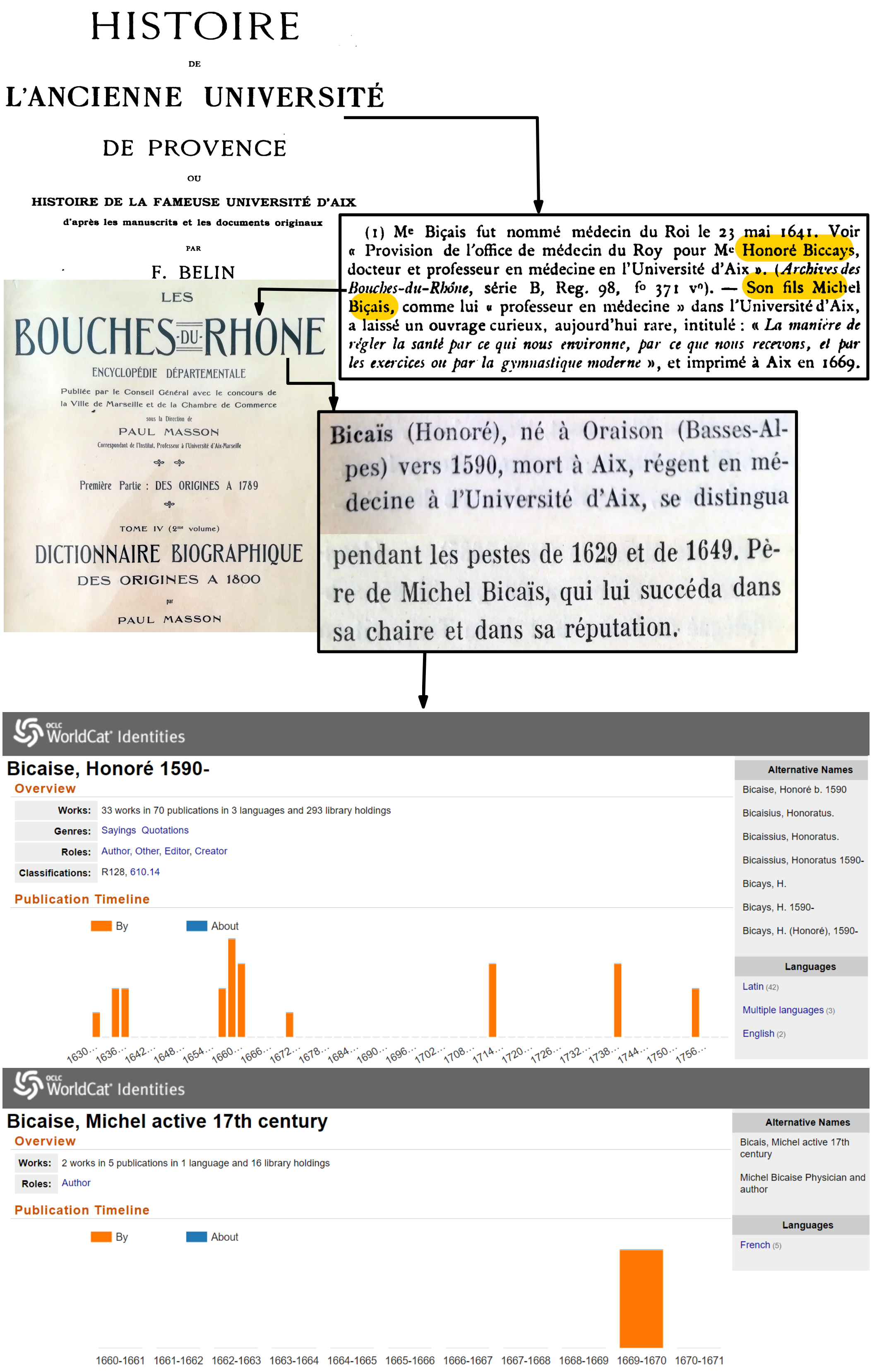

B Intergenerational estimates in the literature

This appendix describes existing methods in the literature to estimate intergenerational elasticities, and highlights two potential biases: measurement error and selection of families in the data.

Parent-child elasticities. To study the extent to which inequalities are transmitted across generations, economists typically estimate coefficient b in:

$$y_{i,t+1} = b\, y_{i,t} + e_{i,t+1}\,,$$

where i indexes families, t parents, and t+1 children. The outcome y reflects social status (e.g., income, wealth, education, occupation) and is in logarithms. The coefficient b is the intergenerational elasticity of outcome y. It determines the speed at which outcomes revert to the mean. To see this, note that the half-life of y (the generations until the gap to the mean halves) is $t_{\frac{1}{2}} = -\ln(2) \;/\; \ln(|b|)$, which depends negatively on b.

Panel A of Table B.1 shows estimates of b in the literature.[2] Parent-child elasticities vary across time and space, but are generally below 0.5. This implies a half-life of $t_{\frac{1}{2}} = 1$. That is, half the gap to the mean is filled after one generation. In three generations, almost all advantages will revert to the mean.

Measurement error bias. Recent studies looking at multiple generations show that social status is more persistent than suggested by parent-child elasticities. One possible reason is that there is a highly-persistent inherited endowment that wealth, income, or occupation only reflect noisily. Children do not inherit their socio-economic outcomes directly from their parents. Instead, children inherit an unobserved human capital endowment h (e.g., knowledge, skills, genes, preferences) which then transforms into the observed outcome y imperfectly. This is modeled as a first-order Markov process of endowments transmission where endowments are observed with measurement error (Clark and Cummins 2015; Braun and Stuhler 2018):

$$h_{i,t+1} = \beta h_{i,t} + u_{i,t+1}\,, \tag{1}$$

$$y_{i,t+1} = h_{i,t+1} + \varepsilon_{i,t+1}\,, \tag{2}$$

where $h_{i,t} \sim N(\mu_h, \sigma_h^2)$ and $u_{i,t+1}$ and $\varepsilon_{i,t+1}$ are independent noise terms. The coefficient β captures the extent to which the parents' endowment h is inherited by their children. In this sense, β is the parameter governing the true rate of persistence of social status across generations. In contrast, Equation (2) determines how well this endowment is reflected in the observed outcome y. A larger variance in the noise term, σ_ε^2, is associated with a lower observability of the endowment h.

The intergenerational elasticity of outcome y estimated from Equation (13) is:

$$E(\hat{b}) = \beta\, \frac{\sigma_h^2}{\sigma_h^2 + \sigma_\varepsilon^2} := \beta\, \theta, \tag{3}$$

[2] For a more thorough review, see Solon (1999), Corak (2006), and Black and Devereux (2011).

TABLE B.1: Persistence of social status in the literature

**Panel A**: Estimates of b		
$\hat{b}$	y_t	Country & Source
0.31–0.41	Wealth	Agricultural societies (Borgerhoff Mulder et al. 2009)
0.48–0.59	Wealth	UK (Harbury and Hitchins 1979)
0.225	Wealth	Norway (adoptees) (Fagereng, Mogstad, and Ronning)
0.6	Earnings	USA (Mazumder 2005)
0.34	Earnings	USA (Chetty et al. 2014)†
0.47	Earnings	USA (Corak 2006)
0.19–0.26	Earnings	Sweden (Jantti et al. 2006)
0.11–0.16	Earnings	Norway (Jantti et al. 2006)
0.46	Education	USA (Hertz et al. 2007)
0.71	Education	UK (Hertz et al. 2007)
0.35	Education	Sweden (Lindahl et al. 2015)
0.35	Body Mass	USA (Classen 2010)
**Panel B**: Estimates of β		
$\hat{\beta}$	y_t	Data & Source
0.70–0.75	Wealth	UK probate (1858–2012) (Clark and Cummins 2015)
0.70–0.90	Oxbridge	UK (1170–2012) (Clark and Cummins 2014)
0.61–0.65	Occupation	Germany, 3 gen. (Braun and Stuhler 2018)
0.49–0.70	Education	Germany, 4 gen. (Braun and Stuhler 2018)
0.6	Education	Spain, census (Güell, Rodríguez Mora, and Telmer 2015)
0.61	Schooling	Sweden, 4 gen. (Lindahl et al. 2015)
0.49	Earnings	Sweden, 4 gen. (Lindahl et al. 2015)
0.74	Education	EU-28, 3 gen. (Colagrossi, d'Hombres, and Schnepf 2019)
0.8	Education	Spain, census (Collado, Ortuno-Ortin, and Stuhler 2018)

† Rank-rank slope instead of log-log elasticity.

where $\theta < 1$ is an attenuation bias for β.

Several methods have been used to identify β. One is to exploit correlations in y across multiple generations.[3] According to the first-order Markov process described above, the elasticity of outcome y is $\beta\theta$ between parents, t, and children, $t+1$, and $\beta^2\theta$ between grandparents, t, and grandchildren, $t+2$ (as long as the signal-to-noise ratio is stable across generations). Hence, the ratio of these elasticities identifies β. Intuitively, β is identified because the endowment h is inherited, but the estimation bias θ is not—it is the same across two or three generations. Another identification strategy for β is to estimate intergenerational regressions of Equation (13)'s form with group-average data for siblings (Braun and Stuhler 2018) or for people sharing rare surnames (Clark and Cummins 2015). By grouping individuals with similar inherited endowments, the noise term ε is averaged away. Güell,

[3]Lindahl et al. (2015), Braun and Stuhler (2018), Colagrossi, d'Hombres, and Schnepf (2019).

Rodríguez Mora, and Telmer (2015) propose to identify β through the informational content of rare surnames (ICS)—a moment capturing how much individual surnames explain the total variance of individual outcomes.[4] This method only requires cross-sectional data, i.e., it does not require linking data across generations. Similarly, Collado, Ortuno-Ortin, and Stuhler (2018) estimate β using horizontal kinship correlations in the cross-section.

Panel B of Table B.1 reports estimates of β from these different approaches. The estimates range between 0.49 and 0.90, and hence are substantially larger than the parent-child elasticities b. Furthermore, Clark (2015)'s comprehensive evidence suggests that β is close to a "universal constant" across societies and historical periods. This finding is disputed by studies using the ICS (Güell et al. 2018) or multi-generation links (Lindahl et al. 2015; Braun and Stuhler 2018; Colagrossi, d'Hombres, and Schnepf 2019) instead of surname-averages.

In light of this evidence, the unobserved endowment that children inherit from their parents has often been interpreted as skills, preferences, or even genes. First, because these endowments reflect well the measurement error problem described here: wealth, income, education, etc. only reflect skills and innate abilities with noise. Second, because if β is a universal constant, it should reflect nature rather than nurture. In other words, if β does not vary substantially across time and space, an obvious conclusion is that institutions, social policies, or processes of structural economic transformation cannot affect social mobility in the long run.

We argue that these estimates may be subject to another source of bias in settings where favoritism or nepotism are prevalent. That is, where those with power and influence give preference to friends and relatives ahead of better-qualified outsiders. For example, estimates of occupational or wage persistence may be affected by the fact that certain jobs have higher entry barriers for outsiders than for sons of insiders. Econometrically, this introduces a different bias: selection.

Selection bias. Beyond measurement error, parent-child elasticities may be subject to sample selection: whether observations are sampled or not may be correlated with the unobserved endowment h inherited by children. This additional source of bias is inherent to data used to evaluate social mobility. It is present in applications that focus on a subgroup of the population, e.g., an occupation or those leaving wills. Specifically, in certain occupations relatives of insiders may be more likely to be observed. This kind of selection bias is typically addressed using natural experiments. Similarly, wealth elasticities rely on wills and probate records, where only those leaving wealth above a legal requirement are sampled (Clark and Cummins 2015). This sampling criterion is likely to be correlated with h, an individual's inherited endowments (e.g., social competence, skills, genes). Sample selection may also arise in applications covering the entire population. In census data linking several generations, families are not observed if a generation migrates or dies before outcomes are realized (e.g., wage, occupation choice). This attrition can be correlated with the underlying endowment h. Finally, life-history data collected retrospectively may suffer from recall bias. This bias depends on h if families with large endowments have better knowledge of their ancestors.

[4] The ICS is the difference in the R^2 of regressing y on a vector of dummies indicating surnames vs. a regression in which this vector indicates "fake" surnames.

To see how selection affects intergenerational elasticity estimates, let s be a selection indicator such that $s_i = 1$ if family i is used in the estimation, and $s_i = 0$ if it is not. The intergenerational elasticity of y estimated from Equation (13) is:

$$E(\hat{b}) = b + \frac{\text{Cov}\left(s_i y_{i,t},\ s_i e_{i,t+1}\right)}{\text{Var}\left(s_i y_{i,t}\right)}.$$

Note that if $\text{Cov}\left(s_i y_{i,t},\ s_i e_{i,t+1}\right) = 0$, then $\hat{b}$ is an unbiased estimate of b and a biased estimate of β due to measurement error, i.e., $\hat{b} = \theta\beta$. If the selection indicator, s_i, is correlated with the underlying endowments transmitted across generations, $h_{i,t}$ and $h_{i,t+1}$, then the condition above is violated and $\hat{b}$ is a biased estimate of b.

These two biases are fundamentally different. As described above, measurement error can be corrected using multiple generations. The reason is that across n generations, the underlying endowment is inherited $n-1$ times at a rate β but only twice transformed into the observed outcome y with measurement error. This is not true for the selection bias, which depends on the h, and hence is 'inherited' $n-1$ times. For example, consider grandparent-grandchild (and parent-child) correlations in outcomes: The correlations depend on β—which is inherited twice (once), on the measurement error with which h is twice (twice) transformed into y, and on the selection bias—which is also 'inherited' twice (once). Hence, the ratio of grandparent-grandchild to parent-child correlations does not correct for selection. Moreover, if selection changes over time (e.g., due to changes in the prevalence of nepotism) this bias may differ across two and three generations. In other words, the ratio of grandparent-grandchild to parent-child correlations may provide upward or downward biased estimates of β.[5]

Henceforth, we restrict our analysis to sample selection—the bias emerging when inherited human capital is correlated to whether families are sampled or not. Another issue is whether human capital endowments (h) are genetically inherited (selection) or are determined by parental investments (causation).[6] We abstract from this selection story as our main purpose is to disentangle nepotism from human capital endowments, regardless of whether the latter are determined by nature or nurture. That said, in our empirical application it is possible that a scholar strategically invests in the human capital of his most endowed son, i.e., the son with higher chances of becoming a scholar *ex ante*. Unfortunately, we only observe the children of scholars who become scholars themselves. Hence, we cannot use sibling comparisons to address this issue. That said, such strategic investments in the most endowed son would lead to understating the rate of mean reversion in scholars' human capital and to overstating nepotism—which we already estimate to be low in periods of rapid scientific advancement.

[5]Formally, this ratio is an upward biased estimate of β if $\frac{\text{Cov}\left(s_i\, y_{i,t},\ s_i\, e_{i,t+2}\right)}{\text{Cov}\left(s_i\, y_{i,t},\ s_i\, e_{i,t+1}\right)} > 1$.

[6]Different strategies have been used to address this kind of selection: twin studies (Behrman and Rosenzweig 2002), adoptees (Plug 2004; Jantti et al. 2006; Sacerdote 2007; Black et al. 2019; Fagereng, Mogstad, and Ronning), and policy changes affecting parents' outcomes exogenously (Black, Devereux, and Salvanes 2005). See Holmlund, Lindahl, and Plug (2011) and Black and Devereux (2011) for reviews.

C Identification

This appendix describes in more detail how our 13 moments identify the model's parameters and illustrates our identification strategy with simulations.

We identify the deep parameters of our model of human capital transmission with nepotism using the two Facts described in Section 3, Table 2. Specifically, we identify the intergenerational elasticity of human capital (β), the magnitude of nepotism (γ), the noise with which unobserved human capital is transformed into observed publications (σ_e and κ), and the shape of the human capital distribution (μ_h and σ_h) by minimizing the distance between 13 simulated and empirical moments in Table 2.[7] The 13 empirical moments used in the estimation can be grouped into two categories: First, as is standard in the literature, we consider correlations in observed outcomes across generations. Specifically, we consider the father-son correlation in publications conditional on both having at least one observed publication (intensive margin) and the proportion of families where father and son have zero publications (extensive margin). When observed, we also consider the grandfather-grandson correlation in the intensive margin. Second, we depart from the previous literature and consider ten moments describing the empirical distribution of publications for fathers and sons. These moments are the mean, the median, the 75th and 95th percentiles, and the proportion of zeros.

Before describing how the empirical moments used in the estimation identify the model's parameters, it is worth noting how γ, the magnitude of nepotism, depends on the other model's parameters. Specifically, γ is determined by parameters ν and τ, but also by the distribution of human capital among all potential scholars. In other words, $\tau - \nu$ alone does not characterize the magnitude of nepotism. For example, the same $\tau - \nu$ can reflect low levels of nepotism if the mean μ_h and the variance σ_h^2 of the stationary human capital distribution are high, and high levels of nepotism if μ_h and σ_h^2 are low. This is illustrated in Figure C.1, which shows the simulated distribution of human capital of sons of scholars under different model's parameters. All panels consider the same $\tau - \nu = 0.3$, but a different mean, μ_h, and variance, σ_h^2, for the human capital distribution. Specifically, the left panels consider a benchmarck scenario with $\mu_h = 1$ and $\sigma_h^2 = 1$. The top right panel considers a scenario with a larger mean ($\mu_h = 2$ and $\sigma_h^2 = 1$), and the bottom right panel a scenario with a larger variance ($\mu_h = 1$ and $\sigma_h^2 = 1.5$). Although ν is constant across panels, the share of nepotic sons varies considerably.

Next, we describe how our 13 empirical moments identify the model's parameters. Father-son correlations provide biased estimates of β due to measurement error, governed by σ_e and κ, and due to selection from nepotism, γ. We address both biases by comparing not only observed *outcomes* across generations, but also the corresponding *distributions*. These comparisons respond differently to measurement error and nepotism, and hence can be used to identify the model's parameters.

In terms of observed *outcomes*, an increase in measurement error reduces the extent to which father-son correlations reflect β. The reason is that measurement error alters these correlations but not the underlying human capital endowments. In contrast, an increase in nepotism alters the human

[7] The parameters μ_u and σ_u are pinned down from the stationarity conditions (6) and (7). We assume $\tau = 0$ without loss of generality and recover ν from Equation (9).

Figure C.1: The magnitude of nepotism (γ) and other model's parameters

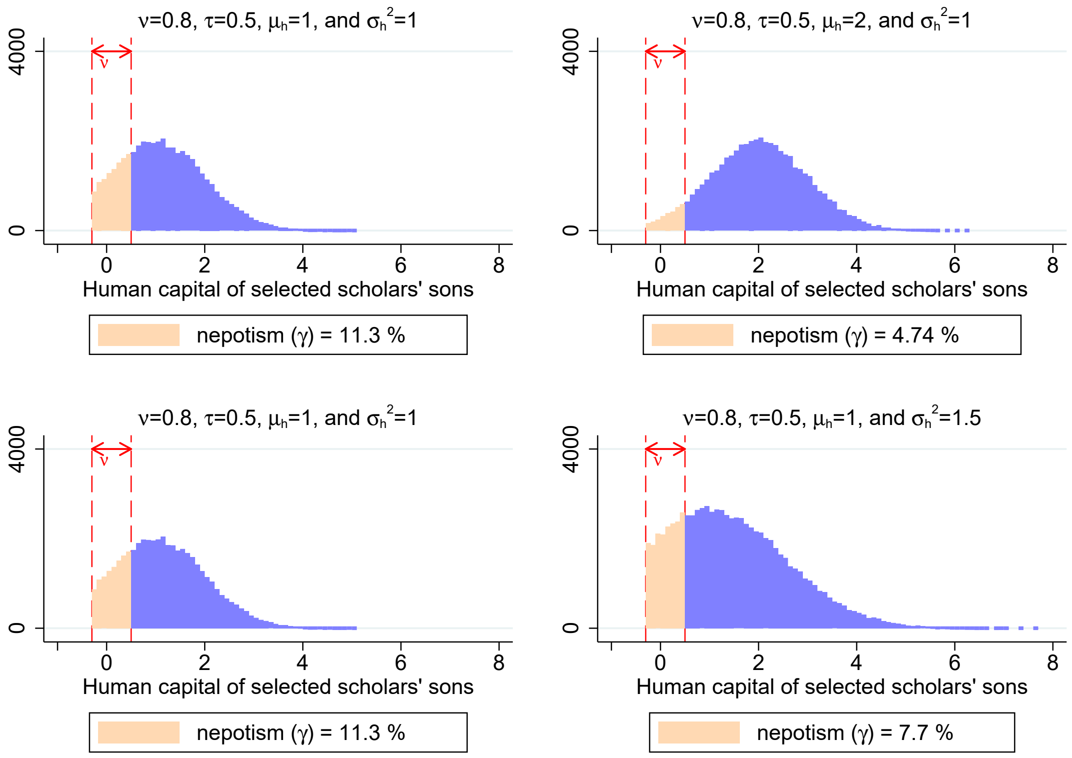

Notes: Based on 50,000 simulated families of potential scholars.

capital distributions for selected fathers and sons, and also the corresponding father-son correlations. Hence, these correlations may become more informative of β.

In terms of observed *distributions*, nepotism and measurement error also have different implications. Measurement error is not associated with differences in the distribution of the observed outcome y across generations. In contrast, nepotism lowers the selected sons' human capital relative to that of their fathers. This generates distributional differences across generations (beyond those generated by reversion to the mean), as suggested by Figure 5. Intuitively, the distributional differences generated by nepotism are stronger at the bottom of the distribution, i.e., closer to the selection thresholds. Our estimation strategy, hence, puts additional weight on the proportion of father's and sons with zero publications. In addition, the variance of the distributions—captured by the 75th and 95th percentiles—also helps to disentangle measurement error from nepotism: an increase in measurement error increases the variance of both distributions, while an increase in nepotism increases the variance of the sons' distribution relatively more. In theory, this allows to correct for measurement error without resorting to grandfather-grandson correlations. That said, in our empirical application measurement error is governed by two parameters, σ_e and κ. This additional moment, i.e. grandfather-grandson correlations, helps to identify σ_e and κ separately.[8]

[8] In other words, for datasets in which κ is not binding, the measurement error bias is governed by one parameter, σ_e. This can be identified with the variance of the observed outcome's distribution across generations, without resorting to

In sum, our identification strategy exploits the fact that an increase in the degree of nepotism (measurement error):

(i) generates (does not generate) father-son distributional differences;

(ii) increases (does not increase) the variance of sons' outcomes vs. their fathers';

(iii) increases (reduces) the information that father-son correlations convey about intergenerational human capital transmission.

Hence, by comparing both outcomes and distributions across generations, we can disentangle measurement error from selection and identify our model's parameters.

We illustrate our identification strategy with simulations. Figure C.2 shows the simulated distributions of the underlying (human capital) and the observed outcome (publications), father-son correlations in publications and the corresponding QQ plot. Column A presents a benchmark simulation for 10,000 potential scholars with $\beta = 0.6$, $\gamma = 13.5\%$, $\mu_e = 1$, $\pi = 0$, $\mu_h = 2$, $\sigma_h = 5$, $\sigma_e = 0.25$, $\tau = 0$, and $\nu = -1$. In Column B, we increase σ_e^2 to 3. That is, we generate measurement error by reducing the extent to which human capital translates into publications. The distribution of h is not altered with respect to the benchmark case, but that of y is: both fathers and sons present a larger mass of zero publications and a larger variance. Since y is similarly affected for fathers and sons, the QQ plot does not reflect distributional differences across generations. However, the increase in measurement error attenuates the father-son correlation in y, which drops from 0.45 to 0.27 with respect to the benchmark case.

Column C increases nepotism with respect to the benchmark case by setting $\gamma = 40\%$ (or, alternatively, by setting $\nu = -2.5$ with the remaining model parameters being constant). In contrast to the previous exercise, this affects the distribution of both h and y, as sons with low levels of human capital now can become a scholar.[9] This generates distributional differences in observed publications between fathers and sons, reflected in the QQ plot. Most evidently, the mass of sons with zero publications and the variance of sons' publications is now larger than their fathers'. Since nepotism alters both the human capital's and the observed outcome's distribution, father-son correlations become more informative of β than in the benchmark case: the correlation increases from 0.45 to 0.47.

In sum, measurement error and nepotism have different implications for father-son correlations, distributional differences (especially, at the bottom of the distribution), and the relative variances of observed outcomes.

grandfather-grandson correlations.

[9]The father's h distribution is also affected, albeit to a lesser degree. The reason is that marginal fathers, i.e., fathers with an h just above the threshold τ, are now more likely to be in the set of selected families. Before, these fathers were mostly excluded, as their sons were likely to have low realizations of h, falling below the (nepotic) threshold to become a scholar. Similarly, this may decrease the variance of fathers' publications.

Figure C.2: Identification example based on model simulations

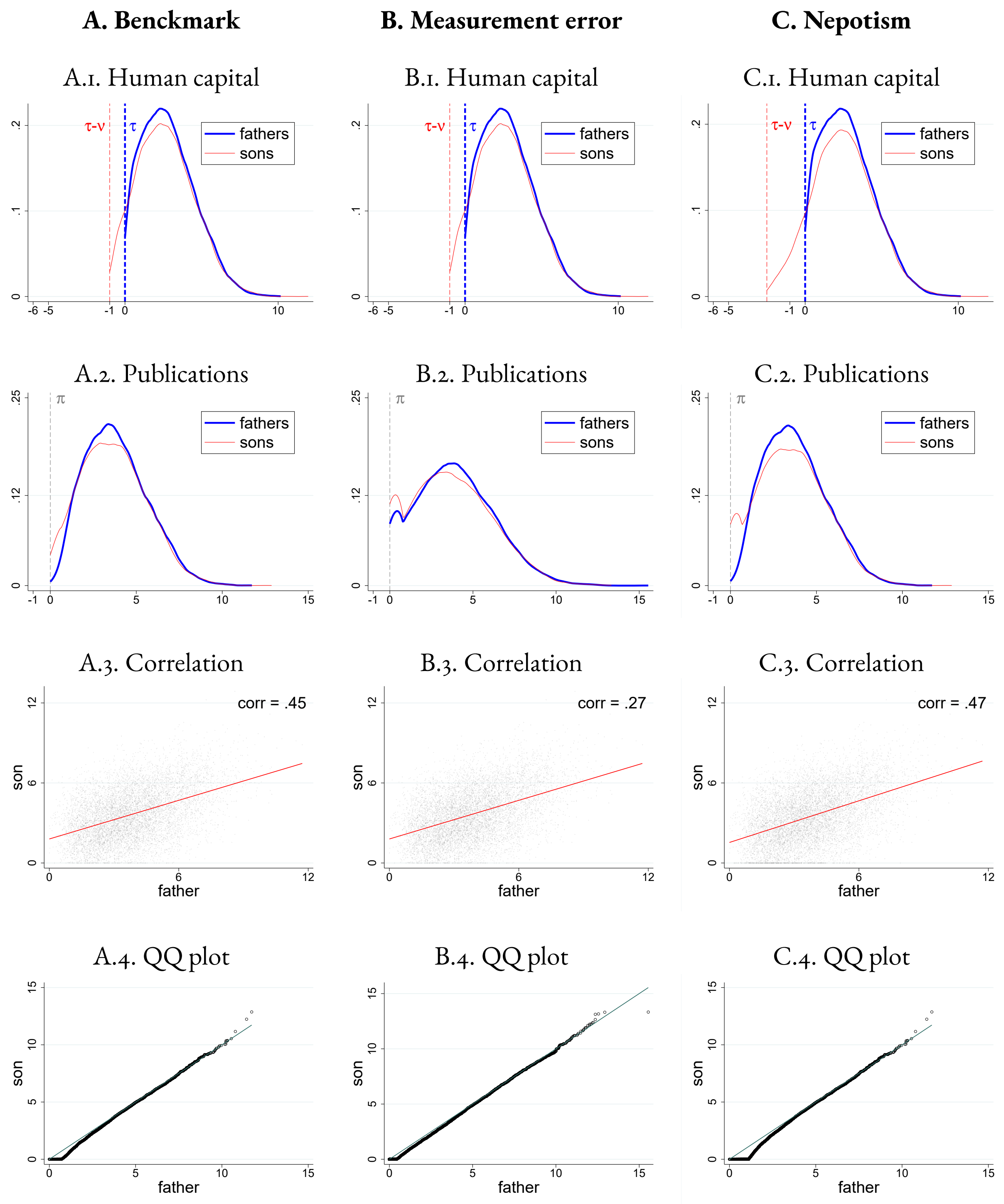

Notes: The benchmark simulation is for 10,000 potential scholars with $\beta = 0.6$, $\gamma = 13.5\%$, $\mu_e = 1$, $\pi = 0$, $\mu_h = 2$, $\sigma_h = 5$, $\sigma_e = 0.25$, $\tau = 0$, and $\nu = -1$. Column B increases σ_e to 3, Column C increases nepotism by setting $\gamma = 40.2\%$.

D Model fit

This appendix provides additional descriptive statistics and a detailed discussion on model fit, which is summarized in the main text (see Section 5.2).

Table D.1 shows the 6 parameter estimates and the 13 empirical and simulated moments for our baseline model as well as for the alternative model without nepotism discussed in Section 5.2. Panel A presents parameter estimates, Panel B empirical and simulated moments. Specifically, the top rows present the simulated and empirical moments regarding correlations across generations, and serve to evaluate Fact 1; the bottom rows evaluate the fit for the fathers' and sons' marginal distribution of publications, and serve to evaluate Fact 2.

Our baseline estimates reproduce Fact 1. Our model with nepotism matches the father-son correlation on the intensive margin of publications – that is, conditional on both father and son having at least one observed publication. This is the correlation to which our objective function attaches additional weight. Interestingly, this correlation is below the estimate of β. This implies that father-son correlations in outcomes under-predicts the extent to which children inherit human capital endowments from their parents. Our model with nepotism matches the proportion of families where father and son have zero publications (extensive margin) and the correlation between grandfathers and grandsons in the intensive margin. That said, we slightly under-predict these moments compared to the model without nepotism. Importantly, our baseline model matches the empirical fact that the grandfather-grandson correlation is larger than predicted by iterating the two-generation correlation. Specifically, our simulated grandfather-grandson correlation is 0.189. In contrast, iterating the simulated two-generation correlation yields $0.375^2 = 0.1406$.

Our estimates also reproduce Fact 2, that is, that the publications' distribution of fathers first-order stochastically dominates that of sons. Table D.1 shows that we fit both distributions: we perfectly match the proportion of fathers and sons with zero publications—the two moments to which our objective function attaches additional weight. We also match the sons' mean and median. For fathers, we underestimate the number of publications, especially in the 75th percentile. That said, we reproduce the father-son distributional differences described in Fact 2. We match the fact that fewer fathers have zero publications, that fathers on average published more than sons, and that the median father, the father on the 75th, and on the 95th percentile published more than the corresponding sons. We also reproduce the empirical observation that the gap between fathers' and sons' publications is more prominent at the bottom of the distribution: our simulated moments reflect larger father-son gaps in the mean and the median than in the 75th and 95th percentile.

As explained in the main text, nepotism is crucial for reproducing the father-son distributional differences. Specifically, we estimate an alternative model without nepotism, $\gamma = 0$. This model, where distributional differences can only be generated by mean reversion, fails to match Fact 2, and only generates very small distributional differences above the median.

Table D.1: Simulated and empirical moments for different models

	Model w/o nepotism	Baseline model	Data
A. Parameters:			
β	0.723	0.634	.
γ	0 *(imposed)*	18.74	.
μ_h	4.853	1.865	.
σ_h	2.204	4.219	.
σ_e	1.119	0.393	.
κ	3.989	2.121	.
B. Moments:			
Father-son correlation†	0.375	0.375	0.375
Father-son with zero publications	0.205	0.170	0.211
Grandfather-grandson correlation†	0.245	0.189	0.234
Fathers with zero publications	0.350	0.289	0.288
Sons with zero publications	0.350	0.383	0.384
Median, fathers	4.921	3.678	5.075
Median, sons	4.918	3.231	3.402
75th percentile, fathers	6.584	5.963	7.370
75th percentile, sons	6.574	5.832	6.413
95th percentile, fathers	8.979	9.612	9.425
95th percentile, sons	8.888	9.539	8.537
Mean, fathers	4.110	3.844	4.456
Mean, sons	4.105	3.463	3.477

Notes: †correlation on the intensive margin.

E Additional figures and tables

E.1 QQ plots

FIGURE E.1: Quantile-quantile plot by historical period

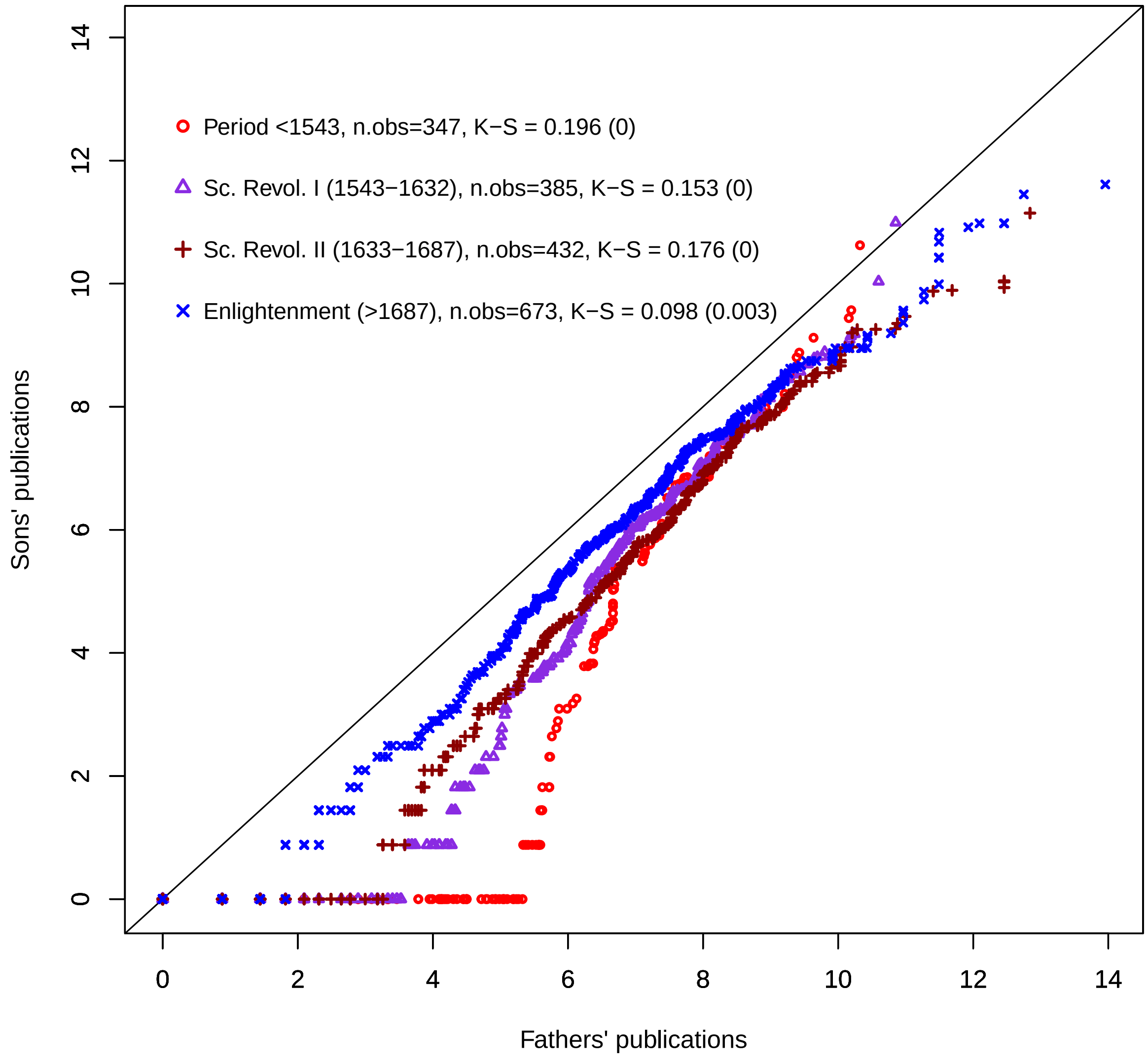

Figure E.2: Quantile-quantile plot by age of institution

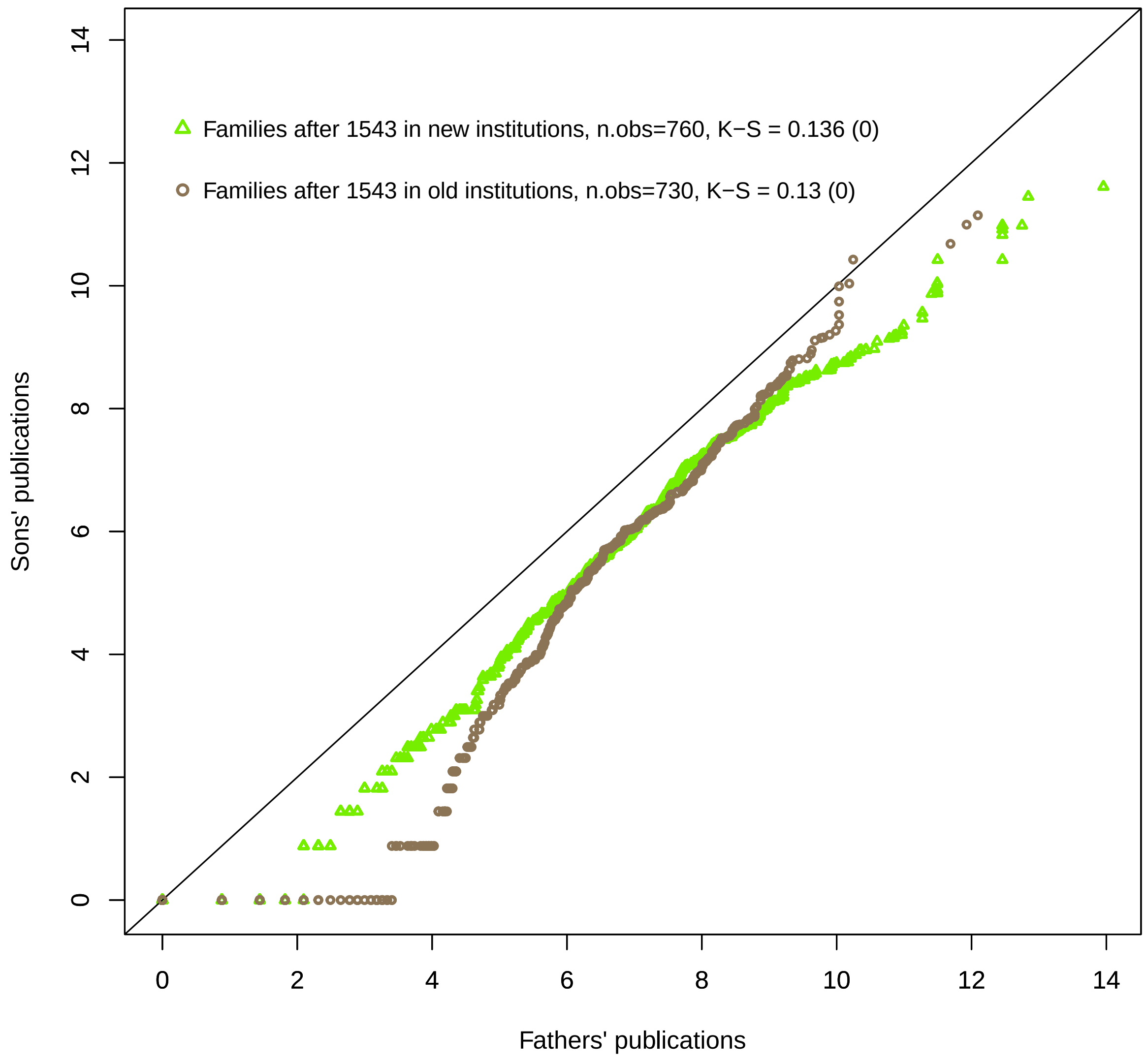

Figure E.3: Quantile-quantile plot by religious affiliation

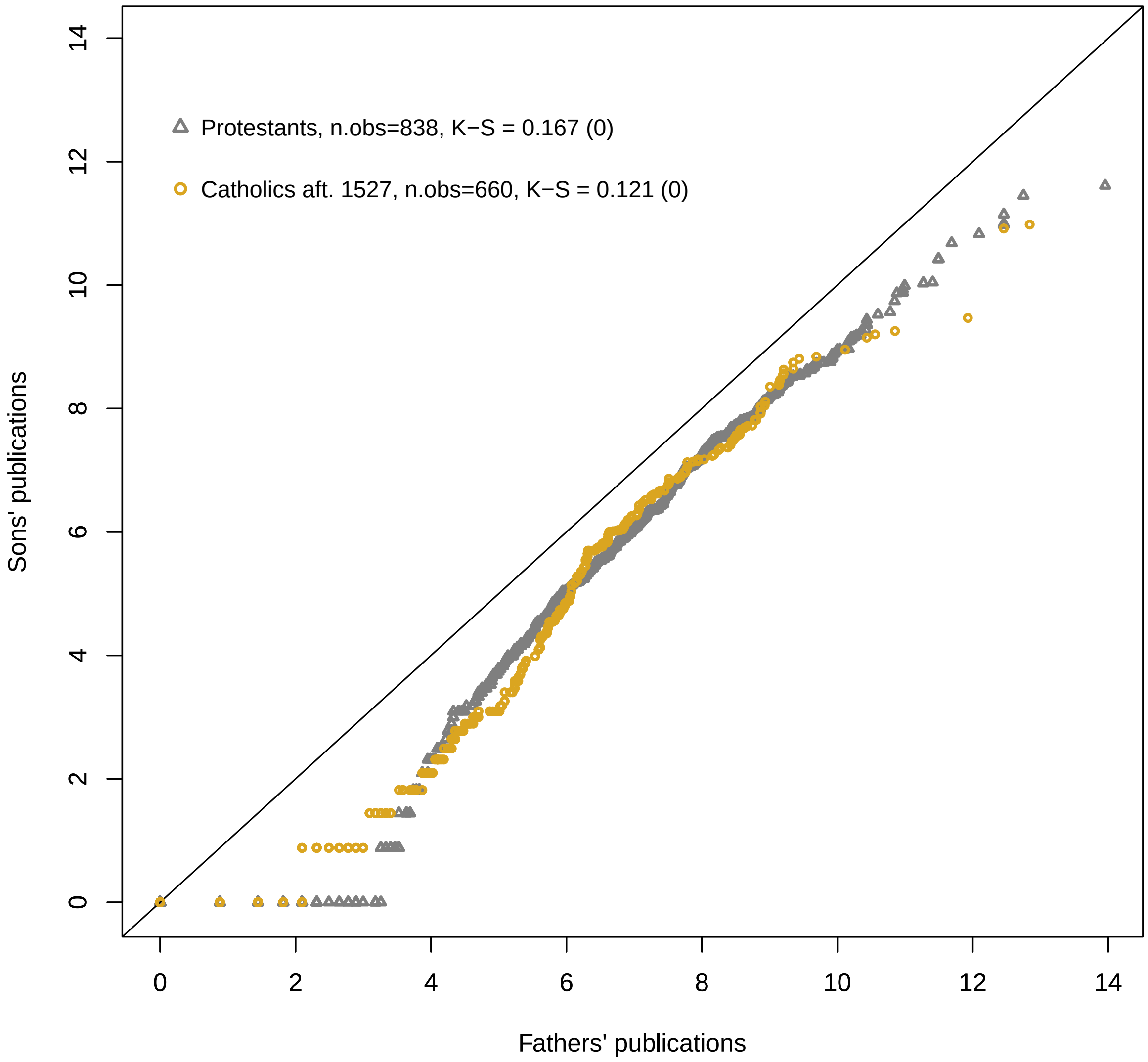

FIGURE E.4: Quantile-quantile plot by field of study

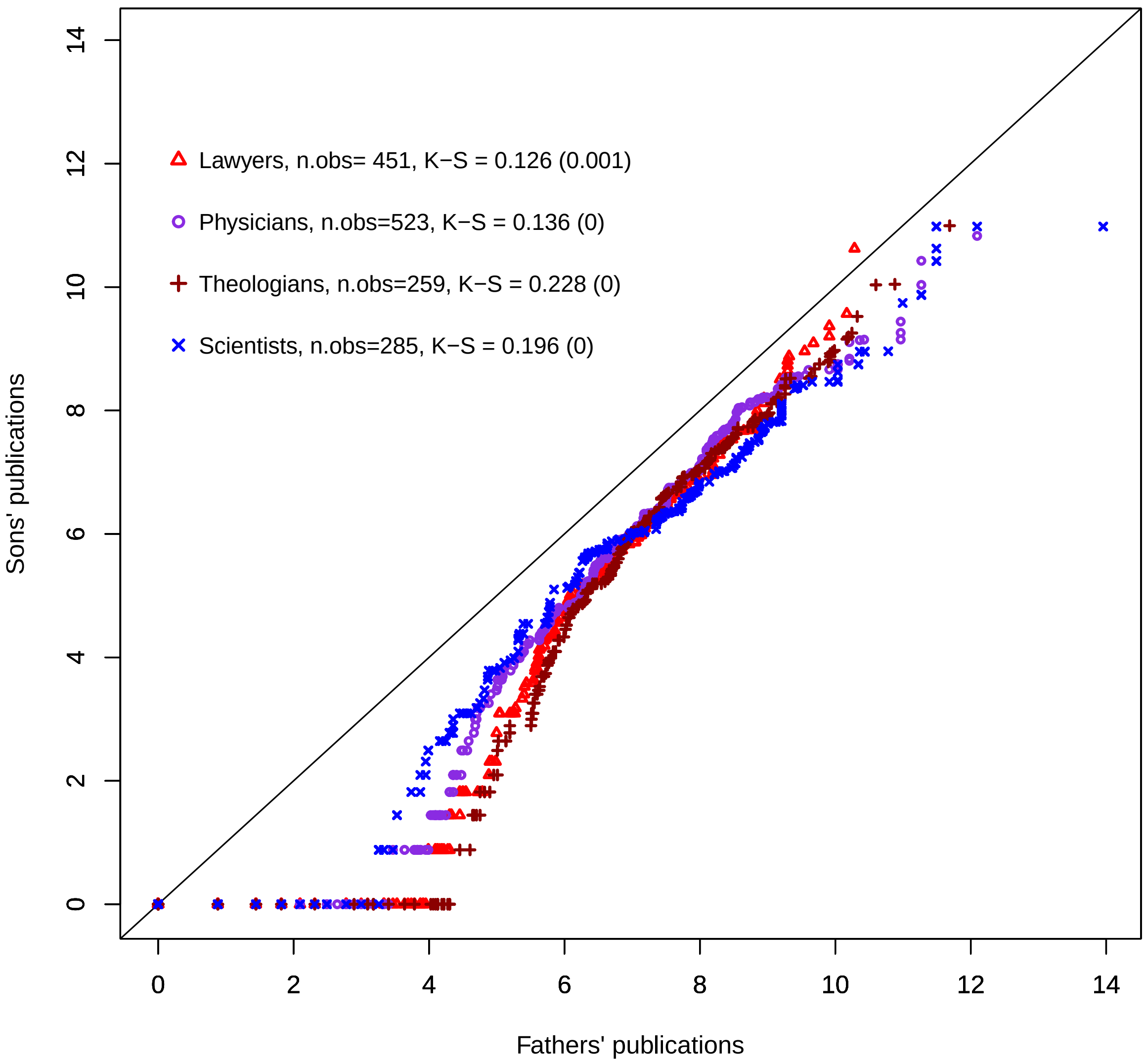

Figure E.5: Quantile-quantile plot by fathers and sons in same vs different fields

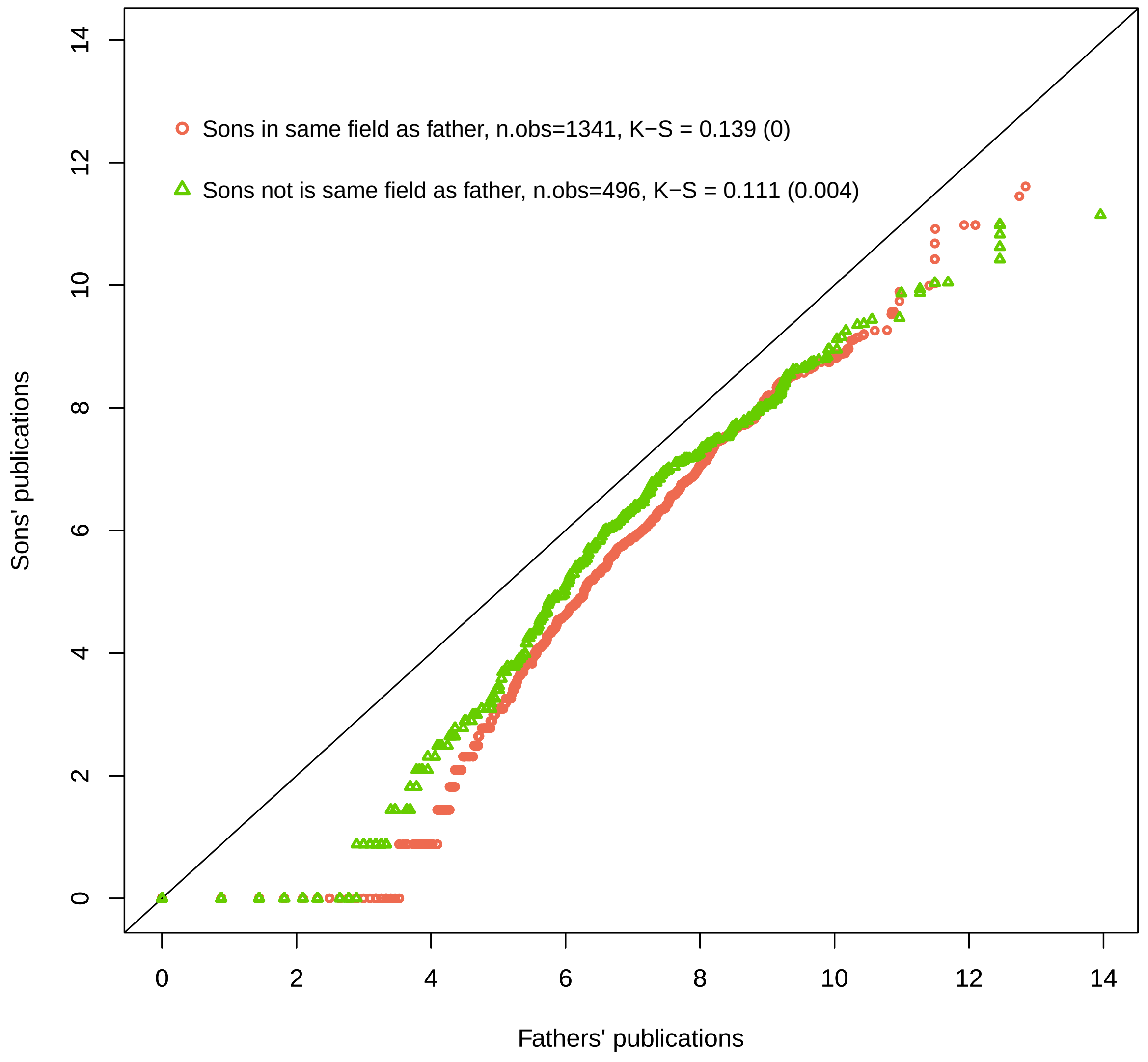

Figure E.6: Quantile-quantile plot by nomination before/after father's death

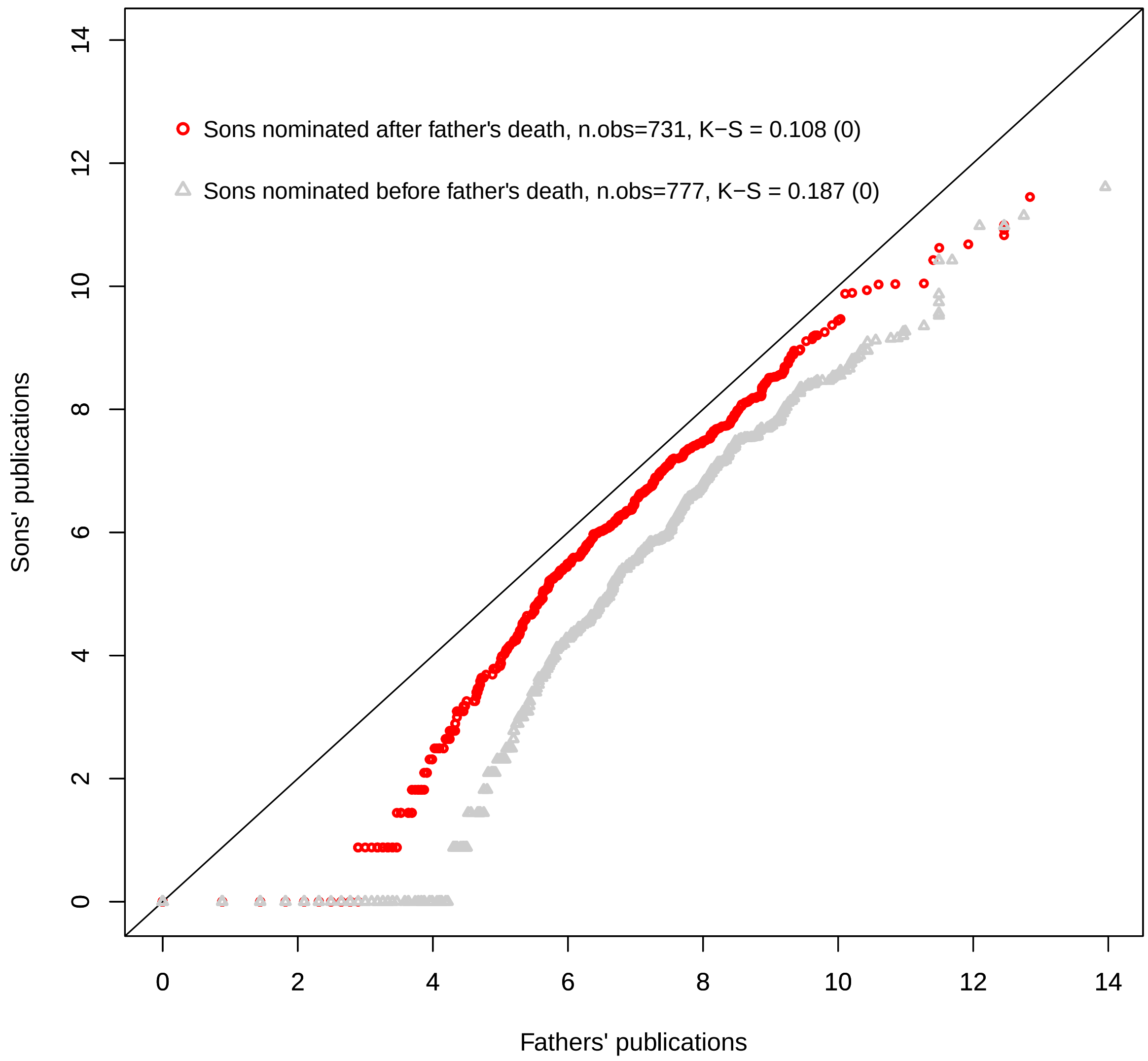

FIGURE E.7: Quantile-quantile plot by type of institutions

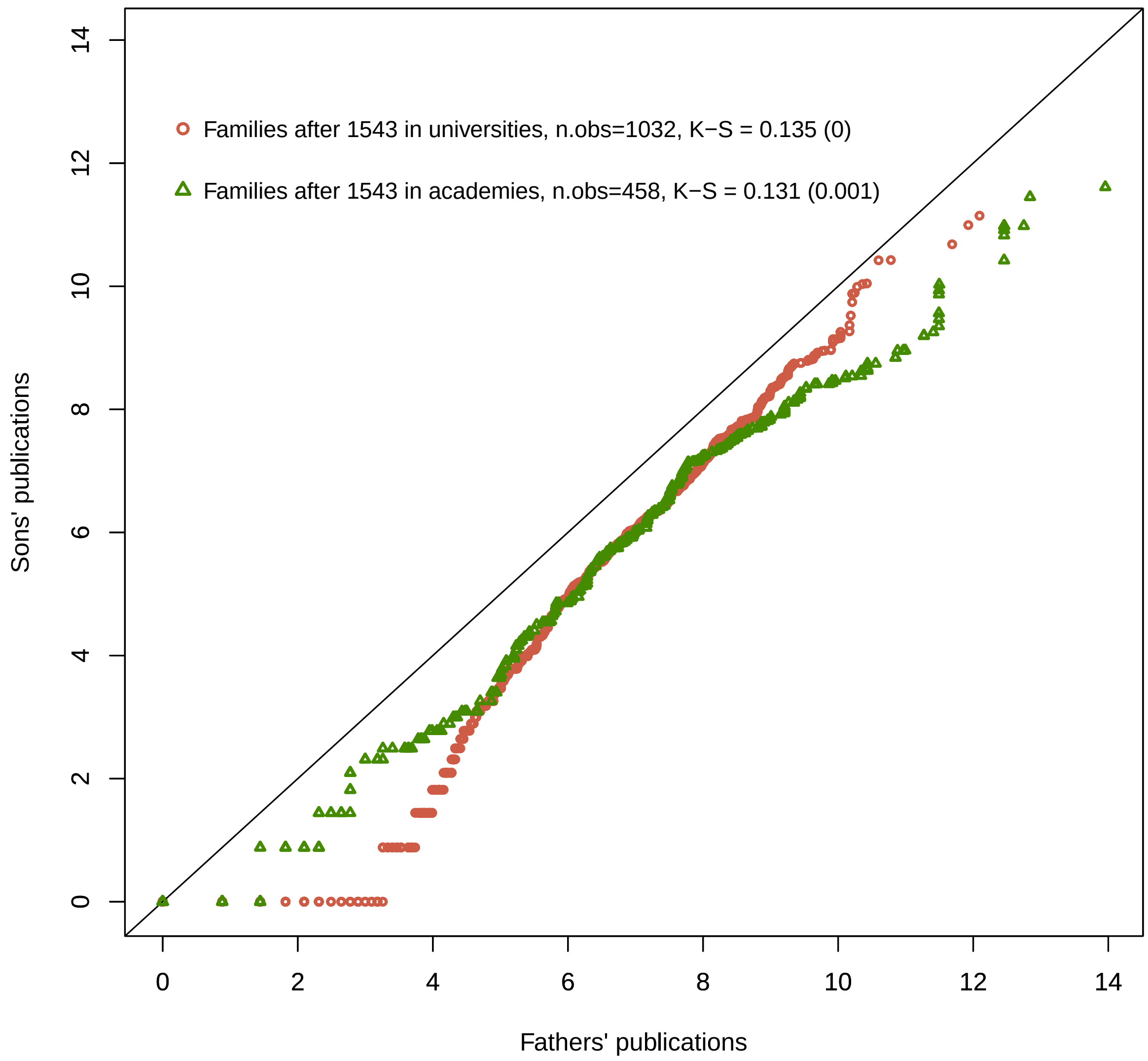

E.2 Additional figures and tables for heterogeneity analysis

Figure E.8: Field-institution growth rates in publications over time

A. Catholic institutions

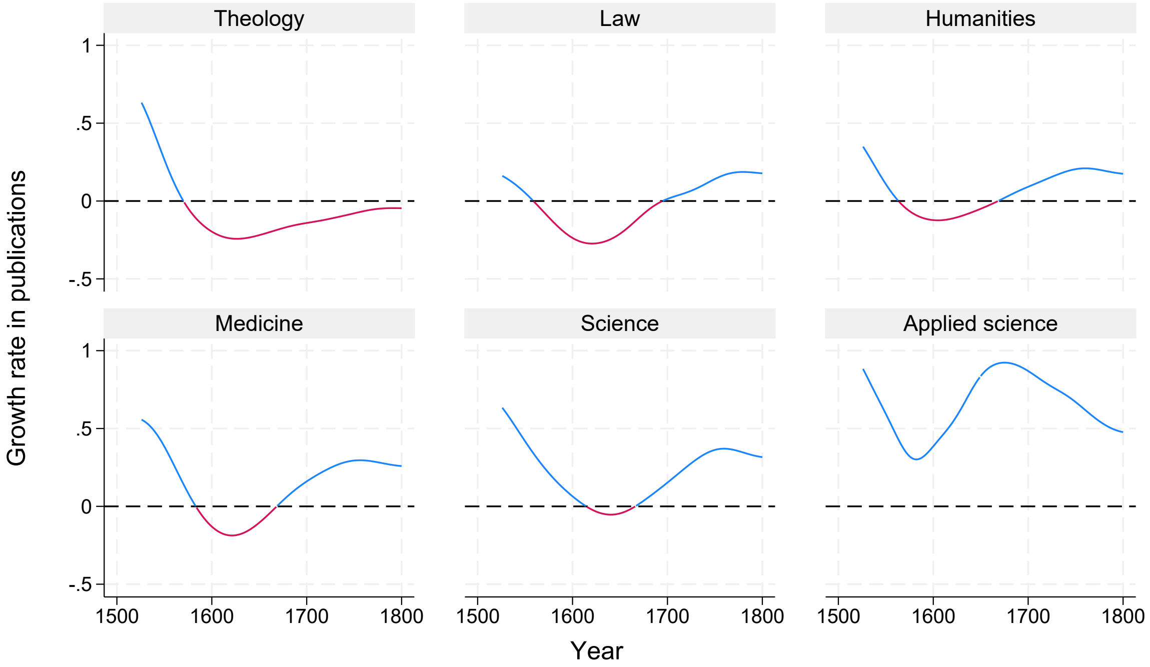

B. Protestant institutions

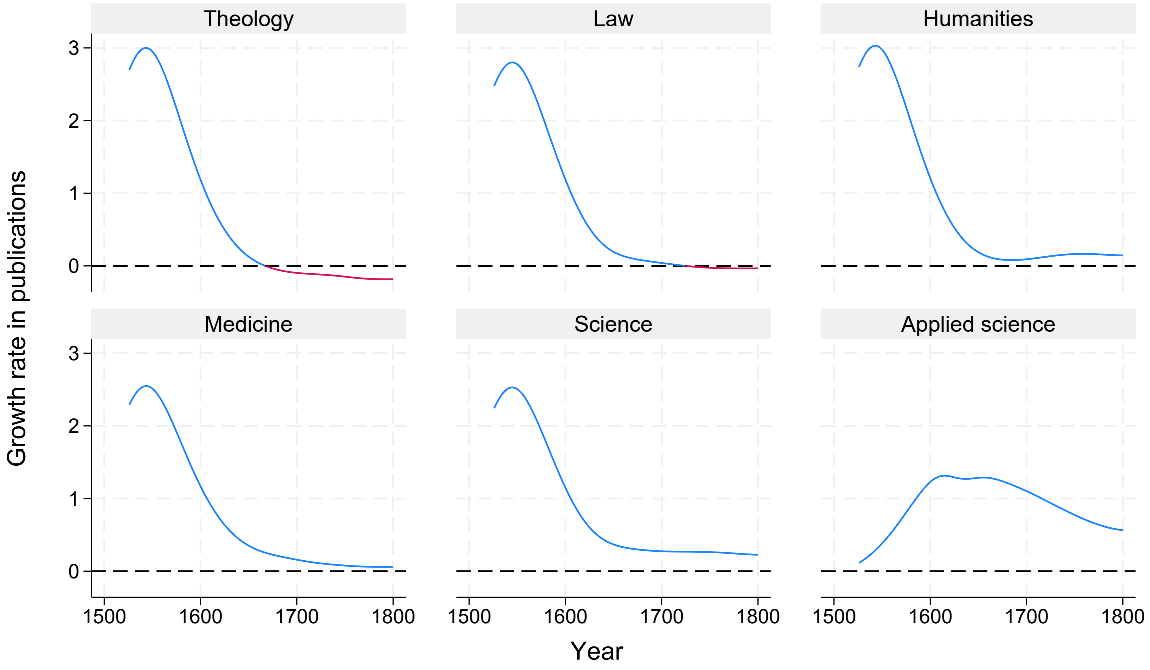

Notes: This figure uses data from De la Croix (2021b) on 40,800,000 publications of all known scholars active between 1500–1800. It displays, for each year, the growth rate in publications over the previous 25 years by field of study and type of institution (catholic vs. protestant). Blue is for eras of rapidly changing knowledge frontier (growth rate > 0); red is for eras of stagnation (growth rate ≤ 0).

Table E.1: Heterogeneity by rapid vs. stagnant eras, under different thresholds for growth rate of publications (g)

		[1]	[2]	[3]	[4]
		Rapidly growing knowledge frontier $g > 0$	Stagnant knowledge frontier $g \leq 0$	Rapidly growing knowledge frontier $g > median$	Stagnant knowledge frontier $g \leq median$
IGE human capital	β	0.64	0.78	0.65	0.67
		(0.04)	(0.06)	(0.06)	(0.05)
Nepotism, %	γ	9.2	25.3	7.3	18.21
		(2.3)	(4.1)	(3.36)	(2.32)
Mean human capital	μ_h	3.7	-1.1	3.98	1.79
		(0.5)	(1.3)	(0.67)	(0.68)
SD human capital	σ_h	3.5	5.0	3.33	4.30
		(0.3)	(0.5)	(0.50)	(0.28)
SD publications' shock	σ_e	0.3	0.7	0.25	0.38
		(0.1)	(0.2)	(0.22)	(0.16)
Threshold publications	κ	2.1	1.8	2.24	1.89
		(0.3)	(0.3)	(0.54)	(0.18)
N		1,048	290	670	668

Notes: SE in parenthesis from 200 bootstrapped samples with replacement; degrees of overidentification: 6

E.3 Additional figures and tables for validation exercises

TABLE E.2: Dep. variable = 1 if scholar has Wikipedia page.

	[1]	[2]	[3]	[4]	[5]
Scholar's son (0/1)	0.074***	0.081***	0.075***	0.059***	0.062***
	(0.014)	(0.014)	(0.013)	(0.013)	(0.015)
Publications (arcsinh)	0.106***	0.106***	0.097***	0.094***	
	(0.001)	(0.001)	(0.001)	(0.001)	
Cohort FE	.	Y	Y	Y	Y
Institution FE	.	.	Y	Y	Y
Field FE	.	.	.	Y	Y
Number of library holdings FE	.	.	.	.	Y
Observations	20,500	20,394	20,354	20,354	18,616
R-squared	0.262	0.292	0.384	0.397	0.425

Notes: The sample is 20,500 scholars from institutions with complete and broad coverage who are listed in Worldcat. Cohort fixed effects are based on a scholar's earliest activity dat, and institution fixed effects on a scholar's first appointment. For scholars working in multiple fields, we consider the main field. Standard errors clustered by cohort in parenthesis; *p<.05; **p<.01; ***p<.001.

Table E.3: Eighteen moments targeted in extended estimation using outsiders

		value	s.e.	N
A. Intergenerational correlations				
1. Father-son, intensive margin	$\rho(y_t, y^s_{t+1} \mid_{y_t, y_{t+1}>0})$	0.375	0.032	982
2. Father-son with zero publications	$\Pr(y_t=0 \wedge y^s_{t+1}=0)$	0.211	0.004	1,482
3. Grandfather-grandson, intensive margin	$\rho(y_t, y^g_{t+2} \mid_{y_t, y^g_{t+2}>0})$	0.234	0.172	87
B. Distributional moments				
4. Fathers with zero publications	$\Pr(y_t=0)$	0.131	0.009	1,328
5. Outsiders with zero publications	$\Pr(y^o_{t+1}=0)$	0.030	0.002	9,118
6. Sons with zero publications	$\Pr(y^s_{t+1}=0)$	0.236	0.011	1,482
7. Fathers median	$Q50(y_t)$	6.050	0.117	1,328
8. Outsiders median	$Q50(y^o_{t+1})$	6.085	0.031	9,118
9. Sons median	$Q50(y^s_{t+1})$	4.927	0.140	1,482
10. Fathers 75th percentile	$Q75(y_t)$	7.714	0.082	1,328
11. Outsiders 75th percentile	$Q75(y^o_{t+1})$	7.616	0.031	9,118
12. Sons 75th percentile	$Q75(y^s_{t+1})$	6.893	0.092	1,482
13. Fathers 95th percentile	$Q95(y_t)$	9.656	0.163	1,328
14. Outsiders 95th percentile	$Q95(y^o_{t+1})$	9.616	0.044	9,118
15. Sons 95th percentile	$Q95(y^s_{t+1})$	8.689	0.081	1,482
16. Fathers mean	$E(y_t)$	5.439	0.083	1,328
17. Outsiders mean	$E(y^o_{t+1})$	5.738	0.027	9,118
18. Sons mean	$E(y^s_{t+1})$	4.310	0.080	1,482

Notes: The sample comprises (a) families of scholars and (b) outsiders in the same cohorts and institutions as at least one scholar's son. Note that this sample is different from that in Table 2 because here we take a conservative approach and restrict the sample to outsiders and families of scholars who are listed in Worldcat or Wikipedia; y: publications (inverse hyperbolic of library holdings by or about each scholar).

Table E.4: Fathers and sons at different universities

			Baseline sample		Different universities	
Parameters:	Interg. elasticity human capital	β	0.63	(0.04)	0.81	(0.15)
	Nepotism magnitude, %	γ	18.7	(1.74)	0.04	(0.07)
	Mean human capital distribution	μ_h	1.87	(0.47)	5.79	(0.31)
	S.D. human capital distribution	σ_h	4.22	(0.20)	2.13	(0.20)
	S.D. shock to publications	σ_e	0.39	(0.15)	2.26	(0.47)
	Threshold observable publications	κ	2.12	(0.14)	2.25	(0.58)
Data moments:	Fathers with zero publications		0.29		0.16	
	Sons with zero publications		0.38		0.10	
	Median, fathers		5.08		6.12	
	Median, sons		3.40		6.92	
	75th percentile, fathers		7.37		7.63	
	75th percentile, sons		6.41		8.10	
	95th percentile, fathers		9.43		9.47	
	95th percentile, sons		8.54		9.55	
	Mean, fathers		4.46		5.37	
	Mean, sons		3.48		6.13	
	Father-son correlation†		0.38		0.29	
	Father-son with zero publications		0.21		0.05	
	Grandfather-grandson correlation†		0.23		-0.10	
	N		1,837		507	

Notes: †on the intensive margin. SE from 200 bootstrapped samples with replacement.

F Moments used in estimation with complete and complete & broad coverage

In the main text, we examine the sensitivity of our analysis to sampling bias. That is, to the possibility that the secondary sources used to construct our dataset selectively report father-son links when fathers are famous. In short, we show that this scenario is unlikely for four reasons: First, almost two thirds of our father-son pairs are based on sources with complete coverage where we can rule out sampling bias, and the remaining third comes mostly from sources whit broad coverage where sampling bias is unlikely. Second, the coverage of the data (complete, broad, or partial) does not vary substantially over the historical periods under analysis, over centuries, across countries, across fields of study, and by the religion of the university (protestant vs. catholic). Third, it is not obvious why secondary sources would selectively record famous fathers of underachieving son more often than underachieving fathers of famous sons. Fourth, we present separate estimates restricting the data to sources with complete coverage and to sources with complete and broad coverage, and show that our results are robust. In this appendix, we provide the detailed summary statistics of the moments used in the estimations with complete coverage and with complete and broad coverage. We show that the two Facts used in our estimation strategy are robust to the accuracy of our sources, and hence, are also not a by-product of sampling bias.

Specifically, Table F.1 presents the 13 moments used for our baseline estimation (column 1), for our estimation restricted to sources with complete coverage (column 2), and for our estimation restricted to sources with complete and broad coverage (column 3). Panel A shows the moments capturing intergenerational correlations. If the historical sources used are subject to sampling bias, we would expect our baseline intergenerational correlations to be downward biased relative to those calculated using sources where we can rule out sampling bias (Solon 1989). Instead, we find that the father-son elasticity of publications is 0.375 for all families, 0.36 for families with complete coverage only, and 0.38 for families with complete and broad coverage. Similarly, the grandfather-grandson elasticity of publications is, respectively, 0.23, 0.18, and 0.23. On the extensive margin, the proportion of fathers *and* sons with zero publications is around 0.20 for all families, families with complete coverage, and families with complete and broad coverage. This suggests that the relatively high elasticity of publications across generations (Fact 1) is not a by-product of sampling bias in our sources, as it is observed also in the subsample restricted to complete coverage where we observe the universe of father-son pairs.

Panel B of Table F.1 shows the moments capturing father-son distributional differences. If fathers who were scholars of no great account are more likely to fall by the wayside than an underachieving son of a famous scholar, we would expect this sampling bias to drive the wedge between the fathers' and sons' publication distribution. In other words, we would expect our baseline distributional differences to be substantially larger than those calculated using sources where we can rule out sampling bias. Instead, we find that the distributional moments are very similar for all families, for families with complete coverage only, and for families with complete and broad coverage. For example, the

Table F.1: Moments, by coverage of data sources

		All [1]	Complete [2]	Complete and Broad [3]
A. Intergenerational correlations				
Father-son, intensive margin	$\rho(y_t, y_{t+1} \|_{y>0})$	0.375	0.36	0.38
Father-son with zero publications	$\Pr(y_t=y_{t+1}=0)$	0.21	0.18	0.21
Grandfather-grandson, intensive margin	$\rho(y_t, y_{t+2} \|_{y>0})$	0.23	0.18	0.23
B. Father-son distributional differences				
Fathers with zero publications	$\Pr(y_t=0)$	0.29	0.26	0.29
Sons with zero publications	$\Pr(y_{t+1}=0)$	0.38	0.33	0.38
Fathers median	$Q50(y_t)$	5.08	5.43	5.11
Sons median	$Q50(y_{t+1})$	3.40	4.34	3.58
Fathers 75th percentile	$Q75(y_t)$	7.37	7.51	7.37
Sons 75th percentile	$Q75(y_{t+1})$	6.41	6.74	6.44
Fathers 95th percentile	$Q95(y_t)$	9.43	9.68	9.44
Sons 95th percentile	$Q95(y_{t+1})$	8.54	8.63	8.55
Fathers mean	$E(y_t)$	4.46	4.73	4.49
Sons mean	$E(y_{t+1})$	3.48	3.86	3.53
Father-son pairs	N	1,837	1,178	1,763

proportion of sons with zero publications is 9, 7, and 9 percentage points larger than the proportion of fathers with zero publications in, respectably, our baseline sample with all observations, observations with complete coverage, and observations with complete and broad coverage. The median, 75th and 95th percentile, and mean are also larger for fathers than for sons across these three groups.

To illustrate the similarity of the father-son distributional differences, Figure F.1 presents QQ plots for each group. The fathers' distribution of publications first order stochastically dominates that of sons independently of the coverage of the sources. Altogether, this shows that the distributional differences between fathers and sons (Fact 2) holds when we restrict our data to complete sources where we can rule out sampling bias. In other words, it is highly unlikely that the data is selected on father's publications and that this drives the observed wedge between the publications of fathers vs. sons.

Figure F.1: Quantile-quantile, by data coverage

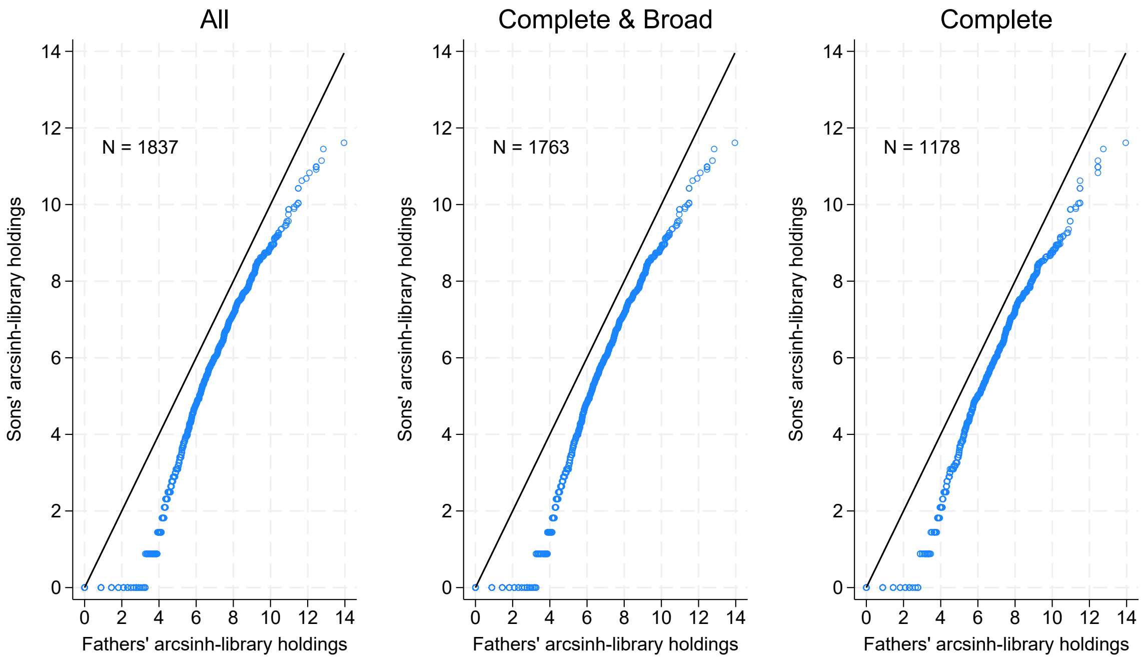

G Stationarity and time trends in publications

To estimate nepotism and the intergenerational human capital elasticity, we assume that the human capital distribution is stationary among *potential scholars*. That is, among individuals with high human capital endowments who could potentially become scholars—whether they are in our dataset or not. This assumption is standard in the literature estimating intergenerational elasticities, but its importance is rarely discussed (Nybom and Stuhler 2019). In this appendix, we first discuss the use of the stationarity assumption in the literature and the sensitivity of our β-estimates to it. Next, we show that, under stationarity, our (already large) nepotism estimates are a lower-bound to the true level of nepotism. Finally, we use a dataset on all pre-modern scholars (not only fathers and sons) collected by De la Croix (2021b) to examine time trends in observed outcomes. These trends support the stationarity assumptions for both our nepotism and β-estimates. In addition, Section 5.3 of the main text relaxes the stationarity assumption. Specifically, we assume that the human capital of a father and a son who were active in a given time period is drawn from the same distribution, but we allow the human capital distribution to change across periods. This allows publications to exhibit time trends on both the extensive or intensive margin.

G.1 Stationarity in the intergenerational literature

Theory. Steady-state assumptions play a critical role for intergenerational elasticities, especially when the endowments that parents transmit to children are unobserved.[10] To see this, consider the following first-order Markov process:

$$\begin{aligned} h_{i,t+1} &= \beta h_{i,t} + u_{i,t+1}\,, \\ y_{i,t+1} &= h_{i,t+1} + \varepsilon_{i,t+1}\,, \end{aligned}$$

where $h_{i,t} \sim N(\mu_{h,t}, \sigma^2_{h,t})$ is an unobserved endowment (human capital) that parents t transmit to children $t+1$ at rate β; y is an observed outcome (publications) noisily related to h; and $u_{i,t+1}$ and $\varepsilon_{i,t+1}$ are noise terms with standard deviation σ_u and σ_e. Here $\mu_{h,t}$ and $\sigma_{h,t}$ are time dependant. In other words, we do not impose stationarity over the human capital distribution.

We can estimate β using correlations in y across multiple generations.[11] The OLS elasticity of y between parents and children (b_1) and the corresponding elasticity between grandparents and grandchildren (b_2) are:

$$\begin{aligned} b_1 &= \beta \left[\sigma^2_{h,t+1} \,/\, (\sigma^2_{h,t+1} + \sigma^2_{\varepsilon})\right]\,, \\ b_2 &= \beta^2 \left[\sigma^2_{h,t+2} \,/\, (\sigma^2_{h,t+2} + \sigma^2_{\varepsilon})\right]\,, \end{aligned}$$

Hence, the ratio b_2/b_1 identifies β under the assumption that $\sigma_{h,t+1} = \sigma_{h,t+2}$. That is, when the signal-to-noise ratio is constant across three generations. This condition is satisfied when the human capital

[10] See, e.g., Clark and Cummins (2015), Adermon, Lindahl, and Waldenström (2018).
[11] Lindahl et al. (2015), Braun and Stuhler (2018), Colagrossi, d'Hombres, and Schnepf (2019).

distribution is stationary. However, this stationarity assumption is often implicit, and its importance in estimating β is rarely acknowledged in the literature (Nybom and Stuhler 2019).

Evidence. Next, we present evidence supporting the stationarity assumption $\sigma_{h,t+1}=\sigma_{h,t+2}$. Ideally, we would show that the standard deviation of human capital h is constant over time for the universe of *potential scholars*. Since, by construction, we do not observe h, we will show trends in the standard error of the mean for our observed human-capital proxy: publications. To evaluate a universe resembling all *potential scholars*, we use the De la Croix (2021b) data on 58,251 pre-modern scholars (not only fathers and sons) with a reference date in 1088–1800.[12]

Figure G.1 presents these trends, calculated over 25-year intervals. After 1350, the standard error of the mean of log-publications is extremely stable. This supports the assumption of a stable variance in the human capital distribution over time, that is, that $\sigma_{h,t+1} = \sigma_{h,t+2}$ is satisfied. Admittedly, the standard error is much larger before 1350. That said, in our dataset we have 36 families where both father and son's reference date is before 1350. Hence, it is unlikely that the large changes in standard error before 1350 are driving our aggregate β-estimates.

Figure G.1: Trend in standard error of the mean, arcsinh-library holdings

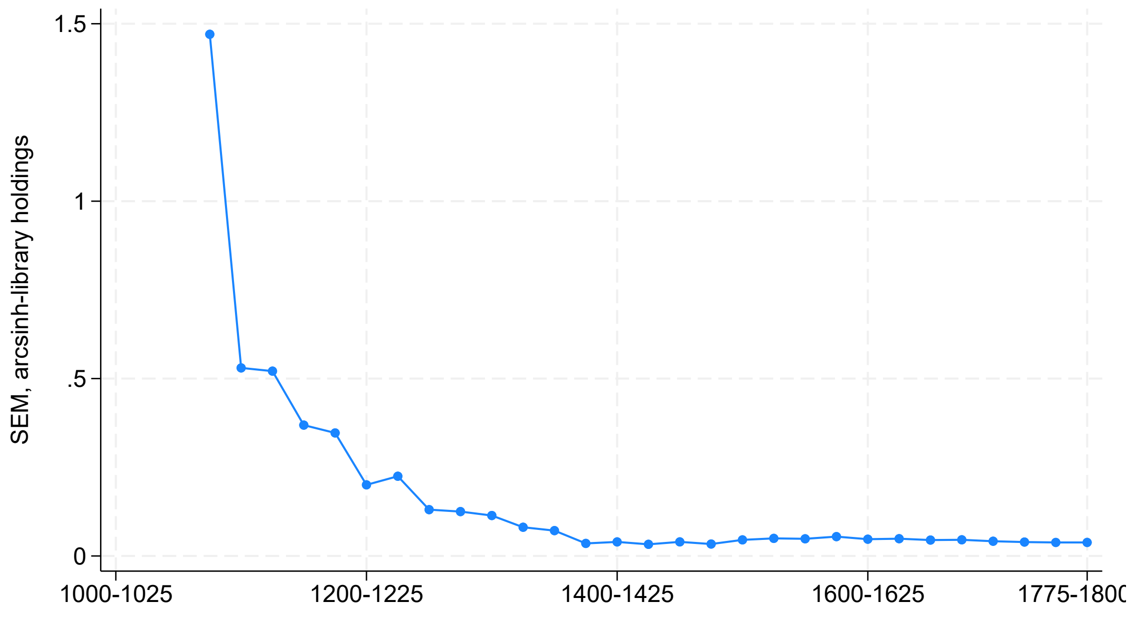

Notes: The sample is all scholars in De la Croix (2021b) with a reference date between 1088 and 1800 ($N = 58,251$). Standard error of the mean in arcsinh-library holdings calculated over 25-year periods.

[12] Note that here we do not restrict the sample to institutions with a certain data coverage or to individuals listed in WorldCat. Hence, this sample is larger than the one used in Figure 1. Reference dates are based on birth year, nomination year, or approximate activity year.

G.2 Stationarity and nepotism

Theory. Our estimates for nepotism are also sensitive to the stationarity assumption. Here we argue that, under stationarity, our nepotism estimates are lower-bound estimates. Note that we identify nepotism using two sets of moments: The first are correlations in observed outcomes across multiple generations. These allow us to uncover the true intergenerational human capital elasticity, which will be important to estimate nepotism. The second are distributional differences in observed outcomes between fathers and sons. We argue that the observed distributional differences may be the result of two forces: on the one hand, nepotism lowers the selected sons' human capital relative to that of the selected fathers, generating distributional differences in publications. That said, not all the distributional differences are directly attributed to nepotism. The second force at place is mean-reversion. If human capital strongly reverts to the mean, the sons of individuals at the top of the human-capital distribution will perform worse than their fathers even if no nepotism is at place. To gauge how much do distributional differences depend on nepotism and how much on mean-reversion, we assume stationarity in the distribution of human capital over all potential scholars. The stationarity assumption and our first set of moments (which identify the intergenertional human capital elasticity β) allow us to uncover the rate of mean-reversion. That is, how different fathers and sons are supposed to look like in the absence of nepotism. Hence, any excess distributional differences, net of reversion to the mean, can be attributed to nepotism. Formally, imposing stationarity implies that the difference in human capital between fathers and sons should follow:

$$h_{i,t+1} = \beta h_{i,t} + (1-\beta)\mu_h + \omega_{i,t+1},$$

where $\omega_{i,t+1}$ is a shock distributed according to $N(0, (1-\beta^2)\sigma_h^2)$. In the absence of nepotism, this differences in human capital would be directly translated into the following differences in publications:

$$y_{i,t} = \max(\kappa, h_{i,t} + \epsilon_{i,t})$$

$$y_{i,t+1} = \max(\kappa, \beta h_{i,t} + (1-\beta)\mu_h + \omega_{i,t+1} + \epsilon_{i,t+1})$$

If the father-son difference in publications is larger than suggested by the previous equations, then an additional force must be in place. A force selecting fathers and sons differently, such that the later can become scholars with lower human capital endowments. In our setting, this additional force is nepotism.

How would our nepotism estimate change in a non-stationary environment? In our setting, it is reasonable to assume that if the human capital distribution is non-stationary, then it *improves* over time. Under this scenario we would expect more sons with higher human capital than their fathers than under stationarity. This implies that, in the absence of nepotism, we would expect virtually no distributional differences in publications between fathers and sons. In extreme scenarios, we would even expect the sons publication's distribution to dominate that of their fathers. Hence, we would need a larger nepotism parameter to reconcile the large observed father-son distributional differences in publications with the small expected differences. In other words, under stationarity, a share of

the father-son distributional differences is attributed to nepotism, and another to mean-reversion. In a non-stationary environment, mean-reversion would explain a lesser share of the father-son distributional differences, and hence, our nepotism estimate would have to be larger. Therefore, under stationarity, our nepotism estimates are conservative, lower-bound estimates.

Evidence. The fact that our (already large) nepotism estimate is a conservative estimate is reassuring. Here we present additional evidence supporting the stationarity assumption, and hence, that our nepotism estimate is not severely downward biased. Ideally, we would show that the mean of the human capital distribution, μ_h, is constant over time for all *potential scholars*. Since we do not observe h, we will focus on trends in our observed human-capital proxy: publications. To evaluate a universe resembling all *potential scholars*, we use the dataset collected by De la Croix (2021b) on 58,251 pre-industrial scholars.

Figure G.2 shows the trend in publications on the intensive margin (top panel). That is, it shows the inverse hyperbolic sine of the number of library holdings, conditional on having at least one publication listed in WorldCat. To calculate trends, we use a kernel-weighted local polynomial regression of publications on a scholar's reference date. The figure shows no trend in the intensive margin of publications, supporting our stationarity assumption.[13]

The bottom panel shows trends on the extensive margin of publications: that is, whether a scholar is listed in WorldCat. The figure shows a U-shaped pattern. Before 1350, the extensive margin of publications is high because of a selection effect: top scholars are more likely to be observed. That said, we have a limited number of observations from this period (36), and hence, it is unlikely that this has a large impact on our aggregate results. Around 1450, when the printing press was introduced, there is a structural break in the extensive margin of publications. There are two reasons to believe that this structural break does not reflect a change in the human capital distribution but a change in the technology for printing and preserving books: The first reason is that the printing press massively increased the diffusion and preservation of scholar's books (Dittmar 2019). This alone could explain the observed trend without resort to changes in the human capital distribution. Formally, this trend is related to our parameter κ, the measurement error on the extensive margin of publications, and not to μ_h, the mean of the human capital distribution among potential scholars. This is supported by our higher estimates for κ for the earlier period (1088–1543) (see Section 5.3). The second reason why it is unlikely that this trend reflects changes in the human capital distribution is because such a change would affect the trends in *both* the extensive and the intensive margin of publications. We only observe a trend in the former, suggesting that the changes are related to improvements in the printing and book-preservation technology. Finally, this increasing trend implies that, around 1450, some sons benefited from the existence of the printing press to publish and preserve their work. In contrast, we are more likely to observe zero-publications for their fathers, whose work was not printed and may have been lost. Correcting for this bias would increase the father-son distributional differences. Hence, it would lead to larger nepotism estimates.

[13] The fluctuations before 1350 are driven by a smaller sample in the earlier periods.

Figure G.2: Trend in log-publications, intensive and extensive margin

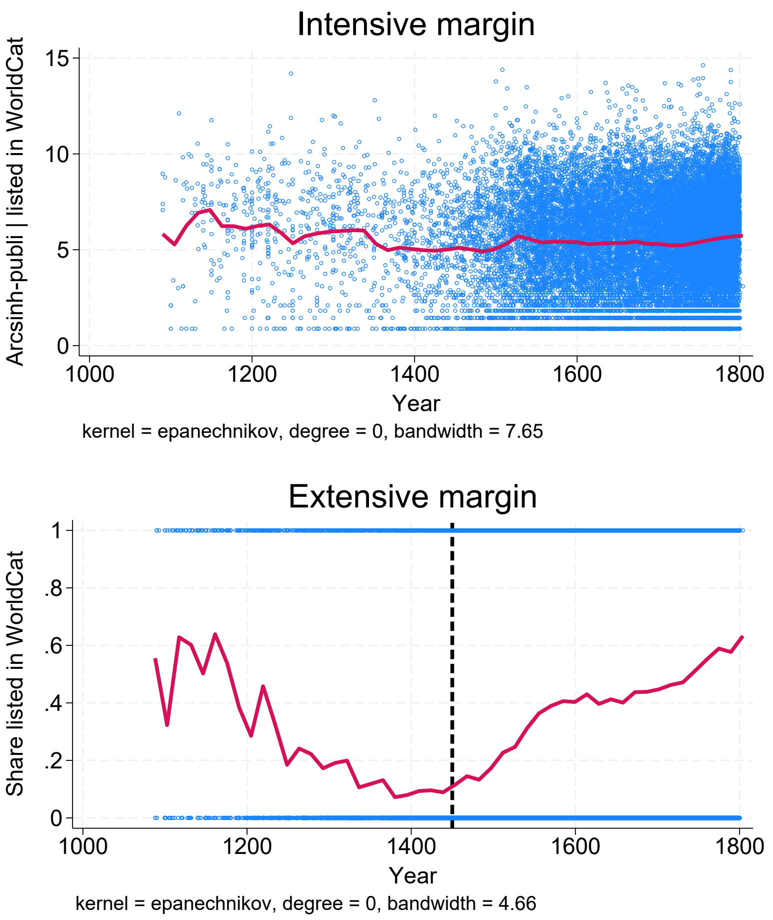

Notes: The sample is all 58,251 scholars in De la Croix (2021b). Trends calculated with a kernel-weighted local polynomial regression. The dashed line is for the introduction of the printing press.

In sum, the De la Croix (2021b) dataset comprising 58,251 scholars shows no trend on the intensive margin of publications. This supports our stationarity assumption for the human capital distribution. On the extensive margin, we find evidence of a structural break around 1450. That said, this is related to the changes brought about by the printing press in terms of book diffusion and preservation, rather than with a change in the human capital distribution.

H Robustness to distributional assumptions

The intergenerational transmission of wealth is often modeled assuming a normal distribution for the initial distribution of wealth $h_{i,t}$ and the idiosyncratic shock $u_{i,t+1}$. How do these distributional assumptions affect our results? Could the large nepotism estimate be a by-product of these distributional assumptions? Here we consider an alternative to normality: drawing shocks from fat-tailed distributions. This distributions give a higher likelihood to the emergence of geniuses, which is appealing in our setting with individuals at the very top of the talent distribution.

Before re-estimating our results, we need to consider two issues: the first concerns the targeted moments, the second the set of feasible fat-tailed distributions. Some of the commonly targeted moments when shocks are normal are not defined when shocks are fat tailed. This is the case of Pearson correlation and of the mean. Hence, if we want to use shocks from fat tailed distributions, we need to target an alternative set of moments ($V_S(p)$). Specifically, we replace the Pearson correlation for the Spearman rank correlation—which remains well-defined with any distribution—and we drop the two means from the targeted moments. We thus have four overidentifying restrictions instead of six. To show that these changes are not crucial for our results, we first conduct our baseline estimation under this new set of moments to define a new benchmark.

Table H.1 presents the results of the new benchmark and compares them to the estimation in the main text ($V(p)$). The Spearman correlations ρ_S are identical to their Pearson counterparts ρ, and all estimates are similar under the two different objectives. In detail, our two main estimates—the intergenerational human capital elasticity, β, and the magnitude of nepotism, γ—are not significantly different when we target the moments in $V(p)$ or in $V_S(p)$. Overall, the table shows that targeting this alternative set of moments does not alter our baseline results, and hence, that we can use them to check the robustness to using fat tailed shocks.

The second issue is the set of feasible fat tailed distributions. We need distributions with closed-form expressions for the density to verify that their shape is preserved (up to scale and shift) under addition. To see why, note that the sum-stable property of the normal distribution implies that its shape remains the same across all generations once transformed by Equation (2), that is, $h_{i,t+1} = \beta h_{i,t} + u_{i,t+1}$. Only its parameters change. This stability property is not a theoretical curiosity. Without it, we lack of coherence in modeling, as the initial distribution of human capital could not be rationalized by the model, its shape having vanished after one period. There are two families of fat-tailed distributions where one can verify that the sum-stable property is satisfied as in the normal distribution: The Cauchy and Levy distribution (Nolan 2003). Here we use the Cauchy distribution, which is fat tailed but, unlike the Levy distribution, is defined over $\mathbb{R}$.

After discussing these two issues, we can now analyze the effect of using fat tailed distributions on our results. Specifically, the theoretical model of human capital transmission with nepotism where shocks are Cauchy is as follows. A potential scholar in generation t of family i is endowed with an unobserved human capital $h_{i,t}$. Human capital follows a Cauchy distribution with location x_h and

Table H.1: Benchmark estimation under different set of moments

Objective:		$V(p)$	$V_S(p)$
**Panel A.** Moments:			
Father-son correlations:			
Pearson, intensive margin	$\rho(y_t, y_{t+1})$	0.375	.
Spearman, intensive margin	$\rho_S(y_t, y_{t+1})$	.	0.375
Grandfather-grandson correlations:			
Pearson, intensive margin	$\rho(y_t, y_{t+2})$	0.234	.
Spearman, intensive margin	$\rho_S(y_t, y_{t+2})$	.	0.265
Distribution means:			
Father mean log-publications	$E(y_t)$	YES	.
Son mean log-publications	$E(y_{t+1})$	YES	.
Remaining distributional moments:		YES	YES
**Panel B.** Identified parameters:			
Intergen. elasticity of human capital	β	0.63	0.70
		(0.04)	(0.04)
Nepotism, %	γ	18.7	19.4
		(1.7)	(1.7)
Mean of human capital distribution	μ_h	1.87	1.25
		(0.47)	(0.65)
SD of human capital distribution	σ_h	4.22	4.43
		(0.20)	(0.24)
SD of shock to publications	σ_e	0.39	0.55
		(0.15)	(0.19)
Threshold of observable publications	κ	2.12	2.00
		(0.14)	(0.13)
Degrees of overidentification		6	4

Notes: τ normalized to 0. S.E. between parentheses obtained by estimating parameters on 200 bootstrapped samples with replacement

scale parameter s_h:

$$h_{i,t} \sim \text{Cauchy}(x_h, s_h)$$

The offspring of this generation, indexed $t+1$, inherit the unobserved human capital endowment under the first-order Markov process in Equation (2). The noise term $u_{i,t+1}$ is an i.i.d. ability shock affecting generation $t+1$, and has now a Cauchy distribution, Cauchy(x_u, s_u).

Human capital is stationary among potential scholars. That is, we assume that, conditional on the model's parameters being constant, the human capital of generations t and $t+1$ is drawn from the same distribution. Formally, $h_{i,t} \sim$ Cauchy(x_h, s_h) and $h_{i,t+1} = \beta h_{i,t} + u_{i,t+1}$ implies $h_{i,t+1} \sim$

Cauchy$(\beta x_h + x_u, |\beta| s_h + s_u)$.[14] Stationarity leads to the following two restrictions:

$$\begin{aligned} x_u &= (1-\beta)x_h \\ s_u &= (1-|\beta|)s_h\,. \end{aligned}$$

Equations (4)-(5) give the publications for fathers, $y_{i,t}$ and sons, $y_{i,t+1}$ in the set of scholar families $\mathbb{P}$. The shocks affecting how human capital translates into publication now follow a fat-tailed distribution: $\epsilon_{i,t}, \epsilon_{i,t+1} \sim \text{Cauchy}(0, s_e)$.

Finally, the magnitude of nepotism, γ, is defined analogously to our baseline model. Formally,

$$\gamma = F_h^{cauchy}(\tau \mid h_{i,t+1} \geq \tau - \nu)\,,$$

where $F^{cauchy}(x;\ x_h, s_h)$ is the (stationary) Cauchy cumulative distribution of human capital with location x_h and scale parameter s_h, and

$$F^{cauchy}(x \mid h_{i,t+1} \geq \tau - \nu) = Prob\left(h_{i,t+1} \leq x \mid h_{i,t+1} \geq \tau - \nu\right)$$

is the corresponding truncated cumulative distribution of sons' human capital in the set of observed scholar families $\mathbb{P}$.

There are three variants to the model of the main text (Model I). One with Cauchy distribution for all shocks (Model II), another with Cauchy distribution for shocks to human capital and Normal distribution for shocks to publications (Model III), and another with Normal distribution for shocks to human capital and Cauchy distribution for shocks to publications (Model IV). We evaluate Models II and III, as they lead to non-normal human capital distribution.

Table H.2 shows the results. The value of the objective to be minimized is an order of magnitude higher when human capital shocks are modeled with a Cauchy (794 vs. 6,613 and 4,940, respectively). In other words, the data cannot be fitted well to a distribution with fat tails. For example, the 50th percentile for the son's publication distribution is 3.4 arcsinh-library holdings in the data (Table D.1), 3.1 in the simulation with the Normal distribution, and 1.2 (Model II) and 0.9 (Model III) in the simulations with the Cauchy. Finally, the nepotism estimates are robust to assuming Cauchy shocks, although the intergenerational elasticity β is not.

In sum, using fat tailed distributions for human capital shocks seems, *a priori*, an appealing alternative to the usual normality assumption. However, fat tailed distributions do not fit the data, which is very normally distributed after all.

[14] Because if $X \sim \text{Cauchy}(x_0, s_0)$ we have $kX + \ell \sim \text{Cauchy}(kx_0 + \ell, |k|s_0)$. And if $Y \sim \text{Cauchy}(x_1, s_1)$, $X + Y \sim \text{Cauchy}(x_0 + x_1, s_0 + s_1)$.

Table H.2: Identified parameters under different model assumptions

Parameter		Model I	Model II	Model III
Intergen. human capital elasticity	β	0.703	0.298	0.437
Nepotism, %	γ	19.35	17.57	25.58
Std. dev. of shock to publications	σ_e	0.553	.	2.259
Scale of shock to publications	s_e	.	0.100	.
Threshold of observable publications	κ	2.002	0.820	0.002
Mean of human capital distrib.	μ_h	1.248	.	.
Location of human capital distrib.	x_h	.	0.847	0.040
Std. dev. of human capital distrib.	σ_h	4.430	.	.
Scale of human capital distrib.	s_h	.	1.094	0.920
Value of objective $V(p)$		793.8	6,613.2	4,940.1

Notes: τ normalized to 0; degrees of overidentification: 4

I Linearity of beta

So far, we assumed that parents with high and low human capital transmit their endowments at the same rate β. This linearity assumption would be violated, e.g., if successful fathers with many publications could spend less time with their children, reducing their human capital transmission systematically. Here we show empirically that, in our setting, the linearity assumption is satisfied.

To do so, we examine the parent-child elasticity of publications in the intensive margin. A large literature derives estimates of β directly from such parent-child elasticities. Here we compare elasticities obtained using OLS vs. estimated non-parametrically. The latter allow elasticities to differ in families with different levels of publications, and hence, with different human capital endowments.

Formally, our OLS elasticity estimates, b^{ols}, are:

$$y_{i,t+1} = c + b^{ols}\, y_{i,t} + e_{i,t+1}\,, \tag{4}$$

where $y_{i,t+1}$ and $y_{i,t}$ are the inverse hyperbolic sine of library holdings for, respectively, sons and fathers with at least one publication in WorldCat. That is, b^{ols} is the publications' elasticity in the intensive margin. In our setting, we can interpret arcsinh-arcsinh specifications as elasticities because the number of library holdings (in levels) of fathers and sons take on large values, with means well-above the threshold proposed by Bellemare and Wichman (2020).[15] This specification assumes that b^{ols} is linear.

Conversely, non-parametric estimates for the publication's elasticity, b^{np}, are:

$$y_{i,t+1} = g(y_{i,t}) + e_{i,t+1}\,, \tag{5}$$

where $g(.)$ does not follow any given parametric form but is derived from the data. Hence, this specification accounts for any polynomial form for $g(.)$, i.e., $g(y_{i,t}) = c + \sum_j b_j^{np}\, y_{i,t}^j$ for all $j \in \mathbb{Z}$. This allows elasticities to be different across families with different levels of publications. The non-parametric elasticity b^{np} corresponds to the marginal effect of $y_{i,t}$, obtained as averages of the derivatives.

Figure I.1 compares OLS and non-parametric elasticity estimates. It shows a scattergram of fathers' (y-axis) and sons' (x-axis) publications, OLS fitted values from eq. (4) (dashed line), and non-parametric fitted values and 95% confidence intervals from eq. (5) (thick red line and grey area). Specifically, the latter plots the smoothed values of a kernel-weighted local polynomial regression of $y_{i,t+1}$ on $y_{i,t}$. To further capture non-linearities, we choose a polynomial of degree one for the smoothing. Finally, note that in this figure the OLS and non-parametric elasticities correspond to the slopes of the plotted lines.

Overall, the figure shows that there is no statistically significant difference between OLS and non-parametric estimates. This is true at all levels of father's publications. For fathers with fewer than 12 arcsinh-publications (more than $\leq 80,000$ in levels), the fitted OLS and non-parametric values are identical. In turn, the parent-child elasticity in publications (i.e., the slope of the lines) is tightly identified around 0.36 for both estimates. At the very top of the distribution, we also do not observe

[15] See footnote 16 in the main text for details.

Figure I.1: Parent-child publications' elasticity (intensive margin), OLS and kernel-weighted local polynomial regression

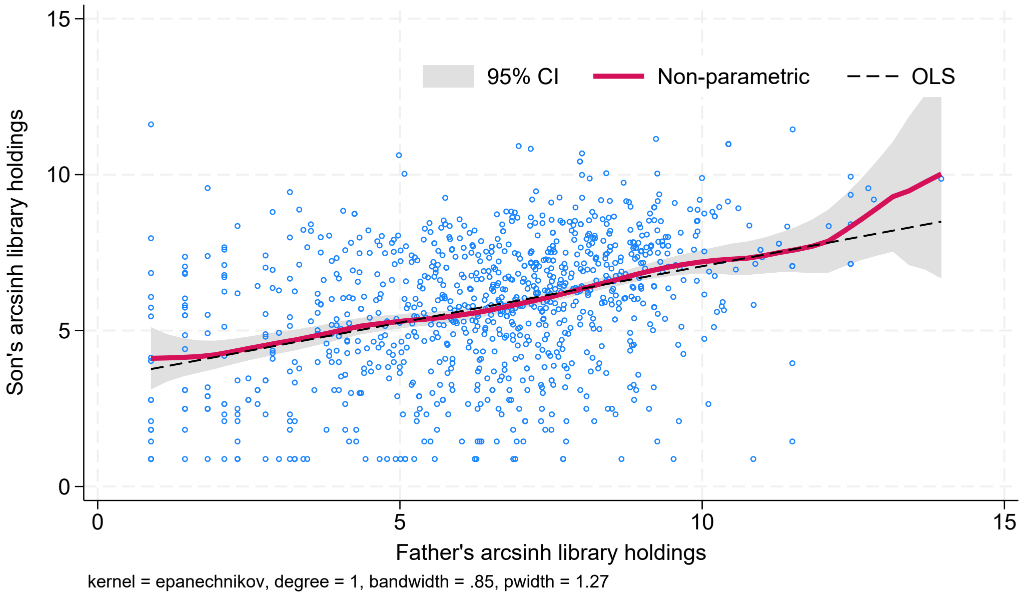

Notes: The sample are fathers and sons with at least one recorded publication.

significant differences between OLS and non-parametric estimates, although the confidence intervals are wider due to fewer number of observations.

Table I.1 confirms this pattern for different historical periods. It shows the OLS (eq. 4) and non-parametric (eq. 5) elasticities for all families (row 1); for families before the Scientific Revolution (row 2); during the Scientific Revolution (rows 3 and 4); and during the Enlightenment (row 5). For all periods, the OLS and non-parametric estimates are almost identical.

Table I.1: Parent-child publications' elasticity (intensive margin), OLS and Non-parametric estimates

	OLS [1]		Non-parametric [2]		
All	0.36***	(0.03)	0.36***	(0.03)	N=982
Pre-Scientific Revolution (1088–1543)	0.06	(0.11)	0.06	(0.10)	N=83
Scientific Revolution (1543–1632)	0.36***	(0.07)	0.38***	(0.07)	N=196
Scientific Revolution (1632–1687)	0.32***	(0.05)	0.30***	(0.07)	N=271
Enlightenment (1688–1800)	0.44***	(0.04)	0.44***	(0.04)	N=432

Note: The sample are fathers and sons with at least one publication; SE in parenthesis; Non-parametric SE obtained with 1,000 bootstrapped replications; ***p<.01,** p<.05,* p<.1

Altogether, we find identical elasticities using OLS and non-parametric techniques. This suggests that the parent-child elasticity of publications is linear. In other words, it is identical for parents with high and low publications. This lends credence to the assumption that human capital endowments are transmitted at the same rate β by parents with high and low human capital endowments.

J Heterogeneity in publication thresholds

The parameter κ is the minimum number of publications needed to observe a scholar's work in modern libraries. So far, we assumed that κ is the same for fathers and sons. An alternative is to assume that the threshold is lower for sons: the work of a famous scholar's son may capture the attention of publishers and librarians more easily—even if it is of lower quality.

Here we examine the robustness of our results to this alternative assumption. We define the sons' threshold as κ_s, possibly lower than the father's threshold κ_f and estimate the corresponding model in Table J.1. We find that the constraint $\kappa_s \leq \kappa_f$ is saturated: our estimated κ_s and κ_f are almost identical. Hence, our estimation results are unchanged: we find very similar intergenerational human capital transmission β (0.634 vs. 0.626) and percentage of nepotic sons γ (18.74 vs. 19.04%).

TABLE J.1: Results under alternative assumptions for κ

Parameter		benchmark	different κ's
Intergenerational elasticity of human capital	β	0.634	0.626
Nepotism, %	γ	18.74	19.04
Mean of human capital distribution	μ_h	1.865	1.846
Std. deviation of human capital distribution	σ_h	4.219	4.251
Std. deviation of shock to publications	σ_e	0.393	0.415
Threshold of observable publications - all	κ	2.121	.
Threshold of observable publications - fathers	κ_f	.	2.118
Threshold of observable publications - sons	κ_s	.	2.118

Notes: τ normalized to 0; degrees of overidentification: 6

K Alternative measures of publications

In the main text, we defined publications as the number of library holdings in modern libraries by or about each scholar. This includes all imprints/editions/copies of books, volumes, issues, or documents which a scholar wrote that are available in WorldCat libraries today. It also includes library holdings about his work written by a different author. We chose this measure of publications as our baseline measure because it captures two important characteristics of a scholar's research: its size and its relevance for today in a manner akin to modern citation data. Although we believe both characteristics to be important, it is interesting to examine the robustness of our results to measuring only the size of a scholar's work. To do so, here we consider two alternative measure of publications: The first measure is the number of library holdings written by each scholar, i.e., omitting library holdings about his work written by a different author. The second measure is the number of unique works by or about each scholar instead of the total number of library holdings.

Table K.1 provides the empirical moments for our baseline measure (the arcsinh of library holdings by and about each author) in column [1], and for our alternative measures (the arcsinh of library holdings by each author and the arcsinh of unique works) in columns [2] and [3]. Panel A shows that the inter-generational correlations are very similar on the intensive margin across these three measures. On the extensive margin, the correlation is equal by construction. Overall, this indicates that the high inter-generational elasticity (Fact 1) is visible on the library holdings by and about each author, on the library holdings by each author, and on the number of unique works.

Panel B shows the moments characterizing father-son distributional differences. The levels are different by construction: the library holdings written by each author and, especially, the number of unique works are equal or smaller that the total number of library holdings written by and about each author. That said, the properties of the distribution and the father-son distributional differences (Fact 2) are robust to using different publications' measures. To see this, note that the father's median, mean, 75th and 95th quantile are substantially higher than their sons' in the three measures. To further show that the properties of the fathers' and sons' distribution are similar, Table K.2 shows quantile ratios. The median/Q75 ratio, the median/Q95 ratio, and the median/mean ratio are similar for fathers and sons independently of whether one uses library holdings by and about each author, library holdings by each author, or unique works as the measure of research output.

Finally, Table K.3 re-estimates our model targeting the moments defined with library holdings by and about each author (column 1), with library holdings by each author (column 2), and with unique works (column 3). Our estimates for the intergenerational elasticity of human capital and for nepotism are remarkably similar across specifications. Specifically, excluding publications about a scholar's work written by a different author leads to a β-estimate of 0.62, very similar to our baseline estimate that includes them (0.63). The nepotism estimate, γ, is idential across measures, suggesting that 18.7% of scholars' sons were nepotic. Similarly, using the number of unique works, we find a β of 0.61 and a nepotism estimate of 18.8.

Altogether, the evidence presented in this appendix suggests that our main estimates for the in-

tergenerational elasticity of human capital and for nepotism are robust to how we measure a scholar's research output. Specifically, our results are not a byproduct of whether our definition of publications includes work written by a different author or is based on library holdings instead of unique works.

TABLE K.1: Targeted moments with alternative measures of publications

	[1]	[2]	[3]
	Library holdings by & about author	Library holdings by author	Unique works
A. Intergenerational correlations			
Father-son, int. margin	0.375	0.366	0.357
Father-son with zero pubs.	0.211	0.211	0.211
Grandfather-grandson, int. margin	0.234	0.212	0.230
B. Father-son distributional differences			
Fathers with zero pubs.	0.288	0.290	0.290
Sons with zero pubs.	0.384	0.384	0.384
Fathers median	5.075	4.238	3.801
Sons median	3.402	2.615	2.440
Fathers Q75	7.370	6.300	5.762
Sons Q75	6.413	5.415	4.950
Fathers Q95	9.425	8.213	7.568
Sons Q95	8.537	7.306	6.748
Fathers mean	4.456	3.752	3.441
Sons mean	3.477	2.893	2.664

TABLE K.2: Comparison of distributions

	[1]	[2]	[3]	
		Library holdings by & about author	Library holdings by author	Unique works
Q50/Q75	Fathers	0.69	0.67	0.66
Q50/Q75	Sons	0.53	0.48	0.49
Q50/Q95	Fathers	0.54	0.52	0.50
Q50/Q95	Sons	0.40	0.36	0.36
Q50/mean	Fathers	1.14	1.13	1.10
Q50/mean	Sons	0.98	0.90	0.92

Table K.3: Identified parameters with alternative measures of publications

		[1]	[2]	[3]
		Library holdings by & about author	Library holdings by author	Unique works
IGE human capital	β	0.63	0.62	0.61
Nepotism, %	γ	18.7	18.7	18.8
Mean human capital	μ_h	1.87	1.65	1.60
SD human capital	σ_h	4.22	3.56	3.37
SD publications's shock	σ_e	0.39	0.29	0.20
Threshold publications	κ	2.12	1.82	1.74

Notes: τ normalized to 0; degrees of overidentification: 6

L Longevity

Longevity is an important factor for the number of publications of scholars. In our setting, scholars' fathers may have lived longer than scholars' sons. The reason is that, by construction, the former are recorded in our data conditional on living until they have a child, while the latter are recorded even if they die early after their nomination. In our sample of scholars with known birth and death year, the mean longevity is 67.65 (s.e 0.32) for fathers and 61.67 (s.e. 0.44) for sons. Here we show that this differential longevity does not affect our results.

To do so, we adjust the son's distributional moments accounting for the 5.98 year father-son gap in longevity. We do this in two steps. First, we calculate the marginal effect of living one additional year on the proportion of sons with zero publications and on the mean, median, 75th, and 95th percentile of the sons' log-publications. Second, we adjust the baseline distributional moments for sons by adding the marginal effects above times 5.98; the differential longevity between fathers and sons. That is, we calculate what the sons' distributional moments would look like if they had, on average, lived as long as fathers of scholars.

Formally, we first estimate the following equation by OLS:

$$y_{i,t+1} = \alpha + \delta(mean) \cdot L_{i,t+1} + e_{0\ i,t+1}\,, \tag{6}$$

where i indicates families of scholars and $t+1$ that the observation corresponds to a scholar's son; $y_{i,t+1}$, is the inverse hyperbolic sine of the number of library holdings; and $L_{i,t+1}$ is the son's longevity, in years. Hence, $\delta(mean)$ captures the marginal effect of one additional year of life on the sons' arcsinh-publications. Estimating $\delta(mean)$ by OLS allows to understand this relationship for the *average* son.

We calculate analogously $\delta(zeros)$, the marginal effect on the proportion of sons with zero publications. That is, we estimate 6 by OLS where the dependent variable, $y_{i,t+1}$, is an indicator equal to one if a son had zero publications.

Next, we run a simultaneous-quantile regression to estimate the relation between longevity and publications at other distributional moments than the mean. Formally, we estimate:

$$Q_{y_{i,t+1}}(q|L_{i,t+1}) = \alpha_i + \delta(q) \cdot L_{i,t+1}\,, \tag{7}$$

where q is the quantile of interest; $\delta(Q50)$, $\delta(Q75)$, and $\delta(Q95)$ are the marginal effect of living one additional year on the median, 75th and 95th percentile of the sons' publication distribution; are all coefficients are estimated simultaneously

Table L.1 presents the corresponding estimates. Column [1] confirms that longevity is important for publications. One additional year of life is associated with an increase of 0.023 arcsinh-publications. Hence, if sons lived as long as fathers, their mean arcsinh-publications would increase, on average, by $5.98 \times 0.023 = 0.138$. Column [2] shows the corresponding marginal effect for the proportion of sons with zero publications. Note that this marginal effect is small and implies that, if sons lived as long as fathers, their probability to die without ever publishing would be reduced by $5.98 \times 0.0015 = 0.00897$, or 0.897 percentage points of a sample mean of 38 percent. This suggests

that the high proportion of sons with zero publications is not a by-product of sons dying early after their nomination. This is important as our identification of nepotism partially hinges on father-son distributional differences at the bottom of the distribution. Finally, columns [3] to [5] show that one additional year of life is associated with an increase of 0.03 arcsinh-publications at the median and 75th percentile, and with an increase of 0.014 arcsinh-publications at the 95th percentile of the sons' publications distribution. Hence, if sons lived as long as fathers on average, their arcsinh-publications would increase by $5.8 \times 0.03 = 0.179$ at the median and 75th percentile; and by $5.8 \times 0.014 = 0.084$ at the 95th percentile.

TABLE L.1: The effect of Longevity on son's distributional moments

	[1]	[2]	[3]	[4]	[5]
	OLS	OLS	simultaneous-quantile regression		
	$\partial(mean)$	$\partial(zeros)$	$\partial(Q50)$	$\partial(Q75)$	$\partial(Q95)$
Longevity (years)	0.023***	-0.0015**	0.031***	0.030***	0.014**
	(0.005)	(0.0007)	(0.007)	(0.005)	(0.007)
Observations	1,329	1,329	1,329	1,329	1,329

Note: The sample is scholars' sons with known birth and death year;$^{***}p<.01$,$^{**}p<.05$,$^{*}p<.1$

Finally, Table L.2 shows the adjusted sons' distributional moments. Column [1] shows the baseline moments and column [2] the adjusted moments if scholars' sons had lived as long as scholars' fathers. The adjusted moments are $m + \partial(m) \times 5.98$; where m is the baseline value, $\partial(m)$ the marginal effect of longevity at moment m, and 5.98 the father-son differential longevity.

The baseline and adjusted moments are very similar. The proportion of sons with zero publications (0.38) is almost not altered by adjusting for the fathers-sons longevity differential (0.37). The mean, median, 75th and 95th percentile of the sons' log-publications are larger when we impute the same longevity to sons and fathers. For example, if sons lived as long as fathers on average, their mean arcsinh-publications would have been 3.61 instead of 3.48—which corresponds to an increase of 0.14 arcsinh-publications. That said, the adjusted distributional moments are consistent with *Fact 2*. After accounting for longevity differentials, the publication's distribution of fathers first order stochastically dominates that of sons. On the bottom of the distribution, 30% of fathers and 37% of sons had zero publications, even after accounting for longevity differentials. These distributional differences are also visible at the mean, median, 75th and 95th percentile. For example, the average father had 4.5 arcsinh-publications (45 in levels), more than twice as much as the average son (18.5 in levels) even after accounting for longevity differentials. The father-son differences at the median are reduced by 0.18 arcsinh-publications after adjusting for longevity, but the median father still published substantially more more than the median son. Importantly, this implies that, after adjusting for longevity differentials, the father-son distributional differences are relatively larger at the bottom of the distribution than at the mean or at the median.

Altogether, the evidence suggests that longevity affects publications, but that father-son longevity differences do not explain away father-son distributional differences (*Fact 2*). This shows that our

estimates for nepotism and the intergenerational human capital elasticity are not driven by differences in longevity.

Table L.2: Distributional moments adjusted for longevity differentials

		Baseline [1]	Adjusted [2]	Difference [1]-[2]
Fathers with zero pubs.	$\Pr(y_t=0)$	0.29	.	.
Sons with zero pubs.	$\Pr(y_{t+1}=0)$	0.38	0.37	0.01
Fathers median	$Q50(y_t)$	5.08	.	.
Sons median	$Q50(y_{t+1})$	3.40	3.58	-0.18
Fathers 75th percentile	$Q75(y_t)$	7.37	.	.
Sons 75th percentile	$Q75(y_{t+1})$	6.41	6.59	-0.18
Fathers 95th percentile	$Q95(y_t)$	9.43	.	.
Sons 95th percentile	$Q95(y_{t+1})$	8.54	8.62	-0.08
Fathers mean	$E(y_t)$	4.46	.	.
Sons mean	$E(y_{t+1})$	3.48	3.61	0.14

M Fertility differentials in academia

This appendix examines the sensitivity of Fact 2—i.e., that the publication's distribution of fathers first order stochastically dominates (FOSD) that of sons—and of our nepotism estimates to fertility differentials between scholars.

As for fertility in general, we unfortunately do not have data on complete families of the professors in the sample. Baudin and De la Croix (2023), however, reconstruct families of professors from Northern Europe, for whom there are many genealogies available in the crowdsourced genealogical websites such as geni.com. That paper shows that the differential fertility between more and less successuful scholars changes over time. From 1625 to 1700, scholars who were more successful at publishing also had more children. From 1700 to 1800, the relationship is reversed, and more successful scholars have fewer children than scholars who published less. These fertility differentials are small, around 0.1-0.2 sons for scholars above vs. below the median in terms of publications. Hence, it is unlikely that the differential fertility in favor of more successful scholars is large enough over our entire sample period to explain away our FOSD fact or our nepotism estimates. That said, because our aim is to study the transmission of upper tail human capital within academia, we believe that conditioning on individuals in our sample being in academia is the right choice.

As for fertility in the sense of number of kids in academia, Table M.1 presents the distribution of parities in our sample. That is, it shows the number of fathers (and their mean publications) by the number of sons they had who entered in academia.

TABLE M.1: Distribution of parities

parity x	$x = 1$	$x = 2$	$x = 3$	$x = 4$
No. fathers with x children in academia	1320	165	27	3
Mean arcsinh-publications of fathers	4.25	4.4	5.03	6.28
S.E. of the mean	0.1	0.26	0.81	3.16

We have 1,320 fathers with one child in academia, 165 fathers with two children in academia, 27 fathers with 3 children in academia, and 3 fathers with 4 children in academia. The fathers with one and two academic children have similar publications (4.25 and 4.4 respectively). The (few) fathers with more than two children in academia seem more successful in publishing, but the difference is not statistically significant. It is unlikely that these 30 very successful fathers, or even the 195 fathers with more than one child, would bias our nepotism estimates, as they represent a small proportion of our sample and the differences in mean publications are not that large. Nevertheless, to examine this, we re-estimate the parameters of our model excluding them, i.e., excluding fathers with more than one child in academia.

The results are presented in Table M.2. Reassuringly, the estimations are very similar. Specifically, our nepotism estimate (γ) and our intergenerational human capital elasticity (β) are almost identical when we include or not fathers with more than one child in academia. A Clogg et al. test cannot

TABLE M.2: Robustness to fertility differentials within academia

		All	Scholars with one child in academia	Difference
		[1]	[2]	[3]
IGE human capital	β	0.63 (0.04)	0.68 (0.05)	0.05 [0.435]
Nepotism, %	γ	18.7 (1.74)	19.4 (2.13)	0.70 [0.799]
Mean human capital	μ_h	1.87 (0.47)	1.41 (0.61)	0.46 [0.733]
SD human capital	σ_h	4.22 (0.20)	4.36 (0.25)	0.14 [0.662]
SD publications' shock	σ_e	0.39 (0.15)	0.47 (0.18)	0.08 [0.550]
Threshold publications	κ	2.12 (0.14)	2.06 (0.15)	0.06 [0.770]

Notes: SE in parenthesis from 200 bootstrapped samples with replacement; degrees of overidentification: 6; Column [3] shows the difference (col. [2]-[1]) and the corresponding p-value based on Clogg, Petkova, and Haritou (1995)'s test.

reject the null that and the estimates are equal with a p-value of 0.435 for β, and of 0.799 for γ. Hence, we can conclude that the presence of fathers having multiple children in academia does not bias the benchmark results.

References

Bayerische Akademie der Wissenschaften. 2022. "Deceased members." https://badw.de/en/community-of-scholars/deceased.html.

Accademia delle Scienze di Torino. 1973. "Dizionario biografico dei Soci dell'Accademia." Torino.

Addy, George M. 1966. *The Enlightenment in the University of Salamanca*. Durham NC: Duke University Press.

Adermon, Adrian, Mikael Lindahl, and Daniel Waldenström. 2018. "Intergenerational Wealth Mobility and the Role of Inheritance: Evidence from Multiple Generations." *The Economic Journal* 128 (612): F482–F513.

Albrecht, Helmuth, and Bettina Gundler. 1986. *Catalogus professorum der Technischen Universität Carolo-Wilhelmina zu Braunschweig*. Braunschweigischen Hochschulbundes.

Alcocer Martinéz, Mariano. 1918. *Historia de la Universidad de Valladolid – Expendientes de provisiones de catedras*. Valladolid: Imprenta Castellana.

Anderson, Peter John. 1893. *Lists of Officers, University and King's College: Aberdeen, 1495-1860*. Aberdeen: Aberdeen University Press.

———. 1898. Records of Marischal College and University.

Andreev, A Yu, and DA Tsygankov. 2010. *Imperatorskiy Moskovskiy universitet, 1755-1917: entsiklopedicheskiy slovar [Imperial Moscow University, 1755-1917: Encyclopedic Dictionary]*. Moscow: ROSSPeN.

Anonymous. 1896. *Complete List of the Members & Officers of the Manchester Literary and Philosophical Society*. Manchester: 36, George Street.

Antonetti, Guy. 2013. *Les professeurs de la faculté des droits de Paris 1679-1793*. Paris: Editions Pant'éon-Assas.

Appelt, Joachim, and Annett Wulkow. 2022. "Professoren (bis 1945)."

Arteaga, Enrique Esperabé. 1917. *Historia pragmática é interna de la Universidad de Salamanca: maestros y alumnos más distinguidos*. Imp. y Lib. de Francisco Núñez Izquierdo.

Astro.uu.se. 2011. "History of astronomy in Uppsala."

Astruc, Jean. 1767. *Mémoires pour servir à l'histoire de la faculté de médecine de Montpellier*. P.-G. Cavelier.

Attinger, Victor, Marcel Godet, and Heinrich Türler. 1928. *Dictionnaire historique et biographique de la Suisse*. Neuchâtel: Administration du Dictionnaire historique et biographique de la Suisse.

Barbot, Jules. 1905. *Les chroniques de la faculté de médecine de Toulouse*. Toulouse: Dirion.

Barjavel, Casimir François Henri. 1841. *Dictionnaire historique, biographique et bibliographique du département de Vaucluse*. Devillaris.

Baudin, Thomas, and David De la Croix. 2023. "The Emergence of the Child Quantity-Quality Tradeoff – insights from early modern academics." unpublished.

Bauer, Johannes Joseph. 1957. *Zur Frühgeschichte der Theologischen Fakultät der Universität Freiburg (1460-1620)*. Freiburg: Albert.

BBAW. 2022. Mitglieder der Berliner Akademien – chronologisch. Berlin-Brandenburgischen Akademie der Wissenschaften.

Beaune, Henri, and Jules d'Arbaumont. 1870. *Les Universités de Franche-Comté: Gray, Dole, Besançon: documents inédits publiés avec une introduction historique*. J. Marchand.

Behrman, Jere R, and Mark R Rosenzweig. 2002. "Does increasing women's schooling raise the schooling of the next generation?" *American Economic Review* 92 (1): 323–334.

Belin, Ferdinand. 1905. *Histoire de l'ancienne université de Provence, ou Histoire de la fameuse univesité d'Aix: d'après les manuscrits et les documents originaux*. Paris: A. Picard et fils.

Bellemare, Marc F., and Casey J. Wichman. 2020. "Elasticities and the Inverse Hyperbolic Sine Transformation." *Oxford Bulletin of Economics and Statistics* 82 (1): 50–61.

Benzing, Josef. 1986. *Verzeichnis der Professoren der alten Univerität Mainz*. Mainz: Universitätsbibliothek Johannes Gutenberg-Unviersität.

Berger-Levrault, Oscar. 1890. *Catalogus professorum Academiarum et Universitatum alsaticarum XVI-XVIII seculi*. Impr. de Berger-Levrault.

Berry, Henry Fitz-Patrick. 1915. *A history of the Royal Dublin Society*. Longmans, Green and Company.

Bimbenet, Jean Eugène. 1853. *Histoire de l'Université de lois d'Orléans*. Dumoulin.

Black, Sandra E., and Paul J. Devereux. 2011. "Recent developments in intergenerational mobility." In *Handbook of Labor Economics*, edited by A. Orley and C. David, 1487–1541. Oxford: Elsevier.

Black, Sandra E, Paul J Devereux, Petter Lundborg, and Kaveh Majlesi. 2019, 07. "Poor Little Rich Kids? The Role of Nature versus Nurture in Wealth and Other Economic Outcomes and Behaviours."

Black, Sandra E, Paul J Devereux, and Kjell G Salvanes. 2005. "Why the apple doesn't fall far: Understanding intergenerational transmission of human capital." *American Economic Review* 95 (1): 437–449.

Blasutto, Fabio, David De la Croix, and Mara Vitale. 2021. "Scholars and Literati at the Academy of the Ricovrati (1599–1800)." *Repertorium Eruditorum Totius Europae* 3:51–63.

Boissonade, Prosper. 1932. *Histoire de l'université de Poitiers: passé et présent (1432-1932)*. Poitiers: Imprimerie moderne, Nicolas, Renault & cie.

Borgeaud, Charles. 1900. *Histoire de l'Université de Genève: L'Acadamie de Calvin, 1559-1798*. Geneva: Georg & Company.

Borgerhoff Mulder, Monique, Samuel Bowles, Tom Hertz, Adrian Bell, Jan Beise, Greg Clark, Ila Fazzio, Michael Gurven, Kim Hill, Paul L Hooper, et al. 2009. "Intergenerational wealth transmission and the dynamics of inequality in small-scale societies." *Science* 326 (5953): 682–688.

Bourchenin, Pierre Daniel. 1882. *Étude sur les académies protestantes en France au XVIe et au XVIIe siècle*. Paris: Grassart.

Boutier, Jean. 2017. "Accademia Fiorentina - liste des membres." EHESS, Marseilles.

———. 2018. "Académie des inscriptions et belles lettres, 1663-1793 (liste des membres)." Excel spreadsheet.

Brants, Victor. 1906. *La faculté de droit de l'Université de Louvain à travers cinq siècles <1426-1906>*. Louvain: Ch. Peeters.

Braun, Sebastian Till, and Jan Stuhler. 2018. "The Transmission of Inequality Across Multiple Generations: Testing Recent Theories with Evidence from Germany." *The Economic Journal* 128 (609): 576–611.

Brizzi, Gian Paolo. 2001. *L'antica Università di Fermo*. Milano-Fermo, Silvana.

Brun-Durand, Justin. 1900. *Dictionnaire biographique et biblio-iconographique de la Drôme, contenant des notices sur toutes les personnes de ce département qui se sont fait remarquer par leurs actions ou leurs travaux, avec l'indication de leurs ouvrages et de leurs portraits*. Grenoble: H. Falque et F. Perrin.

Burtchaell, George Dames, and Thomas Ulick Sadleir. 1935. *Alumni dublinenses: a register of the students, graduates, professors and provosts of Trinity College in the University of Dublin (1593-1860)*. A. Thom & Company, Limited.

Calcaterra, Carlo. 1943. *Le adunanze della patria società letteraria*. Societa Editrice Internazionale.

Canella Secades, Fermin. 1873. *Historia de la Universidad de Oviedo y noticias de los establecimientos de enseñanza de su distrito*. Oviedo: Eduardo Uria.

Capeille, Jean. 1914. *Dictionnaire de biographie rousillonnaises*. J. Comet.

Carmignani, Paul. 2017. *L'Université de Perpignan: L'une des plus anciennes universités d'Europe*. Presses universitaires de Perpignan.

Casellato, Sandra, and Luciana Sitran Rea. 2002. *Professori e scienziati a Padova nel Settecento*. Treviso: Antilia.

Catoire, Guillaume, Valentine Debois, David de la Croix, Julie Duchêne, Maximilian Ganterer, and Mara Vitale. 2021. "Scholars and Literati at the Universitas Lovaniensis (1425–1797)." *Repertorium Eruditorum Totius Europae* 4:53–66.

Chenon, Emile. 1890. *Les anciennes Facultés des droits de Rennes (1735-1792)*. H. Caillière.

Chetty, Raj, Nathaniel Hendren, Patrick Kline, and Emmanuel Saez. 2014. "Where is the land of opportunity? The geography of intergenerational mobility in the United States." *The Quarterly Journal of Economics* 129 (4): 1553–1623.

Clark, Gregory. 2015. *The son also rises: Surnames and the history of social mobility*. Princeton University Press.

Clark, Gregory, and Neil Cummins. 2014. "Surnames and social mobility in England, 1170–2012." *Human Nature* 25 (4): 517–537.

———. 2015. "Intergenerational Wealth Mobility in England, 1858–2012: Surnames and Social Mobility." *The Economic Journal* 125 (582): 61–85.

Classen, Timothy J. 2010. "Measures of the intergenerational transmission of body mass index between mothers and their children in the United States, 1981-2004." *Economics and Human Biology* 8 (1): 30–43.

Clogg, Clifford C, Eva Petkova, and Adamantios Haritou. 1995. "Statistical methods for comparing regression coefficients between models." *American journal of sociology* 100 (5): 1261–1293.

Colagrossi, Marco, Beatrice d'Hombres, and Sylke V Schnepf. 2019. "Like (Grand) Parent, like Child? Multigenerational Mobility across the EU." IZA Discussion Paper.

Collado, M Dolores, Ignacio Ortuno-Ortin, and Jan Stuhler. 2018. "Kinship correlations and intergenerational mobility." Universidad Carlos III de Madrid.

Collège de France. 2018. "Liste des professeurs du Collège de France depuis 1530." PDF document.

Collinet, Paul. 1900. *L'ancienne Faculté de droit de Douai (1562-1793)*. Lille: Travaux et memoires de l'université de Lille.

Conrad, Ernst. 1960. “Die Lehrstühle der Universität Tübingen und ihre Inhaber (1477-1927).” Eberhard Karls Universität Tübingen.

Corak, Miles. 2006. “Do poor children become poor adults? Lessons from a cross-country comparison of generational earnings mobility.” In *Dynamics of inequality and poverty*, 143–188. Emerald Group Publishing Limited.

Čornejová, Ivana, and Anna Fechtnerová. 1986. *Životopisný slovnık Pražské Univerzity: Filozofická a Teologická Fakulta 1654 - 1773*. 1. vyd. Praha: Univ. Karlova.

Courteault, Paul. 1912. “Listes des membres: 1712-1912.” *Actes de l'Académie nationale des sciences, belles-lettres et arts de Bordeaux*, pp. 327–357.

Courtenay, William J. 1999. *Parisian scholars in the early fourteenth century: a social portrait*. Volume 41 of *Cambridge Studies in Medieval Life and Thought: Fourth Series*. Cambridge: Cambridge University Press.

Coutts, James. 1909. *A History of the University of Glasgow: From its Foundation in 1451 to 1909*. Glasgow: J. Maclehose and Sons.

Curi, Vincenzo. 1880. *L'Università degli Studi di Fermo: notizie storiche*. Ancona, Ernesto Aurelj.

Dahlgren, Erik Wilhelm. 1915. *Kungl. Svenska vetenskapsakademien: personförteckningar, 1739-1915*. Almqvist & Wiksells boktr.

Da-Rio, Girolamo. 1828. *Giornale dell'Italiana letteratura*. Padova: Tipographia del Seminario.

De la Croix, David. 2020. “Scholars at the Royal Society of Sciences of Montpellier (1706–1793).” *Repertorium Eruditorum Totius Europae* 1:33–39.

———. 2021a. “Scholars and Literati at the Royal College in Paris (1530–1800).” *Repertorium Eruditorum Totius Europae* 1:19–24.

———. 2021b. “Scholars and Literati in European Academia before 1800.” *Repertorium Eruditorum Totius Europae* 5:35–41.

De la Croix, David. 2022. “Scholars and Literati at the University of Strasbourg (1621–1795).” *Repertorium Eruditorum Totius Europae* 8:7–15.

De la Croix, David. 2022. “Literati at the Académie Française (1635-1793).” *Repertorium Eruditorum Totius Europae* 6:17–23.

———. 2023. “Scholars and Literati at the University of Zamość (1594–1784).” *Repertorium Eruditorum Totius Europae* 10:31–37.

De la Croix, David, and Elise Delvaux. 2022a. “Scholars and Literati at the Academy of Sciences and Arts of Toulouse (1729-1793).” *Repertorium Eruditorum Totius Europae* 6:51–58.

———. 2022b. “Scholars and Literati at the Academy of Sciences, Letters and Arts of Besançon (1752-1793).” *Repertorium Eruditorum Totius Europae* 7:39–45.

———. 2022c. “Scholars and Literati at the Academy of Sciences, Letters and Arts of Clermont-Ferrand (1747–1793).” *Repertorium Eruditorum Totius Europae* 8:27–32.

———. 2023a. “Scholars and Literati at the Academy of Sciences and Arts of Dijon (1725-1793).” *Repertorium Eruditorum Totius Europae* 11:17–24.

———. 2023b. “Scholars and Literati at the Philosophical Society & Royal Society of Edinburgh (1731-1800).” *Repertorium Eruditorum Totius Europae* 10:1–7.

De la Croix, David, and Fanny Dock. 2021. “Scholars and Literati at the Royal Academy of Sciences, Letters and Arts of Bordeaux (1712-1793).” *Repertorium Eruditorum Totius Europae* 4:9–17.

De la Croix, David, and Mehrdaad Doraghi. 2021. "Scholars and Literati at the Saint Petersburg Academy of Sciences (1724–1800)." *Repertorium Eruditorum totius Europae* 5:17–26.

De la Croix, David, and Julie Duchêne. 2021. "Scholars and Literati at the Royal Spanish Academy (1713–1800)." *Repertorium Eruditorum Totius Europae* 3:11–17.

De la Croix, David, and Julie Duchêne. 2021. "Scholars and Literati at the "Mersenne" Academy (1635–1648)." *Repertorium Eruditorum Totius Europae* 2:7–12.

De la Croix, David, Thomas Eisfeld, and Maximilian Ganterer. 2021. "Scholars and Literati at the Royal Prussian Academy of Sciences (1700–1800)." *Repertorium Eruditorum Totius Europae* 3:1–9.

De la Croix, David, and Alice Fabre. 2019. "A la découverte des professeurs de l'ancienne université d'Aix, de ses origines á 1793." *Annales du midi* 131:379–402.

———. 2021a. "Scholars and Literati at the University of Aix (1409–1793)." *Repertorium Eruditorum Totius Europae* 4:35–44.

———. 2021b. "Scholars and Literati at the University of Valence (1452–1763)." *Repertorium Eruditorum Totius Europae* 2:13–20.

———. 2022. "Scholars and Literati at the University of Toulouse (1229–1793)." *Repertorium Eruditorum Totius Europae* 8:53–63.

De la Croix, David, and Maximilian Ganterer. 2020. "Scholars and Literati at the Danzig Research Society (1743–1800)." *Repertorium Eruditorum Totius Europae* 1:49–54.

De la Croix, David, Tifenn Gualandris, and Mara Vitale. 2023. "Scholars and Literati at the University of Coimbra (1290–1800)." *Repertorium Eruditorum Totius Europae* 9:49–57.

De la Croix, David, and Hugo Jay. 2021. "Scholars and Literati at the Old University of Aberdeen (1495–1800)." *Repertorium Eruditorum Totius Europae* 4:27–34.

De la Croix, David, and Soraya Karioun. 2021a. "Scholars and Literati at the University of Pont-à-Mousson (1572–1768)." *Repertorium Eruditorum Totius Europae* 2:1–6.

———. 2021b. "Scholars and Literati at the University of Valladolid (1280–1800)." *Repertorium Eruditorum Totius Europae* 1:11–18.

De la Croix, David, and Panagiotis Mytilinaios. 2022a. "Scholars and Literati at the Royal Society (1660–1800)." *Repertorium Eruditorum Totius Europae* 7:11–19.

———. 2022b. "Scholars and Literati at the University of Dole (1422–1691)." *Repertorium Eruditorum Totius Europae* 6:43–50.

De la Croix, David, and Gaia Spolverini. 2021. "Scholars and Literati at the University of Macerata (1540–1800)." *Repertorium Eruditorum Totius Europae* 2:39–45.

———. 2022. "Scholars and Literati at the University of Cracow (1364–1800)." *Repertorium Eruditorum Totius Europae* 6:35–42.

De la Croix, David, and Robert Stelter. 2020. "Scholars and Literati at the University of Jena (1558–1800)." *Repertorium Eruditorum Totius Europae* 1:25–32.

De la Croix, David, and Robert Stelter. 2021. "Scholars and Literati at the University of Gießen (1607–1800)." *Repertorium Eruditorum Totius Europae* 2:31–38.

De la Croix, David, and Robert Stelter. 2021a. "Scholars and Literati at the University of Göttingen (1734–1800)." *Repertorium Eruditorum Totius Europae* 4:1–8.

———. 2021b. "Scholars and Literati at the University of Leiden (1575–1800)." *Repertorium Eruditorum Totius Europae* 5:9–16.

———. 2022a. "Scholars and Literati at the University of Heidelberg (1386–1800)." *Repertorium Eruditorum totius Europae* 6:25–34.

———. 2022b. "Scholars and Literati at the University of Leipzig (1409–1800)." *Repertorium Eruditorum Totius Europae* 8:33–42.

———. 2022c. "Scholars and Literati at the University of Tübingen (1477–1800)." *Repertorium Eruditorum Totius Europae* 7:21–30.

De la Croix, David, and Mara Vitale. 2021a. "Scholars and Literati at the University of Bologna (1088–1800)." *Repertorium Eruditorum Totius Europae* 1:1–10.

———. 2021b. "Scholars and Literati at the University of Padua (1222–1800)." *Repertorium Eruditorum Totius Europae* 3:33–42.

De la Croix, David, and Chiara Zanardello. 2021. "Scholars and Literati at the Academy of Sciences of Turin (1757–1800)." *Repertorium Eruditorum Totius Europae* 5:43–51.

———. 2022. "Scholars at the Royal Academy of Sciences in Paris (1666-1793)." *Repertorium Eruditorum Totius Europae* 9:1–10.

———. 2023. "Scholars and Literati at the University of Modena (1175–1800)." *Repertorium Eruditorum Totius Europae* 9:25–32.

de Teule, Edilbert. 1887. *Chronologie des docteurs en droit civil de l'universite d'Avignon (1303-1791).* Lechevalier.

Debois, Valentine, and David De la Croix. 2021. "Scholars and Literati at the University of Geneva (1559–1800)." *Repertorium Eruditorium Totius Europae* 1:41–47.

———. 2022. "Scholars and Literati at the Royal Dublin Society (1757–1800)." *Repertorium Eruditorium Totius Europae* 6:9–15.

de Coste, Hilarion. 1648. *La vie du RP Marin Mersenne, théologien, philosophe et mathématicien de l'Ordre des pères minimes*. chez Sebastien Cramoisy, imprimeur ordin. du Roy, & de la Reyne régente.

Defrasne, Jean, Jean-Pierre Maurat, Marie Lordereau, and François Lassus. 2002. "Deux cent cinquantième anniversaire de l'académie de Besançon." Besançon.

De la Croix, David. 2021. "Scholars and Literati at the University of Copenhagen (1475–1800)." *Repertorium Eruditorum Totius Europae* 2:21–29.

Delen, Första, and Martin Johan Julius Weibull. 1868. *Lunds Universitets Historia, 1668-1868*. Lund: CWK Gleerup.

De Lens, Louis. 1880. *Université d'Angers, du Xve siècle à la Révolution française*. Angers: Germain et Grassin.

Del Negro, Piero. 2015. *Clariores: dizionario biografico dei docenti e degli studenti dell'Università di Padova*. Padova: Padova University Press.

Deloume, Antonin. 1890. "Personnel de la Faculté de droit de Toulouse depuis la fondation de l'Université de Toulouse au XIIIe siècle." manuscript.

Denéchère, Yves, and Jean-Michel Matz. 2012. *Histoire de l'Université d'Angers du Moyen Age à nos jours*. Presses universitaires de Rennes.

de Pontville, Michel. 1997. "Histoire de l'Académie de Caen." manuscript.

De Renzi, Salvatore. 1857. *Storia documentata della Scuola medica di Salerno*. Nobile.

Di Caprio, Giovanni, and Mara Vitale. 2021. "Scholars and Literati at the University of Fermo (1585–1800)." *Repertorium Eruditorum Totius Europae* 5:27–34.

Dittmar, Jeremiah. 2019. "Economic Origins of Modern Science: Technology, Institutions, and Markets." London School of Economics.

Dominique, Saint-Pierre. 2017. *Dictionnaire historique des Académiciens de Lyon (1700–2016)*. Lyon: Editions de l'Académie.

Dorsman, Leen. 2011. "Catalogus Professorum Academiae Rheno-Traiectinae."

Drüll, Dagmar. 1991. *Heidelberger Gelehrtenlexikon: 1652–1802*. Berlin, Heidelberg: Springer Berlin Heidelberg.

———. 2002. *Heidelberger Gelehrtenlexikon 1386–1651*. Berlin, Heidelberg: Springer Berlin Heidelberg and Imprint and Springer.

Duhamel, Leopold. 1895. "Liste des primiciers de l'Université d'Avignon." Archives du Vaucluse.

Duijnstee, Marguerite. 2010. "L'enseignement du droit civil à l'université d'Orleans du début de la guerre de Cents ans (1337) au siège de la ville (1428)." Ph.D. diss., Institute of Private Law, Faculty of Law, Leiden University.

Dulieu, Louis. 1975. *La médecine à Montpellier, vol I: Le Moyen Âge*. Avignon: Les presses universelles.

———. 1979. *La médecine à Montpellier, vol II: La Renaissance*. Avignon: Les presses universelles.

———. 1983. *La médecine à Montpellier, vol III: L'âge classique*. Avignon: Les presses universelles.

Ebel, Wilhelm, ed. 1962. *Catalogus professorum Gottingensium: 1734 - 1962*. Göttingen: Vandenhoeck & Ruprecht and Niedersächsische Staats- und Universitätsbibliothek.

Emden, Alfred Brotherston. 1959. *A Biographical Register of the University of Oxford to AD 1500*. Volume 3. Clarendon Press.

Ercolani, Giovanni Battista. 1881. *Accademia delle Scienze dello Istituto di Bologna dalla sua origine a tutto il 1880*. Bologna.

Fabroni, Angelo. 1791. *Historiae academiae pisanae*. Bologna: Forni.

Facciolati, Jacopo. 1757. *Fasti Gymnasii Patavini Jacobi Facciolati studio atque opera collecti: Fasti gymnasii Patavini Jacobi Facciolati opera collecti ab anno 1517 quo restitutae scholae sunt ad 1756*. Volume 1. typis Seminarii.

Fagereng, Andreas, Magne Mogstad, and Marte Ronning. "Why do wealthy parents have wealthy children?" *Journal of Political Economy*, forthcoming.

Feenstra, Robert, Margreet Ahsmann, and Theo Johannes Veen. 2003. *Bibliografie van hoogleraren in de rechten aan de Franeker Universiteit tot 1811*. Amsterdam: Koninklijke Nederlandse Akademie van Wetenschappen.

Ferté, Patrick. 1975. *L'université de Cahors au XVIIIe siècle: 1700-1751*. Saint-Sulpice-la-Pointe: Ferté.

Flessa, Dorothee. 1969. "Die Professoren der Medizin zu Altdorf von 1580 - 1809." Ph.D. diss., Universität Erlangen-Nürnberg.

Fleury, Georges, and Auguste Dumas. 1929. *Histoire de l'ancienne Université d'Aix de 1730 à 1793: d'après des documents inédits*. Nicollet.

Forestié, Émerand. 1888. La Société littéraire et l'ancienne académie de Montauban.

Foster, Joseph. 1891. *Alumni Oxonienses: the members of the university of Oxford, 1500-1886*. London: Parker.

Fournier, Marcel. 1892. *Histoire de la science du droit en France*. Librairie du recueil général des lois et des arrêts et du journal du palais.

Frova, Carla, Giuliano Catoni, and Paolo Renzi. 2001. "Maestri e scolari a Siena e Perugia 1250-1500."

Frova, Carla, and Stefania Zucchini. 2017. "Onomasticon: una banca dati per la storia dell'Università di Perugia." *Annali di Storia delle università italiane* 21 (1): 117–134.

Gaullieur, Ernest. 1874. *Histoire du Collége de Guyenne d'après un grand nombre de documents inédits*. Paris: Sandoz et Fischbacher.

Gherardi, Alessandro. 1881. *Statuti dell'Università e studio fiorentino dell'anno MCCCLXXXVII*. Volume 9. Forni editore.

Gkopi, Anna Maria, and Robert Stelter. 2023. "Scholars and Literati at the University of Freiburg (1457–1800)." *Repertorium Eruditorum Totius Europae* 9:59–68.

Gleixner, Ulrike. 2019. "Professorenkatalog."

Grant, Alexander. 1884. *The story of the University of Edinburgh during its first three hundred years*. London: Longmans, Green, and Company.

Grünblatt, Serge. 1961. "Les chirurgiens de l'Hôtel-Dieu de Nantes sous l'ancien régime: Esquisse d'histoire de la médecine à Nantes au 18ème siècle." Ph.D. diss., Nantes.

Gualandris, Tifenn, and Mara Vitale. 2023. "Scholars and Literati at the University of Pisa (1343–1800)." *Repertorium Eruditorum Totius Europae* 11:25–33.

Güell, Maia, Michele Pellizzari, Giovanni Pica, and José V Rodríguez Mora. 2018. "Correlating social mobility and economic outcomes." *The Economic Journal* 128 (612): F353–F403.

Güell, Maia, José V. Rodríguez Mora, and Christopher I. Telmer. 2015. "The Informational Content of Surnames, the Evolution of Intergenerational Mobility, and Assortative Mating." *The Review of Economic Studies* 82 (2): 693–735.

Gundlach, Franz, and Inge Auerbach. 1927. *Catalogus professorum academiae Marburgensis; die akademischen Lehrer der Philipps-Universität in Marburg*. Marburg: N. G. Elwert'sche Verlagsbuchhandlung G. Braun.

Günther, Johannes. 1858. *Lebensskizzen der Professoren der Universität Jena seit 1558 bis 1858*. Jena: Friedrich Mauke.

Gunther, Robert T. 1925. *Early Science in Oxford, Vol. iv The Philosophical Society*. Oxford.

Hänsel, Willy. 1971. *Catalogus Professorum Rintelensium*. Rinteln: Verlag.

Harbury, Colin, and David Hitchins. 1979. *Inheritance and Wealth Inequality in Britain*. London: Allen and Unwin.

Haupt, Herman, and Georg Lehnert. 1907. *Chronik der Universität Giessen, 1607 bis 1907*. Verlag von Alfred Töpelmann.

Hazon, Jacques Albert, and Thomas Bernard Bertrand. 1778. *Notice des hommes les plus célèbres de la Faculté de Médecine en l'Université de Paris, depuis 1110, jusqu'en 1750 (inclusivement)*. chez Benoît Morin.

Hertz, Tom, Tamara Jayasundera, Patrizio Piraino, Sibel Selcuk, Nicole Smith, and Alina Verashchagina. 2007. "The inheritance of educational inequality: International comparisons and fifty-year trends." *The BE Journal of Economic Analysis & Policy* 7, no. 2.

Herzog, Johann Werner. 1780. *Adumbratio eruditorum Basiliensium meritis apud exteros olim hodieque celebrium: Adpendicis loco Athenis Rauricis addita*. sumtibus Car. Aug. Serini, bibliopolae.

Hirsch, Theodor. 1837. *Geschichte des academischen Gymnasiums in Danzig, in ihren Hauptzügen dargestellt*. Volume 1. Wedelschen Hofbuchdruckerei.

Holmlund, Helena, Mikael Lindahl, and Erik Plug. 2011. "The Causal Effect of Parents' Schooling on Children's Schooling: A Comparison of Estimation Methods." *Journal of Economic Literature* 49 (3): 615–51 (September).

Izarn, Pierre. 1991. "La faculté de médecine de Perpignan au XVIIIe siècle." *Bulletin mensuel de l'Académie des sciences et lettres de Montpellier* 22:81–107.

Jantti, Markus, Bernt Bratsberg, Knut Roed, Oddbjorn Raaum, Robin Naylor, Eva Osterbacka, Anders Bjorklund, and Tor Eriksson. 2006. "American exceptionalism in a new light: a comparison of intergenerational earnings mobility in the Nordic countries, the United Kingdom and the United States." IZA discussion paper.

Jaussaud, Philippe, and Édouard-Raoul Brygoo. 2004. *Du Jardin au Muséum en 516 biographies*. Paris: Publications scientifiques du Muséum national d'histoire naturelle.

Jensen, Mikkel Munthe. 2018. "From learned cosmopolitanism to scientific inter-nationalism: the patriotic transformation of Nordic academia and academic culture during the long eighteenth century." Ph.D. diss., European University Institute.

Junius Institute. 2013. "Post Reformation Digital Library – Scholastica." Available at.

Karlberg, Gun. 1977. *Royal Society of sciences of Uppsala, catalogue of publications 1716-1967*. Acta Universitatis Upsaliensis.

Kedzoria, Andrzej. 2021. "Zamiowopedia." https://www.zamosciopedia.pl.

Kiefer, Jürgen DK. 2004. *Bio-Bibliographisches Handbuch der Akademie gemeinnütziger Wissenschaften zu Erfurt 1754-2004*. Akademie Gemeinnütziger Wissenschaften zu Erfurt.

Kiener, Marc, and Olivier Robert. 2005. *Dictionnaire des professeurs de l'Académie de Lausanne (1537-1890)*. Université de Lausanne.

Kirkpatrick, Thomas Percy Claude. 1912. *History of the medical teaching in Trinity College Dublin and of the School of Physic in Ireland*. Hanna and Neale.

Klinge, Matti, Jan-Ivar Lindén, Kerstin Smeds, Rainer Knapas, Anto Leikola, and John Strömberg. 1988. *Helsingfors universitet 1640-1990: Kungliga akademien I Åbo 1640-1808*. Otava.

Kohnle, Armin, and Beate Kusche. 2016. *Professorenbuch der theologischen Fakultät der Universität Wittenberg: 1502 bis 1815/17*. Leipzig: Leucorea-Studien zur Geschichte der Reformation und der Lutherischen Orthodoxie.

Krahnke, Holger. 2001. *Die Mitglieder der Akademie der Wissenschaften zu Göttingen 1751-2001*. Göttingen: Ruprecht Gmbh & Company.

Krüger, Kersten. 2019. "Catalogus Professorum Rostochiensium." http://cphttp://cpr.uni-rostock.de/r.uni-rostock.de/.

Labande, Léon-Honoré. 1901. “Philippe Lauzun. La Société académique d’Agen (1776-1900).” *Bibliothèque de l’École des chartes* 62 (1): 275–276.

Lackner, Franz. 1976. *Die Jesuitenprofessoren an der philosophischen Fakultät der Wiener Universität (1712-1773).* Volume 1. VWGÖ, Verb. d. Wissenschaftl. Gesellschaften Österreichs.

Lamberts, Emiel, and Jan Roegiers. 1990. *Leuven University, 1425–1985.* Leuven: Leuven University Press.

Lamothe-Langon, Etienne-Léon. 1823. *Biographie toulousaine, ou Dictionnaire historique des personnages qui se sont rendus célèbres dans la ville de Toulouse, ou qui ont contribué a son illustration.* Michaud.

Laval, Victorin. 1889. *Histoire de la Faculté de Médecine d’Avignon. Ses origines, son organisation et son enseignement, 1303–1791.* Avignon: Seguin Frères.

Lavillat, Bernard. 1977. *L’enseignement à Besançon au XVIIIe siècle.* Belles Lettres.

Leiden, Universitaire Bibliotheken. 2019. “Leidse hoogleraren vanaf 1575.” https://hoogleraren.leidenuniv.nl/.

Lindahl, Mikael, Mårten Palme, Sofia Sandgren Massih, and Anna Sjögren. 2015. “Long-term Intergenerational Persistence of Human Capital: an Empirical Analysis of Four Generations.” *Journal of Human Resources* 50 (1): 1–33.

Lomholt, Asger. 1950. *Det Kongelige Danske Videnskabernes Selskab 1742—1942.* København: Ejnar Munksgaard.

Maggiolo, Attilio. 1983. *I soci dell’accademia patavina dalla sua fondazione (1599).* Accademia Patavia di Scienze Lettere ed Arti.

Maisonnier, Joseph. 1972. *La Faculté de droit de l’Université de Pau, 1726-1793.* Impr. Y. Cadoret.

Marion, Christophe. 2019. “Annuaire prosopographique : la France savante.” https://www.cths.fr/an/prosopographie.php.

Martin, Eugène. 1891. *L’Université de Pont-à-Mousson (1572-1768).* Paris–Nancy: Berger-Levrault.

Matthew, Henry Colin Gray, Brian Harrison, and R James Long. 2004. *The Oxford dictionary of national biography.* R. James Long.

Maury, Alfred. 1864. *L’ancienne Académie des sciences.* Didier.

Maylender, Michele. 1930. *Storia delle accademie d’Italia.* L. Cappelli.

Mazumder, Bhashkar. 2005. “Fortunate Sons: New Estimates of Intergenerational Mobility in the United States Using Social Security Earnings Data.” *The Review of Economics and Statistics* 87 (2): 235–255.

Mazzetti, Serafino. 1847. *Repertorio di tutti i Professori antichi e moderni della famosa Università, e del celebre Istituto delle Scienze di Bologna.* Bologna: tipografia di San Tommaso d’Aquino.

Mège, F. 1999. *L’Académie des sciences, belles-lettres et arts de Clermont-Ferrand, ses origines et ses travaux.* Académie des sciences de Clermont.

Milsand, Philibert. 1871. *Mémoires de l’Académie des sciences, arts et belles-lettres de Dijon.* Dijon: Imprimerie Rabutot.

Mor, Carlo Guido, and Pericle di Pietro. 1973. *Storia dell’università di Modena.* Volume 1-2. Leo S. Olschki Editore.

Munk, William. 1878. *The Roll of the Royal College of Physicians of London: 1518 to 1700*. Royal College of Physicians of London.

Nadal, Joseph Cyprien. 1861. *Histoire de l'Université de Valence, et des autres établissements d'instruction de cette ville, etc*. Valence: Impr. E. Marc Aurel.

Naragon, Steve. 2006. "Kant in the Classroom: Materials to Aid the Study of Kant's Lectures." Manchester College.

Nève, Félix. 1856. *Mémoire historique et littéraire sur le Collège des trois-langues à l'Université de Louvain, en réponse a la question suivante: Faire l'histoire du collège des trois-langues a Louvain,[...]*. Hayez.

Nolan, John. 2003. *Stable distributions: models for heavy-tailed data*. Birkhauser New York.

Nybom, Martin, and Jan Stuhler. 2019. "Steady-state assumptions in intergenerational mobility research." *The Journal of Economic Inequality* 17 (1): 77–97.

Origlia Paolino, Giovanni Giuseppe. 1754. *Istoria dello studio di Napoli*. Torino: nella stamperia di Giovanni di Simone.

Parodi, Severina. 1983. *Catalogo degli accademici dalla fondazione*. Volume 2. Accademia della Crusca.

Pastor, Julio Rey, and Ernesto García Camarero. 1960. *La cartografía mallorquina*. Instituto Luis Vives.

Pery, Guillaume Marie Auguste Georges. 1888. *Histoire de la Faculté de Médecine de Bordeaux et de l'enseignement médical dans cette ville 1441-1888*. Paris: O. Doin.

Pesenti, Tiziana. 1984. *Professori e promotori di medicina nello studio di Padova dal 1405 al 1509*. Trieste: Lint.

Pietrzyk, Zdzisław, and Jadwiga Marcinek. 2000. *Poczet rektorów Uniwersytetu Jagiellońskiego 1400-2000*. Krakow: Uniwersytetu Jagiellońskiego.

Pillosu, Francesco. 2017. *Libro de grados de Doctores començando del ano 1709 asta 1723*. Università di Cagliari.

Plug, Erik. 2004. "Estimating the effect of mother's schooling on children's schooling using a sample of adoptees." *American Economic Review* 94 (1): 358–368.

Port, Célestin. 1876. *Dictionnaire historique: géographique, et biographique de Maine-et-Loire*. JB Dumoulin.

Prezziner, Giovanni. 1810. *Storia del publico studio e delle società scientifiche e letterarie di Firenze*. Firenze: Carli.

Raggi, A. 1879. Memorie e documenti per la storia dell'Universita di Pavia e degli uomini piu illustri che vi insegnarono.

Ram, Pierre François Xavier de. 1861. *Les quatorze livres sur l'histoire de la ville de Louvain du docteur et professeur en théologie Jean Molanus: Historiae lovaniensium*. Collection de chroniques belges inédites. Bruxelles: Hayez.

Rangeard, Pierre, and Albert Lemarchand. 1868. *Histoire de l'Université d'Angers*. Angers: Barassé.

Renazzi, Filippo Maria. 1803. *Storia dell'Università degli studi di Roma: detta comunemente la sapienza, che contiene anche un saggio storico della letteratura romana, dal principio del secolo XIII sino al declinare del secolo XVIII.* nella stamperia Pagliarini.

Rizzi, Fortunato. 1953. *I professori dell'Università di Parma attraverso i secoli: note indicative bio-bibliografiche*. Tip. fratelli Godi.

Rodrigues, Manuel Augusto. 1992. *Memoria professorum Universitatis Conimbrigensis: 1772-1937*. Coimbra: Arquivo da Universidade de Coimbra.

———. 2003. *Memoria professorum Universitatis Conimbrigensis: 1290-1772*. Coimbra: Arquivo da Universidade de Coimbra.

Rolla, Cecilia, and Mara Vitale. 2022. "Scholars and Literati at the University of Parma (1412–1800)." *Repertorium Eruditorum Totius Europae* 6:1–8.

Rolla, Cecilia, and Chiara Zanardello. 2022. "Scholars and Literati at the Academy of Sciences of Bologna (1714–1800)." *Repertorium Eruditorum Totius Europae* 7:57–65.

Rosen, Josef. 1972. *Die Universität Basel im Staatshaushalt 1460 bis 1535: die Gehälter der Dozenten*. Werner & Bischoff.

Rozier, Jean-François. 1776. *Nouvelle table des articles contenus dans les volumes de l'Académie Royale des Sciences de Paris depuis 1666 jusqu'en 1770*. Paris: Ruault.

Rubio y Borras, Manuel. 1914. *Historia de la Real y Pontificia Universidad de Cervera, dos volúmenes*. Barcelona: Verdager.

Sabbadini, Remigio. 1898. *Storia documentata della Regia Università di Catania*. Catania: Stabilimento Tipografico C. Galàtola.

Sacerdote, Bruce. 2007. "How large are the effects from changes in family environment? A study of Korean American adoptees." *The Quarterly Journal of Economics* 122 (1): 119–157.

Schopferer, Julia. 2016. *Sozialgeschichte der halleschen Professoren 1694–1806: Lebenswege, Netzwerke und Raum als Strukturbedingungen von universitärer Wissenschaft und frühmoderner Gelehrtenexistenz*. Mitteldeutscher Verlag.

Schumann, Eduard. 1892. *Geschichte der Naturforschende Gesellschaft in Danzig*. Danzig: Commissions-Verlag von Wilhelm Engelmann.

Schwinges, Rainer Christoph, and Christian Hesse. 2019. "Repertorium Academicum Germanicum." https://en.rag-online.org/.

Serangeli, Sandro. 2010. *I docenti dell'antica Università di Macerata:(1540-1824)*. Torino: G. Giappichelli.

Shemivot, Vladimir Petrovich. 1873. "General list of members of the Academy of Sciences since its foundation." St. Petersburg.

Sinno, Andrea. 1921. *Diplomi di laurea dell'Almo Collegio salernitano*. Salerno: Stabilimento Tipografico Spadafora.

Slottved, Ejvind. 1978. *Lærestole og lærere ved Københavns Universitet 1537-1977*. Copenhagen: Samfundet for dansk Genealogi og Personalhistorie.

Smart, Robert Noyes. 2004. *Biographical Register of the University of St. Andrews, 1747-1897*. University of St. Andrews Library.

Société de Lausanne. 1790. *Histoire et mémoires de la Société des sciences physiques de Lausanne*. chez Mourer.

Solon, Gary. 1989. "Biases in the Estimation of Intergenerational Earnings Correlations." *The Review of Economics and Statistics* 71 (1): 172–74.

———. 1999. "Intergenerational mobility in the labor market." In *Handbook of Labor Economics*, Volume 3, 1761–1800. Elsevier.

Sturges, RP. 1978. "The Membership of the Derby Philosophical Society, 1783–1802." *Midland History* 4 (3): 212–229.

Svatoš, Michal, and Ivana Čornejová. 1995. *Dějiny Univerzity Karlovy: 1348-1990*. Karolinum.

Taillefer, Michel. 1985. *Une académie interprète des Lumières: l'Académie des sciences, inscriptions et belles-lettres de Toulouse au XVIIIe siècle*. Paris: Editions du CNRS.

Tersmeden, Fredrik. 2015. "Rektoratet vid Lunds universitet - några historiska glimtar." Rektorsinstallation Lunds universitet 28 januari 2015.

The British Library. 2021. Database of Italian Academies.

Tola, Pasquale. 1837. *Dizionario biografico degli nomini illustri di Sardegna*. Chirio.

Torrecilla, Luis Miguel Gutiérrez, Manuel Casado Arboniés, and Pedro L Ballesteros Torres. 2013. *Profesores y estudiantes: biografía colectiva de la Universidad de Alcalá (1508-1836)*. Universidad de Alcalá, Servicio de Publicaciones.

University of Glasgow. 2020. "The University of Glasgow Story." https://universitystory.gla.ac.uk.

Vallauri, Tommaso. 1875. *Storia delle università degli studi del Piemonte*. Stamperia reale di GB Paravia.

van Epen, Didericus Gysbertus. 1904. *Album studiosorum Academiæ gelro-zutphanicae MDCXLVIII-MDCCCXVIII*. The Hague: Jacobum Hoekstra .

Venn, John. 1922. *Alumni Cantabrigienses: a biographical list of all known students, graduates and holders of office at the University of Cambridge, from the earliest times to 1900*. Cambridge University Press.

Vidal y Díaz, Alejandro, et al. 1869. *Memoria histórica de la Universidad de Salamanca*. Salamanca: Imprenta de Olivo y Hermano.

Vitale, Mara. 2022a. "Scholars and Literati at the University of Pavia (1361–1800)." *Repertorium Eruditorum Totius Europae* 8:45–52.

———. 2022b. "Scholars and Literati at the University of Salamanca (1218–1800)." *Repertorium Eruditorum Totius Europae* 7:1–10.

———. 2023. "Scholars and Literati at the Accademia della Crusca (1583–1800)." *Repertorium Eruditorum Totius Europae* 10:39–45.

Volbehr, Friedrich, and Richard Weyl. 1956. *Professoren und Dozenten der Christian-Albrechts-Universität zu Kiel: 1665 - 1954*. Kiel: Hirt.

von Aschbach, Joseph Ritter. 1865. *Geschichte der Wiener Universität und Ihre Gelehrten*. Verlag der Universität.

Von Bahr, Gunnar. 1945. *Medicinska fakulteten i Uppsala*. Stockholm: Almqvist & Wiksell International.

von Hehl, Ulrich, and Thomas Riechert. 2017. "Catalogus professorum lipsiensium." https://research.uni-leipzig.de/catalogus-professorum-lipsiensium/.

Wachter, Clemens. 2009. *Philosophische Fakultät, Naturwissenschaftliche Fakultät*. Erlanger Forschungen Sonderreihe. Erlangen: Universitätsbibliothek Erlangen and Universitätsbund Erlangen-Nürnberg.

Walker, Thomas Alfred. 1927. *A Biographical Register of Peterhouse Men*. Cambridge: Cambridge University Press.

Walter, Ludwig K. 2010. *Dozenten und Graduierte der Theologischen Fakultät Würzburg 1402 bis 2002*. Quellen und Forschungen zur Geschichte des Bistums und Hochstifts Würzburg. Würzburg: Schöningh.

Waterston, C D, and A Macmillan Shearer. 2006. "Former Fellows of the Royal Society of Edinburgh." PDF document.

Wilde, WR, and Owen Lloyd. 1844. "Memoir of the Dublin Philosophical Society of 1683." *Proceedings of the Royal Irish Academy (1836-1869)* 3:160–176.

Wolff, Helmut. 1973. *Geschichte der Ingolstädter Juristenfakultät: 1472-1625*. Volume 5. Freiburg: Duncker und Humblot.

Zanardello, Chiara. 2022a. "Literati and Scholars at the Pilosophical Society of Oxford (1651-1690)." *Repertorium Eruditorum Totius Europae* 8:17–25.

———. 2022b. "Scholars and Literati at the University of Turin (1404–1800)." *Repertorium Eruditorum Totius Europae* 7:31–38.

Zucchini, Stefania. 2008. *Universita e dottori nell'economia del Comune di Perugia: i registri dei Conservatori della moneta (secoli 14.-15.)*. Deputazione di storia patria per l'Umbria.
